# Geochemical Assessment of Sediment Heavy Metal(loid) Concentrations in Lofa County, Northwestern Liberia: A Comparative Analysis of Average Shale and Upper Continental Crust Background Values

**DOI:** 10.3390/toxics14050436

**Published:** 2026-05-14

**Authors:** Hafizou M. Sow, Quanrong Wang, Mohamed Hussein Yousif, Fred B. Wright, Kaixu Chen, Chong Chen, Abara A. Biabak Indrick

**Affiliations:** 1 School of Environmental Studies, China University of Geosciences, Wuhan 430074, China; 2 MOE Key Laboratory of Groundwater Quality and Health, China University of Geosciences, Wuhan 430078, China; 3 School of Marine Science and Technology, China University of Geosciences, Wuhan 430074, China; 4 Wuhan Center, China Geological Survey, Wuhan 430205, China

**Keywords:** geochemical background, artisanal gold mining, ecological risk, West Africa

## Abstract

Despite the proliferation of mining and industrial activities within the Mano River Union member states, sediment quality assessment remains limited due to the lack of a comprehensive geochemical dataset. To narrow this knowledge gap, we evaluated heavy metal(loid) concentrations in stream sediments from Lofa County, Liberia. A total of 313 samples were collected and analyzed for eight metal(loid)s (Cu, Pb, Zn, Cr, Cd, Ni, As, and Hg). The contamination factor (CF), enrichment factor (EF), geoaccumulation index (Igeo), and potential ecological risk index (PERI) were calculated independently against two background values: the average shale and upper continental crust (UCC) values. The UCC background values proved more appropriate than average shale for Liberia’s geographic location and geological setting, providing results that align with the empirical data. The results show that zinc concentrations were consistently low across all sampling sites, indicating regional depletion of the micronutrient. Despite variations in the methodological approaches, assessment results from all four indices identified mercury and arsenic as the contaminants of primary concern. The varying degrees of metal(loid) enrichment and depletion necessitate further research in the study area. This study should guide policymakers in devising a sustainable plan for tackling site-based contamination and delivering on the United Nations Sustainable Development Goal 6.3.

## 1. Introduction

There is a need for the establishment of regional sediment quality guidelines that will align with the United Nations Sustainable Development Goal (SDG 6.3) amongst the Mano River Union member states. Despite numerous studies on sediment quality conducted in West Africa and other parts of the world, very little attention has been given to the sediment quality in western Liberia, primarily due to the unavailability of geochemical data, creating a significant knowledge gap in sediment quality analysis within the Mano River Union (MRU) region (encompassing Liberia, Sierra Leone, Guinea, and the Ivory Coast).

Mining activities do not differ much in the region, owing to the fact that the region shares similar geology. However, though there might be slight variations in mining approaches due to local regulations, overall, mining activities are similar in the region. Historically, mining activities have been ongoing in the MRU region for several decades, consequently releasing loads of potentially toxic elements (PTEs) in streams and rivers. Recent findings by [1] reveal significant heavy metal(loid) contamination in the Fatala River basin in the Republic of Guinea, highlighting arsenic, cadmium, aluminum, tungsten, and iron confined primarily to the Sangardi gold mining districts and the Fria bauxite mining complex. Also, a study in the Pampana River (Sierra Leone) found that the primary source of metal(loid)s in the river was due to gold mining activities at the river’s headwaters (Lake Sonfon). Chromium, cobalt, lead, and thorium were reported to have high mean concentrations in soil at Lake Sonfon, while mercury concentration in 87% of the total fish samples collected in the vicinity exceeded the FDA (Food and Drug Administration)-recommended threshold [2]. A similar study was conducted in the vicinity of the Tongon Mine in Cote d’Ivoire, with results indicating high concentrations of arsenic proximal to dumps and pits [3]. These studies provide a broader perspective of the prevalence of heavy metal(loid)s in the region, and their consequences in these resource-rich districts.

Recent studies have proven that high concentrations of Hg and As in the environment are largely associated with anthropogenic activities. For example, in Sub-Saharan Africa, artisanal and small-scale gold mining activities in poorly monitored regions contribute up to 20% of global gold production, which uses up to 496 tons of Hg annually [4,5]. Artisanal and small-scale gold mining activities often involve the use of mercury in the gold amalgamation process, which subsequently leaches into surrounding rivers, streams, and groundwater [6]. Similarly, arsenic is a ubiquitous PTE naturally present in geological formations, predominantly associated with Au ores [7,8]. The immobilization of these As-bearing sulfide veins during gold extraction processes and the weathering of exposed tailings are two potential sources of arsenic release in the environment [8,9].

While it is a well-established fact that the enrichment of some metal(loid)s in sediments may have an adverse effect on the environment, it is noteworthy to point out that some trace metals, including Zn, are important micronutrients, and their absence in geological units or as a result of leaching is crucial for regional sediment quality, which necessitates environmental management plans [10,11]. The deficiency of zinc in sediments imposes several constraints on aquatic ecosystem stability and productivity [12,13]. In zinc-deficient sediments, benthic primary producers such as microphytes and periphyton experience marked physiological stress that alters the nutritional quality of the base of the food web [14].

Assessing the concentrations of trace heavy metal(loid)s in sediments relies on the selection of the appropriate background values. However, despite numerous studies, the determination of the most appropriate background value in the assessment of sediment heavy metal(loid)s still remains ambiguous. The ill-selection of an inappropriate background value can result in erroneous conclusions. For example, applying the upper continental crust background values for a study area underlain by distinctive local shale geology can introduce significant bias into the assessments. In the same vein, applying the average shale background values to regions with distinct lithologies where the local geology is lower than the average shale values will underestimate pollution indices, potentially masking genuine contamination [15].

This study attempts to bridge the knowledge gap in the Mano River Union region, focusing on western Liberia (Lofa County), and aims to assess the concentration of metal(loid)s in sediments as a means of delivering on the United Nations Sustainable Development Goal (SDG 6.3). By independently using various mathematical models against both the upper continental crust (UCC) proposed by [16] and average shale background values proposed by [17], the objective of the research is to: 1. Evaluate the most appropriate background values for the region; 2. evaluate the enrichment and deficiency levels of trace elements (metal(loid)) in sediments; and 3. assess the ecological risk of heavy metal(loid) pollution.

## 2. Study Area

**Figure 1:**
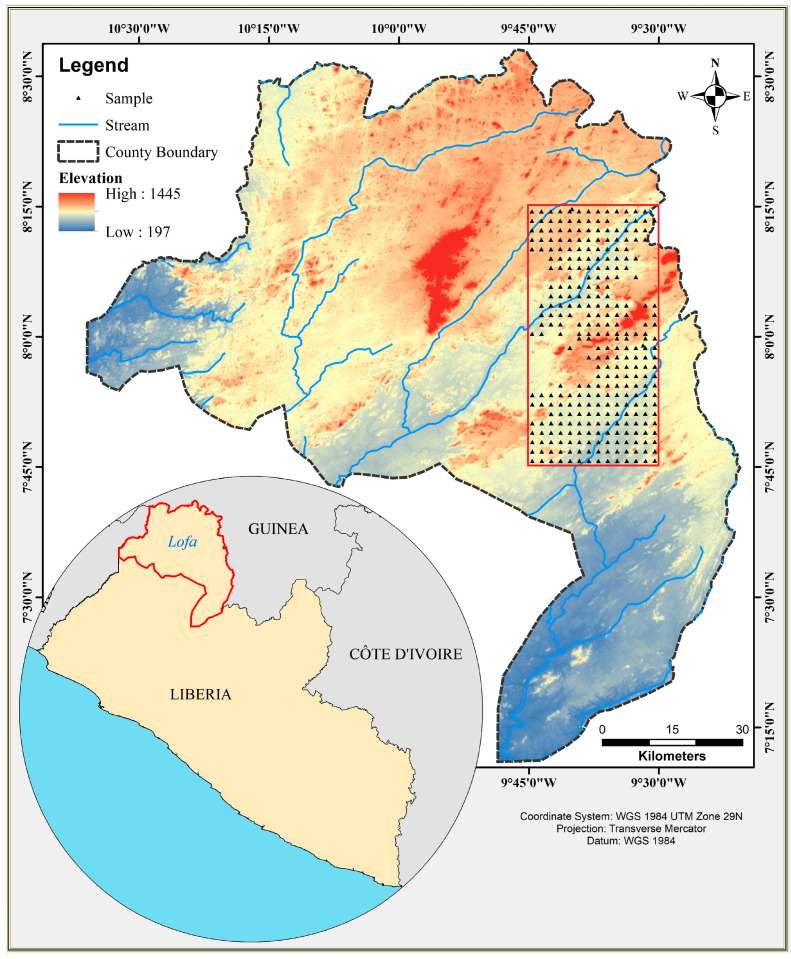
Study area location map and sample points.

Samples were collected from northwestern Liberia, Lofa County (Figure 1). The county covers an area approximately 9982 km^2^ (3854 sq. miles) with a population of about 367,376. The annual rainfall in the county varies and may range from 2700 mm to 3457 mm, with the highest amount of rainfall received in the months of July and August. Agriculture is the primary occupation (accounting for about 71.5% of all activities) of local residents and the main source of livelihood in the county. In recent decades, some residents have shifted from traditional agricultural activities to mining as a means of diversification and tapping into the region’s vast, underexplored natural resources [18]. Artisanal small-scale gold mining (specifically, alluvial panning using mercury amalgamation) and diamond mining are especially common in Lofa County. And across the MRU region, mining practices remain largely similar due to their shared geological characteristics.

The region forms part of the West African Guinean Shield and the Liberian Age Province (2900 Ma) [19]. Bedrock in the region comprises leucocratic granitic rocks, ranging from granite to quartz diorite in composition, with a massive granitic texture. Iron formation, amphibolite, schist, and quartzite form lenses and stretches between and within gneissic units. Gneisses and a few other metamorphic rocks form the underlying bedrock.

Igneous rocks in the region are a conglomeration of granites, diabase dikes, plugs (consisting mainly of olivine, clinopyroxene, tremolite, and anthophyllite), norites, and sills that form part of the crystalline basement. Larger ultramafic intrusions in some areas are completely altered to talc and soapstone. Granitic rocks are mainly massive to coarse-grained rocks, chiefly granite and granodiorite, but range to quartz diorite in composition. The dikes are vertical and up to 50 m thick, dating back 2.8 b.y., exhibiting an eastward trend, with older dikes being metamorphosed to amphibolite. Virtually all rocks in the area were once exposed to regional metamorphism that was accompanied by a varying degree of deformation, evident by the abundant isoclinal folds in most of the schists and gneisses. The regional stratigraphy reflects the dominant northeast-trending grain of the Precambrian rocks, with several linear features trending east-northeast [19].

Most major faults are sub-parallel to regional stratigraphic trends and have probably had a major influence on the region’s topography. Faults in the region follow the regional structural trends, and major faults are discernible by river channels. High-angle, east-trending faults are oblique to the regional stratigraphy and physiographic trends and appear to be younger than the strike-slip faults [20,21]. The geological map of Lofa County is presented in Figure S1 of the Supplementary Information.

## 3. Materials and Methods

### 3.1. Sample Collection and Analysis

Field work was conducted between February and May 2016, in a carefully selected area of 1516.9 km^2^ of the county’s total area (referred to as “Lofa Key Districts”). The 1516.9 km^2^ area was divided into blocks of 4 km^2^, with each 4 km^2^ block further sub-divided into four smaller cells of 1 km^2^ to serve as a catchment area for one to two samples.

Samples were collected from active stream beds and from areas where obstacles in the stream had piled up sediments. Third-order streams were sampled for wider control areas from three to four locations about 15 m to 25 m along active stream lines and composited to obtain one representative sample of the catchment area of each sampling point.

Sand and silt were the main sampling media. Samples were sun-dried and placed into a 2 mm sieve. A minus 2 mm mesh fraction was collected into a 0.3 mm sieve. About 1 kg of the minus 2 mm mesh size was collected and placed into a labeled calico bag. Grain size greater than 1 mm accounted for more than 70% of all grain sizes of the sample.

All samples were analyzed at the laboratory of the Central South Mineral Resources Supervision and Testing Center (People’s Republic of China). As and Hg were analyzed using atomic fluorescence spectrometry (AFS, Jitian, AFS-8130, Beijing, China) while Cu, Pb, Zn, Ni, Cd, and Cr were analyzed using inductively coupled plasma mass spectrometry (ICP-MS, PerkinElmer Nexion-350D, Shelton, CT, USA). Mn was analyzed using X-ray fluorescence (XRF, Rigaku ZSX100e, Tokyo, Japan). Analysis accuracy and precision were monitored using national primary standard reference materials (SRMs) from the Chinese Academy of Measurement Sciences. Three GBW stream sediment SRMs (GBW07361, GBW07304a, and GBW07309) were inserted into the samples to be analyzed and analyzed under the same conditions as the routine samples fourteen (14) times. Full and detailed laboratory procedures are presented in Supplementary Information (SI-1), element detection limits and reportability rate are reported in Table S1, and the certified values, determined values, and recovery rate of the reference materials are presented in Table S2.

### 3.2. Sediment Quality Guidelines

Sediment quality guidelines (SQGs) have been used in a variety of applications, ranging from designing monitoring programs to conducting remedial investigations and ecological risk assessments [22]. Two threshold (upper and lower) limits are established to indicate different levels of toxicity: the threshold effect concentration and probable effect concentration.

The threshold effect concentration or TEC (i.e., the concentration value below which adverse effects on living organisms are unlikely to be noticed) includes the effect range low (ERL) [23], threshold effect levels (TEL) [24], lowest effect levels (LEL) [25], and minimal effect thresholds (MET) [26]. The probable effect concentration (PEC)—the concentration level at which adverse effects are more likely to be seen and felt—includes probable effect levels (PELs) [24], effect range median (ERM) [23], severe effect levels (SEL) [25] and toxic effect thresholds (TET) [26]. Since regulations regarding sediment quality differ from country to country within the MRU region, and the methodological approach mostly depends on the scope of work, the TEL and PEL proposed by [24] were adopted in this study.

### 3.3. Background Values

In this study, the upper continental crust (UCC) and average shale values were independently used to determine the most fitting background values for the region and to cross-validate our research findings. UCC values of all metal(loid)s except Hg were adopted from [16], while Hg was adopted from [27]. Average shale values of all metal(loid)s were adopted from [17].

### 3.4. Data Preparation and Heavy Metal(loid) Concentration Assessment

Microsoft Excel was used to perform logical functions. Descriptive analyses and graphical representations of the data were done using Anaconda’s Jupyter Notebook 6.5.4 package. Location and density maps were done in ArcMap 10.4.1. Pearson correlation matrix (PC), Spearman rank correlation, and violin plots were done in Origin pro 2026.

Mathematical indices were used to quantify sediment metal(loid) concentrations in the study area. Density maps and heat maps were used to identify metal contamination hotspots, and the Pearson correlation matrix and Spearman rank correlation were used to understand the linkage between parameters. The dataset was carefully evaluated for NaNs (not a number) and measurements that were below detection limits (BDLs), and rows containing BDLs were removed in their entirety.

To assess heavy metal(loid) concentration (i.e., enrichment and deficiency) levels in sediments in the Lofa Key Districts, the contamination factor (CF), enrichment factor (EF), geoaccumulation index, and potential ecological risk index (PERI) were incorporated.

#### 3.4.1. Contamination Factor (CF)

The contamination factor quantifies the degree of sediment contamination by measuring the metal concentration of sediment in an area relative to its preindustrial background concentration levels [28]. It is expressed as the ratio of a metal concentration in a sediment sample to its preindustrial value. The CF is presented as:

(1) $$\mathrm{CF}=\frac{Cn}{C\;background},$$

where *Cn* is the metal concentration in the sample and *C background* is the background or preindustrial value of the metal being analyzed. CF < 1 (Low Contamination), 1 < CF < 3 (Moderate Contamination), 3 < CF < 6 (Considerable Contamination), and CF > 6 (High Contamination).

#### 3.4.2. Enrichment Factor (EF)

The enrichment factor represents the average concentration of heavy metal(loid)s. It is obtained by dividing the sediment metal concentration by the background value of the metal being evaluated. It tells the degree to which sediments are enriched with a metal pollutant [28]. Mathematically, the *EF* is expressed as:

(2) $$EF=\frac{\left(\frac{M}{Mn}\right)Sample}{\;\;\left(\frac{M}{Mn}\right)Background},$$

where $\left(\frac{M}{Mn}\right)Sample$ is the ratio of the concentration of the element and that of Mn in the sediment, and $\left(\frac{M}{Mn}\right)Background$ is the ratio of the concentration of the element and that of Mn in an unpolluted background. EF < 2 (Deficiency to Low Enrichment), EF 2–5 (Moderate Enrichment), EF 5–20 (Significant Enrichment), EF 20–40 (Very High Enrichment), and EF > 40 (Extremely High Enrichment).

The enrichment factor is important not just in determining the abundance of a metal in sediment samples; it also distinguishes the source of pollution. EF (>0.5 but <1.5) suggests that the trace element or metal concentration is derived from natural processes such as rock weathering. EF (>1.5) signifies that anthropogenic and non-crustal sources account for a significant proportion of the trace element or metal(loid). EFs of metal(loid)s are calculated using some conservative background elements such as aluminum (Al), iron (Fe), and manganese (Mn). Due to the absence of Al and Fe from the dataset, Mn was selected as the conservative background element in this study.

#### 3.4.3. Geoaccumulation Index (Igeo)

The geoaccumulation index is relevant when examining the deficiency or enrichment of metal(loid)s in sediments. The Igeo was specifically introduced in this study to validate the results obtained from the EF. Mathematically, the Igeo can be expressed as:

(3) Igeo = Log_2_ (*C_n_*/1.5*B_n_)

where *C_n_* represents the concentration of the metal in the analyzed sample (n); *B_n_* represents the preindustrial or background value of the same metal; 1.5 accounts for correction for lithological variation. Igeo ≤ 0 (uncontaminated), Igeo 0–1 (uncontaminated to moderately contaminated), Igeo 1–2 (moderately contaminated), Igeo 2–3 (moderately to strongly contaminated), Igeo 3–4 (strongly contaminated), Igeo 4–5 (strongly to extremely contaminated), and Igeo > 5 (extremely contaminated).

#### 3.4.4. Potential Ecological Risk Index

The potential ecological risk index was proposed by [29] to determine the probable risks that an increased concentration of a given metal could pose to the state of the environment. The method integrates two approaches that are sequentially calculated to obtain the overall result. First, the ecological risk factor $\left(Er^{i}\right)$ of each parameter (metal) is calculated using Equation (4):

(4) $$Er^{i}=\sum_{i=1}^{n}\left(Tr^{i}\right)\left(CF\right),$$

where $Er^{i}$ is the ecological risk factor of element *i*, $Tr^{i}$ is the toxic response factor of element *i*, and CF is the contamination factor of the metal under investigation. The toxic response factors for As, Cd, Cr, Cu, Pb, Hg, Mn, Ni, and Zn are given as 10, 30, 2, 5, 5, 40, 1, 5, and 1, respectively [29,30,31].

Second, the potential ecological risk, which is attributed to the cumulative effect of all metal(loid)s in the population, is calculated using Equation (5):

(5) $$\mathrm{RI}=\sum_{i=1}^{n}Er^{i},$$

where RI is the potential ecological risk index. Therefore, one has:

(6) $$\mathrm{RI}=\sum_{i=1}^{n}Er^{i}=\sum_{i=1}^{n}\left(Tr^{i}\right)\left(CF\right),$$

The classification of degree pollution proposed by [29] for $Er^{i}$ and RI is presented in Table 1.

## 4. Results

The concentrations of eight heavy metal(loid)s (Cu, Pb, Zn, Cr, Cd, Ni, As, and Hg) were investigated using stream sediment samples collected from three hundred and thirteen (313) locations in the Lofa Key Districts (LKDs). After removing rows with measurements below detection limits, samples from 309 locations were assessed and comprehensively summarized in Table 2. Heavy metal(loid)s in the study area exhibited distinct enrichment patterns, with zinc and cadmium exhibiting values below the TEL in all sample locations. Copper and arsenic showed degrees of enrichment above the TEL but below the PEL in some locations, while Ni, Cr, Pb, and Hg exhibited values above the PEL at ≥1 site(s).

### 4.1. Assessment of Heavy Metal(loid) Contamination Factor

Table 3 provides a summary of the assessment results evaluated against both average shale and UCC background values. Using average shale values (Table 3a), two locations showed high Pb contamination levels, and one site displayed a high Hg contamination level. The overall contamination degree for the eight heavy metal(loid)s decreased in the order: Hg > Pb > Ni > Cr > Cu > As > Zn > Cd. When considering the UCC background values, six contaminated sites were identified (Table 3b), and the contamination trend was seen in the following order: Hg > As > Pb > Ni > Cr > Cu > Cd > Zn. Detailed results of sediment heavy metal(loid) contamination at all sample locations are provided in Table S3.

### 4.2. Assessment of Heavy Metal(loid) Enrichment Factor

Table 4 summarizes the results of the enrichment factor derived from both average shale and UCC values, which are fully detailed in Table S4. Using average shale as background values (Table 4a), the overall enrichment decreased in the following order: Hg > Pb > Cr > Ni > Cd > As > Zn > Cu, along with the frequency of metal(loid)s. Using the UCC background values (Table 4b), metal(loid) enrichment decreased in the following order: Hg > As > Pb > Ni > Cr > Cd > Zn > Cu, along with the frequency of metal(loid)s. The EF using the average shale values indicates that for Pb, 18.8% of the samples exhibit EF > 5 (i.e., significant enrichment), as opposed to only 13.3% when employing the UCC values. On the contrary, 0.32% and 2.88% of the total samples exhibit significant enrichment with Hg and As, respectively, when using the shale values, as compared to 19.17% and 46.96% of the respective metals when employing the UCC values.

### 4.3. Geoaccumulation Index

As summarized in Table 5, and fully presented in Table S5 of the Supplementary Information, the Igeo was used to evaluate the degree of metal concentration relative to the shale and UCC background values. The results indicate a generally unpolluted environment for most metals, with mean Igeo values below 0. Arsenic emerged as a metal of significant concern, with a mean Igeo value of 0.009, placing it at the threshold of class 1. It is worth mentioning that the observed Igeo for As using the UCC and average shale values were 3.64 and −0.641, respectively. Interpreting these results against the two background values, sediments are considered to be moderately to strongly contaminated by As from a UCC perspective, while uncontaminated from an average shale perspective. Mercury showed distinct localized hotspots with a maximum Igeo of 5.094 (against a UCC background) and 2.258 (against average shale background values), indicating extreme and moderate-to-strong contamination of sediments, respectively.

### 4.4. Assessment of Potential Ecological Risk

Table 6 presents a summary of the calculated ecological risks for eight heavy metal(loid)s in stream sediments. The multi-factor assessment index ensured the comprehensive evaluation of the potential ecological risk of individual heavy metal(loid)s (*Er*) and the ecological risk (*RI*) posed by all eight metal(loid)s due to their cumulative effect.

**Figure 2:**
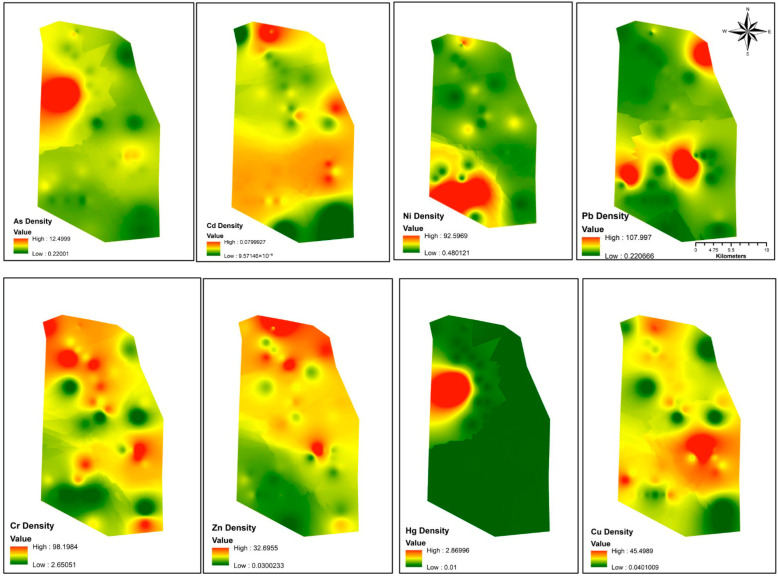
Heavy metal(loid) density maps based on laboratory results.

As previously presented in Table 3, Er values < 40 indicate low potential risk, and RI values <150 indicate low ecological risk. The results show that, with the exception of As and Hg, which pose moderate and very high potential risks, respectively, in LKD-251, all other metals at all sample sites were <40, indicating low potential ecological risk. RI results showed elevated values (>150) in the central western region, correlating with As and Hg hotspots in LKD 251 (Figure 2). RI values in all other locations were <150, indicating low ecological risk (Table S6 of the Supplementary Information).

## 5. Discussion

A comparative analysis of heavy metal(loid) concentrations in the study area against Earth’s crust background values found that zinc concentrations at all sampling locations were below the upper continental crust background values (indicating depletion of the element), and Cd concentration was similar to that of the UCC (suggesting that the metal was neither enriched nor depleted). In contrast, copper, lead, chromium, nickel, arsenic, and mercury showed concentration values above the UCC at different sampling locations. Even though these metal(loid)s exhibited elevated concentrations at different locations, their mean concentrations fell below the global background values for stream sediments, thus suggesting net depletion of the respective metals in the study area.

### 5.1. Spatial Distribution of Heavy Metal(loid)s in Lofa Key Districts

The density maps of heavy metal(loid)s (Figure 2) reveal striking distribution patterns. As and Hg exhibit co-localized high concentrations in the central western part of the study area. Cu and Cr exhibit broadly similar patterns, widely distributed across the study area. Ni displays a distinct distribution gradient, with elevated values concentrated in the south. Though Cd and Zn exhibit concentration levels below the UCC background values, Cd showed relatively elevated concentrations in the southern half and northern edge of the study area, while Zn exhibited higher concentrations in the northern half. Pb is characterized by three isolated hotspots in the southwestern, central, and northeastern regions.

The co-occurrence of mercury and arsenic in the environment is highlighted in numerous studies [32,33,34]. Mercury predominantly occurs as cinnabar (HgS), with minor constituents (<1 wt.%) as impurities in phosphate minerals and iron sulfides such as pyrite and marcasite. Similarly, arsenic is primarily hosted as trace impurities (<1 wt.%) in phosphate minerals and iron sulfides such as pyrite, arsenopyrite, and marcasite [35]. The subsequent weathering and decomposition of these minerals can largely be responsible for the co-availability of arsenic and mercury in an undisturbed environment [36], which generally depends on the solubility of their host minerals, with mobilization rates steadily increasing with decreasing pH.

Arsenic is highly mobile in the subsurface environment, primarily due to redox-driven speciation changes. Unlike on the surface, where arsenic predominantly exists as As (V), in reduced subsurface environments, the metal predominantly exists as As (III), which favors mobility [37]. Ref. [38] demonstrated that arsenic mobility in sediments is largely driven by the formation of soluble thioarsenate with biogenic sulfide, a process mediated by sulfate-reducing bacteria. Additionally, arsenic cycling is influenced by adsorption/precipitation processes as well as microbial and hydrophyte activities [39].

Similar to As, the adsorptive and retentive nature of Hg in sediments makes it a reliable archive for tracking the temporal variation in anthropogenic contamination. Industrial activities tend to produce more bioavailable Hg species compared to mining waste, wherein mercury predominantly exists in inert mineral form. On the contrary, in mining polluted sites, the bulk of the Hg usually exists as insoluble cinnabar (α-HgS). Divalent mercury (Hg(II)) usually represents the predominant form of this metal in sediments, where it forms associations with different fractions depending on geochemical conditions and the presence of organic matter, sulfides, and iron and manganese oxyhydroxides.

Generally, in reduced environments, high concentrations of Hg(0) may be present due to the reduction of Hg(II) to Hg(0), principally mediated by humic acids or Fe(II) [40,41]. On the other hand, in oxic layers of sediments where oxidation of organic matter occurs, mercury forms complexes with iron and manganese oxyhydroxides. CH_3_Hg^+^ is usually formed in sediments under anaerobic conditions influenced by bioavailable Hg through the activity of mainly sulfate-reducing bacteria, which presents a key environmental and human health issue due to the neurotoxicity and bioaccumulation potential. Biotic methylation is strongly influenced not only by the bioavailability of inorganic mercury related to its speciation in sediments, but most importantly, the composition of the microbial community and multi-environmental parameters, including organic matter concentration and quality, speciation of sulfur compounds, and redox conditions.

### 5.2. Heavy Metal(loid) Distribution

**Figure 3:**
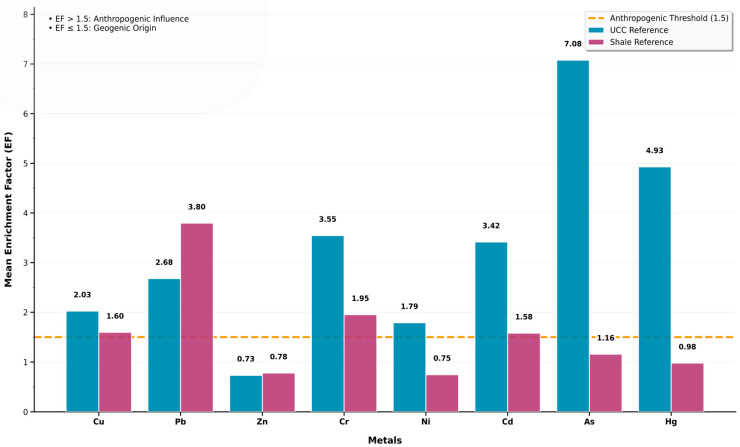
Graphical representation of geogenic and anthropogenic sources of heavy metal(loid) pollution based on mean EF values from UCC and average shale reference system.

Using the mean EF results obtained from both average shale and UCC background values, we graphically distinguished metal(loid)s originating from geogenic sources (weathering of bedrock, etc.) from those influenced by anthropogenic activities (Figure 3). The mean EF values obtained by using the upper continental crust background values indicate that Cu, Pb, Cr, Ni, Cd, As, and Hg are influenced by anthropogenic activities, while Zn is geogenically influenced. On the other hand, results obtained by using average shale background values indicate that Cu, Pb, Cr, and Cd are influenced by anthropogenic activities, and Zn, Ni, As, and Hg are derived from geogenic sources.

The presence of Cu, Pb, Cr, Ni, Cd, As, and Hg in sediments in high concentrations reflects an increasing trend in mining activities, as well as manufacturing and industrialization, the use of fertilizers and pesticides in agricultural processes, and the discharge of domestic waste in streams and river systems [42,43]. Once released, the metal(loid)s get concentrated in rivers, streams, and lakes, eventually enriching sediments that act as long-term sinks for heavy metal(loid)s [44,45].

**Figure 4:**
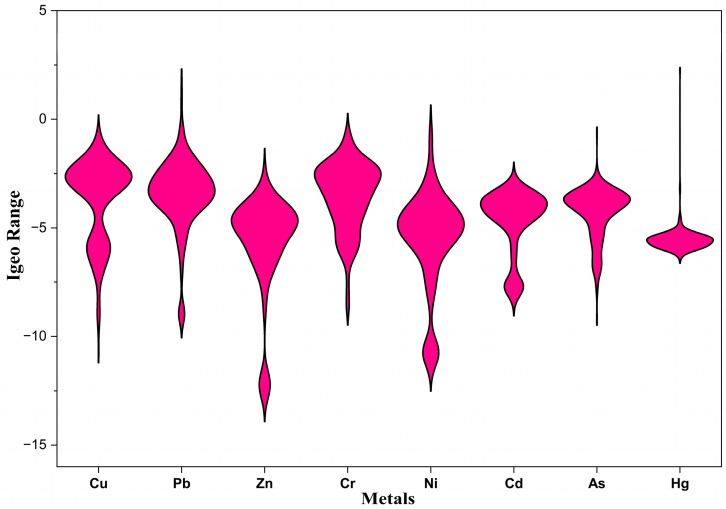
Violin plot of Igeo results against average shale background values.

**Figure 5:**
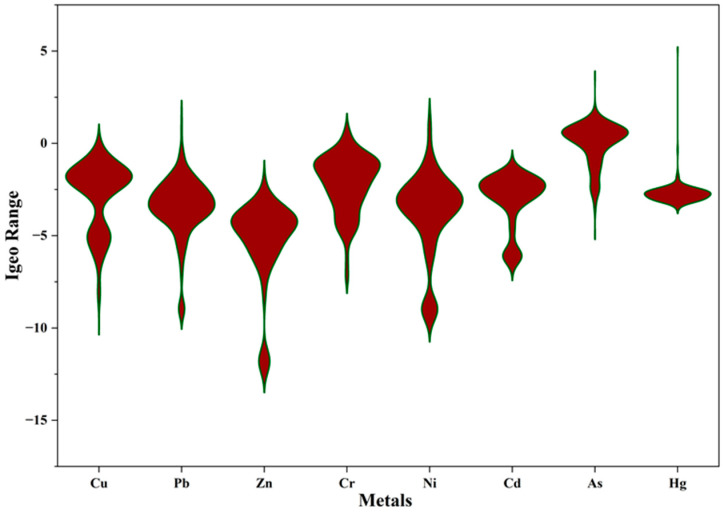
Violin plot of Igeo results against UCC background values.

The Igeo results (Figure 4 and Figure 5) provide a crucial insight: element-independent validation of the pollution pattern suggested by the enrichment factor. Despite overall means of −2.716 (UCC background values) and −5.553 (average shale background values), the results obtained from the Igeo were similar to those of the EF, with a localized hotspot of As and Hg in one location (LKD 251), suggesting point-source pollution. Also, zinc showed deeply negative values, far below natural background values across the study area, strengthening the EF results of zinc deficiency in sediments. The agreement between the two indices confirms that the enrichment signals are real and not an artifact of the normalizer. Manganese, while less ideal as a normalizer, can serve as a functionally adequate substitute in the absence of Al and Fe to identify true contamination hotspots if cautiously used alongside other indices.

Regarding the deficiency of zinc, according to [46,47], sphalerite is the main ore deposit of zinc, and its absence in natural settings can contribute to the low concentrations of the metal in natural environments. Due to the important role of Zn as a micronutrient, the depletion of the metal in sediments in the study area necessitates further investigation and robust mitigation plans. Zinc is required as a trace element at minimal levels for regular metabolic activity. The element plays an essential role both as a structural component of protein and as a co-factor in more than 300 enzymes [48], and its deficiency can lead to loss of appetite, growth retardation, skin morphisms, and immunological abnormalities [49]. According to a study conducted by [50], low availability of Zn in soils can reduce agricultural yield unless appropriately mitigated, highlighting that a population at risk of Zn deficiency is vulnerable to other deficiencies.

The deficiency of zinc in the study area can be attributed to the following factors: 1. The absence of sphalerite (ZnS), the primary natural source of zinc in the environment; and 2. the high solubility nature of Zn in aqueous environments renders it readily soluble, thus promoting its transport as a dissolved ion, preventing it from residing in sediments. Furthermore, the intensity and rate of weathering in tropical regions and metal solubility in aqueous medium render metals such as Zn to be deposited in streams in a solute state that is later precipitated in sediments.

### 5.3. Heavy Metal(loid)s Distribution Based on EF Findings

**Figure 6:**
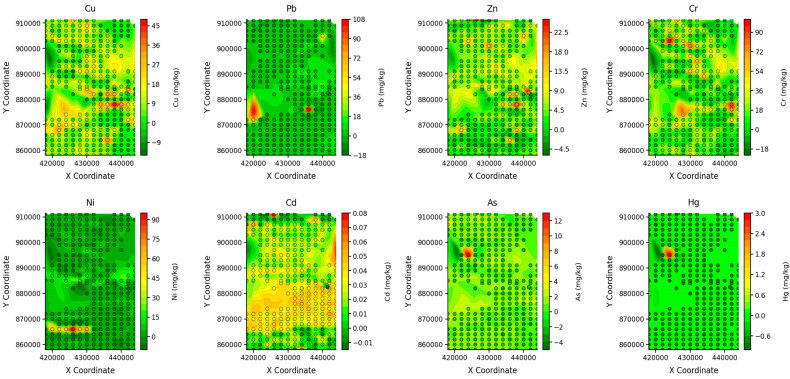
Heavy metal(loid) concentration contour maps showing contamination hotspots based on EF results.

To consolidate our findings, we plotted a spatial distribution map of heavy metal(loid)s based on mean EF values in the region (Figure 6); the map revealed metal-specific hotspots with high As and Hg concentrations in the central western part of the area. Cu was mostly seen in the central and southeastern edge of the study area. Ni was distributed in the south, while Pb was concentrated in the southwest and central parts. High Cr concentration was seen in the southern part of the study area.

### 5.4. Selection of Background Values

The study endeavors to provide homogeneous background values that would pave the way for future work in the MRU region using results obtained in this study. The results of the contamination factor showed a small but significant discrepancy between the two background values (UCC and average shale). One location was found to be extremely contaminated with arsenic when considering the results obtained from the UCC values, but uncontaminated in all sampling locations when evaluated against the average shale values. Lead and zinc exhibited higher EF values in several locations when considering the average shale values, contradicting the laboratory results that indicate a depletion of Zn in sediments. Copper, chromium, nickel, cadmium, arsenic, and mercury exhibited higher values when considering the UCC values.

Using arsenic, copper, chromium, nickel, cadmium, and mercury (underestimated by average shale background values), and zinc and lead (exaggerated by average shale background values), as the basis of our decision (Figure 3), it can be concluded that using UCC as the background values aligns the generated CF, EF, and Igeo results with the empirical dataset (Table S7), as opposed to when considering average shale values. Even though the results obtained by using the UCC background values are slightly exaggerated, they aligned with the analytical data and are productive in environmental engineering and pollution assessment and mitigation programs, since engineers can be assured that contamination is not overlooked. Based on these findings, the UCC values were adopted in this study as background reference values. Hence, the ecological risk factor and ecological risk index were calculated using the UCC’s CF values to have a broader representation of the analytical data.

### 5.5. Correlations Analysis

**Figure 7:**
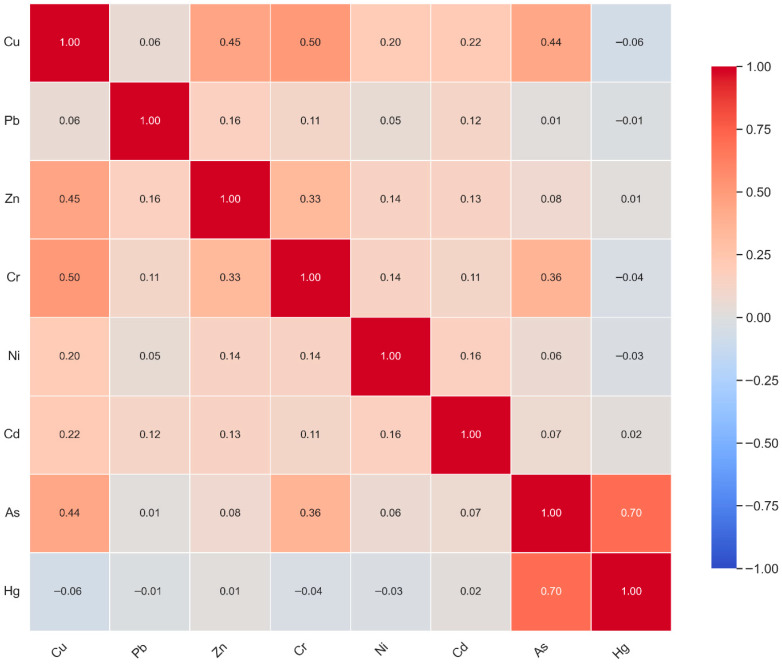
Pearson correlation analysis of heavy metal(loid)s.

Pearson correlation analysis was employed to identify potential similarities in heavy metal(loid) sources and their interrelationships in sediments (Figure 7). According to [51], strong positive correlation coefficients between heavy metal(loid)s suggest either shared pollution sources or similar geochemical behavior between parameters. Correlation results identified strong positive correlations between As-Hg (0.70) and Cr-Cu (0.50), and weak positive correlations were observed between Zn-Cu (0.45), Cu-As (0.44), As-Cr (0.36), and Cr-Zn (0.33). In contrast, negative correlations were observed between Hg-Cu (−0.06), Hg-Pb (−0.01), Hg-Cr (−0.04), and Hg-Ni (−0.03).

**Figure 8:**
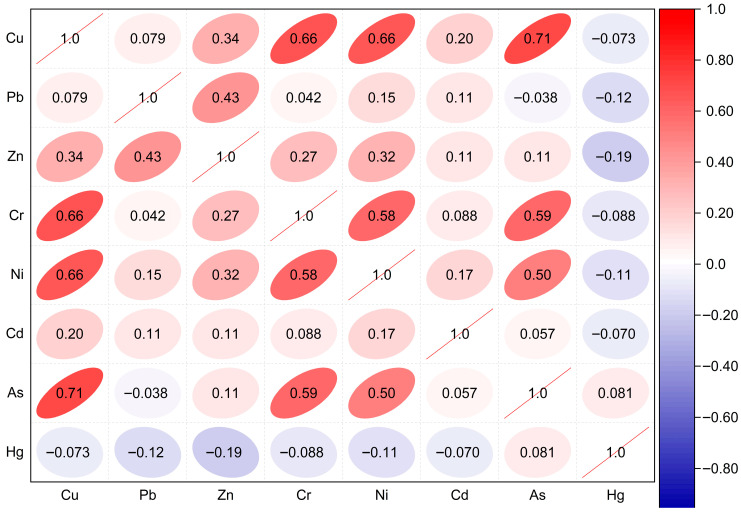
Spearman rank correlation analysis of heavy metal(loid)s.

Owing to the fact that the Pearson correlation coefficient is sensitive to outliers and assumes a normal distribution of the input data, even though geochemical data are frequently non-normally distributed, the Spearman rank correlation was employed since it does not assume data normality and is robust to outliers. Spearman correlation analysis reveals three distinct metal associations that may reflect different source controls (Figure 8). Cu, Cr, Ni, and As form a strongly correlated lithogenic group (ρ = 0.50–0.71). Pb, Zn, and Cd exhibit moderate correlation (ρ = 0.20–0.43), indicating mixed lithogenic and anthropogenic sources. Hg displays a negative correlation with all metals, except As, where a weak positive correlation (ρ = 0.081) is observed, pointing to distinct anthropogenic sources, most likely artisanal small-scale gold mining (ASGM).

Of particular concern is the high concentrations of Hg and As in some localities. According to [52,53], tailing leachates and the use of Hg in artisanal small-scale gold mining activities represent the most recognized sources of As and Hg contamination in downstream sediments, especially in legacy gold mines. The dynamism of As is dependent on its speciation and environmental factors such as temperature, Eh, pH, water composition, physical disturbance, and microbial activity, which also control As mobility from the sediments to the water column. In a separate study, Ref. [54] noted that As release may be controlled by sorption–desorption reactions with clays/phyllosilicates, where As competes for sorption sites with DOC (dissolved organic carbon) and dissolved silica. Arsenic mobility is found to be more extensive in acidic conditions due to the reductive dissolution of Fe-oxyhydroxides and oxidation of pyrite associated with peat layers. In coastal areas, sediments may be potential sources of As in the water column, mainly due to dissolution/desorption reactions of Fe-oxyhydroxides, and should be carefully monitored.

The positive correlations between As and other heavy metals from the two matrices (Pearson and Spearman) indicate that portions of those metals may have originated from a geogenic source. Correlating the findings from the enrichment factor, Pearson correlation matrix, and Spearman rank correlation, we establish that Zn is not influenced by anthropogenic activities, and the negative correlations between mercury and other metals indicate divergent pathways or competitive binding in sediments. This relationship speaks to the fact that Hg is foreign to the environment and was introduced through anthropogenic activities, and the metal has no geogenic origin in the study area.

## 6. Conclusions

The study highlights that average shale background values underrepresent metal contamination in sediments and should be used cautiously, since they offer a more localized reference, which is particularly relevant for fine-grained sedimentary environments. The UCC background values, on the other hand, provide a broader geochemical framework applicable in diverse lithological settings. For scientists and researchers aiming to tackle broader environmental engineering problems and for pollution mitigation purposes, using the UCC as background values will serve well in these aspects, as it does not underestimate metal contamination and enrichment levels.

The research pinpoints the pollution risks of eight heavy metal(loid)s (Cu, Pb, Zn, Cr, Cd, Ni, As, and Hg), which were systematically analyzed and investigated using stream sediment samples from three hundred and nine (309) locations in the Lofa Key Districts. The concentration of zinc at all sampling sites was below the UCC values (which indicates a depletion of the element in the study area), and cadmium concentration was similar to that of the UCC, suggesting that the metal concentration is stable. We attribute the depletion of zinc to the absence of sphalerite and the high solubility of the metal in tropical regions.

Assessment results from all indices identified mercury and arsenic as the contaminants of primary concern, with the potential ecological risk index further associating the high toxicity coefficients of the two metal(loid)s with the high ecological risk (RI) observed in LKD-251.

Employing the Pearson correlation matrix and Spearman’s rank correlation to identify linkages between metal(loid)s, the research found a strong positive correlation (Pearson correlation (r = 0.70)) between arsenic and mercury, suggesting that they may have been derived from a point source. Similarly, Spearman’s rank correlation found a negative correlation between Hg and all metals (with the exception of As). This indicates that Hg was introduced into the environment by anthropogenic activities. However, the co-occurrence of Hg and As in high concentrations at the same location (LKD-251) suggests that the two metal(loid)s are derived from artisanal gold workings where the use of Hg in gold amalgamation and the improper disposal of tailings are prevalent.

The study provides the scientific basis for the adoption of the UCC background values in the establishment of regional sediment quality guidelines within the MRU. In addition to the comprehensive and replicable methodological approach for data-sparse regions, the study directly supports UNSDG 6.3 by identifying priority hazardous metal(loid)s (As and Hg) for monitoring. Furthermore, the Ministry of Agriculture should investigate the impact of Zn deficiency on crop yield and evaluate supplementary programs.

Finally, this study underscores the need for more inclusive studies in the Mano River Union region to bridge the knowledge gap and establish a regional sediment quality guideline that will promote the advancement of UNSDG 6.3 (improve water quality by reducing pollution and minimizing hazardous chemical release).

## 7. Limitations

The results obtained in this study should be considered in the light of certain limitations:

1. Manganese (Mn), though highly sensitive to redox conditions and less-ideal than Al and Fe, was selected as the conservative element for calculating the enrichment factor (EF) in this study. The decision to use Mn in this study stems from the fact that of the three conservation elements (Al, Fe, and Mn), only manganese was available in the dataset, and aluminum and iron were completely absent. However, the Igeo results, which do not require a conservative normalizer, independently confirm the major contamination trends and hotspots as those presented by the EF results. The agreement between the two indices justifies our overall conclusion that Mn, though less ideal, serves as a functionally adequate normalizer for identifying true contamination hotspots in the absence of Al and Fe, and is used in conjunction with other indices.
2. The data presented in this study were obtained through comprehensive and rigorous fieldwork conducted in 2016. Follow-up work was hindered by the scarcity of funds and other unforeseen circumstances. However, these data represent one of the few recent and comprehensive datasets analyzing several potentially toxic elements (PTEs) across a large area extent in the county. The data remain valid for environmental management and policy-devising purposes in the region due to the fact that the geological composition and lithological characteristics, which are responsible for the low zinc concentration, remain unchanged. Also, artisanal small-scale gold mining activities are still prevalent in the county, necessitating the urgent intervention of the requisite authorities to mitigate the issue. Our finding, which establishes that in the absence of local baselines, the UCC provides a more conservative and less misleading benchmark than average shale for this geological setting, is an integral part of this research, which can be useful for future studies.

## Figures and Tables

**Table 1:** Pollution degree classification proposed by [29] for $Er^{i}$ and RI.

$Er^{i}$ (Degree of Pollution)	Classification	RI (Degree of Pollution)	Classification
$Er^{i}$ < 40	Low potential risk	RI < 150	Low ecological risk
$40\leq Er^{i}$ < 80	Moderate potential risk	150 ≤ RI < 300	Moderate ecological risk
$80\leq Er^{i}$ < 160	Considerable potential risk	300 ≤ RI < 600	Considerable ecological risk
$160\leq Er<320^{i}$	High potential risk	RI ≥ 600	Very high ecological risk
$Er^{i}$ ≥ 320	Very high potential risk		

**Table 2:** Descriptive analysis of heavy metal(loid)s (in ppm).

Metal(loid)s	Min.	Max.	Mean	Std. Dev.	Shale.	UCC	TEL	PEL
**Cu**	0.038	45.50	9.0004	7.19651	45	25	35.7	197
**Pb**	0.060	108.00	4.6687	7.91170	20	20	35	91.3
**Zn**	0.027	32.70	4.8149	3.89069	95	71	123	315
**Cr**	0.350	98.20	17.7427	15.39864	90	35	37.3	90
**Ni**	0.055	92.60	4.9927	9.02812	68	20	18	36
**Cd**	0.001	0.08	0.0243	0.01420	0.3	0.098	0.6	3.53
**As**	0.033	12.50	1.2735	0.91469	13	1.5	5.9	17
**Hg**	0.007	2.87	0.0222	0.16157	0.4	0.056 *	0.17	0.486
**Mn**	7.700	260.00	104.73	0.00595	850	600	N/A	N/A

UCC: Upper continental crust value [16]; shale.: Average shale values [17]; * Value obtained from [27]. TEL: Threshold effect level; PEL: Probable effect level.

**Table 3:** Heavy metal(loid) contamination factors using average shale (a) and UCC values (b).

		Cu	Pb	Zn	Cr	Ni	Cd	As	Hg
**CF** **Average Shale**	Maximum	1.01	5.40	0.34	1.09	1.36	0.25	0.96	7.18
	Minimum	0.00	0.00	0.00	0.00	0.00	0.00	0.00	0.02
	Mean	0.20	0.23	0.05	0.20	0.07	0.08	0.10	0.06
	Standard Dev.	0.16	0.39	0.04	0.17	0.13	0.05	0.07	0.40
	Sample with CF > 3	0	2	0	0	0	0	0	1
	% of sample with CF > 3	0	0.64	0	0	0	0	0	0.32
**CF** **UCC**	Maximum	1.82	5.40	0.46	2.81	4.63	0.78	8.33	51.2
	Minimum	0.00	0.00	0.00	0.01	0.00	0.01	0.02	0.13
	Mean	0.36	0.23	0.07	0.51	0.25	0.25	0.85	0.40
	Standard Dev.	0.29	0.40	0.06	0.44	0.45	0.145	0.61	2.88
	Sample with CF > 3	0	2	0	0	2	0	1	1
	% of sample with CF > 3	0	0.64	0	0	0.64	0	0.32	0.32

**Table 4:** Heavy metal(loid) enrichment factors using average shale (a) and UCC values (b).

		Cu	Pb	Zn	Cr	Ni	Cd	As	Hg
**EF** **Average Shale**	Maximum	6.72	57.9	10.3	20.0	16.54	16.1	15.14	112.9
	Minimum	0.02	0.02	0.00	0.02	0.00	0.03	0.07	0.09
	Mean	1.59	3.79	0.78	1.95	0.75	1.58	1.16	0.98
	Standard Dev.	1.01	6.96	1.16	2.12	1.45	2.62	1.44	6.41
	Samples with EF > 5	6	59	4	11	7	30	9	1
	% of sample with EF > 5	1.92	18.8	1.28	3.51	2.24	9.58	2.88	0.32
**EF** **UCC**	Maximum	8.54	40.9	9.76	36.3	39.69	34.9	92.59	569.4
	Minimum	0.03	0.01	0.00	0.04	0.01	0.06	0.43	0.43
	Mean	2.02	2.68	0.73	3.53	1.79	3.42	7.08	4.93
	Standard Dev.	1.28	4.90	1.09	3.82	3.476	5.666	8.83	32.31
	Samples with EF > 5	10	42	3	46	22	54	147	60
	% of sample with EF > 5	3.19	13.3	0.96	14.7	7.03	17.2	46.96	19.17

**Table 5:** Heavy metal(loid) Igeo using both average shale (a) and UCC (b) values.

		Cu	Pb	Zn	Cr	Ni	Cd	As	Hg
**Igeo** **Average Shale**	Max	−0.569	1.848	−2.123	−0.459	−0.139	−2.565	−0.641	2.258
	Min	−10.794	−8.965	−12.365	−8.591	−10.856	−8.435	−9.206	−6.401
	Mean	−3.652	−3.427	−5.711	−3.537	−5.545	−4.669	−4.272	−5.553
	Std. Dev	1.857	1.613	2.251	1.544	2.235	1.442	1.148	0.574
**Igeo** **UCC**	Max	0.279	1.848	−1.703	0.903	1.626	−0.951	3.643	5.094
	Min	−9.946	−8.965	−11.945	−7.228	−9.091	−6.821	−4.921	−3.564
	Mean	−2.804	−3.427	−5.291	−2.174	−3.780	−3.055	0.009	−2.716
	Std. Dev	1.857	1.613	2.251	1.544	2.235	1.442	1.146	0.574

**Table 6:** Heavy metal(loid) potential ecological risk index and ecological risk.

Er									
	Cu	Pb	Zn	Cr	Ni	Cd	As	Hg	RI
Maximum	9.1	27	0.46	5.6	23.1	23.3	83.3	2050	2144.54
Minimum	0.004	0.02	0	0.02	0.013	0.4	0.22	5.0714	13.72
Mean	8	1.17	0.07	1.01	1.248	7.43	8.49	15.86	37.78
Standard Dev.	1.4	1.98	0.05	0.88	2.257	4.35	6.1	115.4	119.90
Samples with $Er^{i}$ > 40	0	0	0	0	0	0	1	3	1
% of sample with $Er^{i}$ > 40	0	0	0	0	0	0	0.32	0.9585	0.319

## Data Availability

The original contributions presented in this study are included in the article/Supplementary Material. Further inquiries can be directed to the corresponding authors.

## Supplementary Materials

The following supporting information can be downloaded at: https://www.mdpi.com/article/10.3390/toxics14050436/s1; Figure S1. Geological map of Lofa County modified from (1). Table S1. Element detection limits. Table S2. Certified values (mg/kg), determined values (mg/kg), and recovery rate (%) of SRMs. Table S3. Heavy metal contamination factors using both average shale and UCC background values. Table S4. Heavy metal enrichment factors using both average shale and UCC background values. Table S5. Geoaccumulation index of heavy metal(loid)s. Table S6. Heavy metal(loid)s Er and RI. Table S7. Laboratory results [55].
