# Supplementary material for: Geochemical Assessment of Sediment Heavy Metal(loid) Concentrations in Lofa County, Northwestern Liberia: A Comparative Analysis of Average Shale and Upper Continental Crust Background Values
Source: Toxics. 2026 May 14;14(5):436. doi: 10.3390/toxics14050436 (PMC13211182; doi:10.3390/toxics14050436)
Supplement: Supplementary file 1 [file toxics-14-00436-s001.zip › toxics-4204232-supplementary.pdf]

Geochemical Assessment of Sediment Heavy Metal(loid) Concentrations in Lofa County, Northwestern Liberia: A Comparative Analysis of Average Shale and Upper Continental Crust Background Values

Hafizou M. Sow [1,*], Quanrong Wang [1,2,*], Mohamed Hussein Yousif [1], Fred B. Wright [3], Kaixu Chen [4], Chong Chen [4] and Abara A. Biabak Indrick [1]

[1] School of Environmental Studies, China University of Geosciences, Wuhan 430074, China
[2] MOE Key Laboratory of Groundwater Quality and Health, China University of Geosciences, Wuhan 430078, China
[3] School of Marine Science and Technology, China University of Geosciences, Wuhan 430074, China
[4] Wuhan Center, China Geological Survey, Wuhan 430205, China
* Correspondence: hafizmsow@cug.edu.cn (H.M.S.); wangqr@cug.edu.cn (Q.W.)

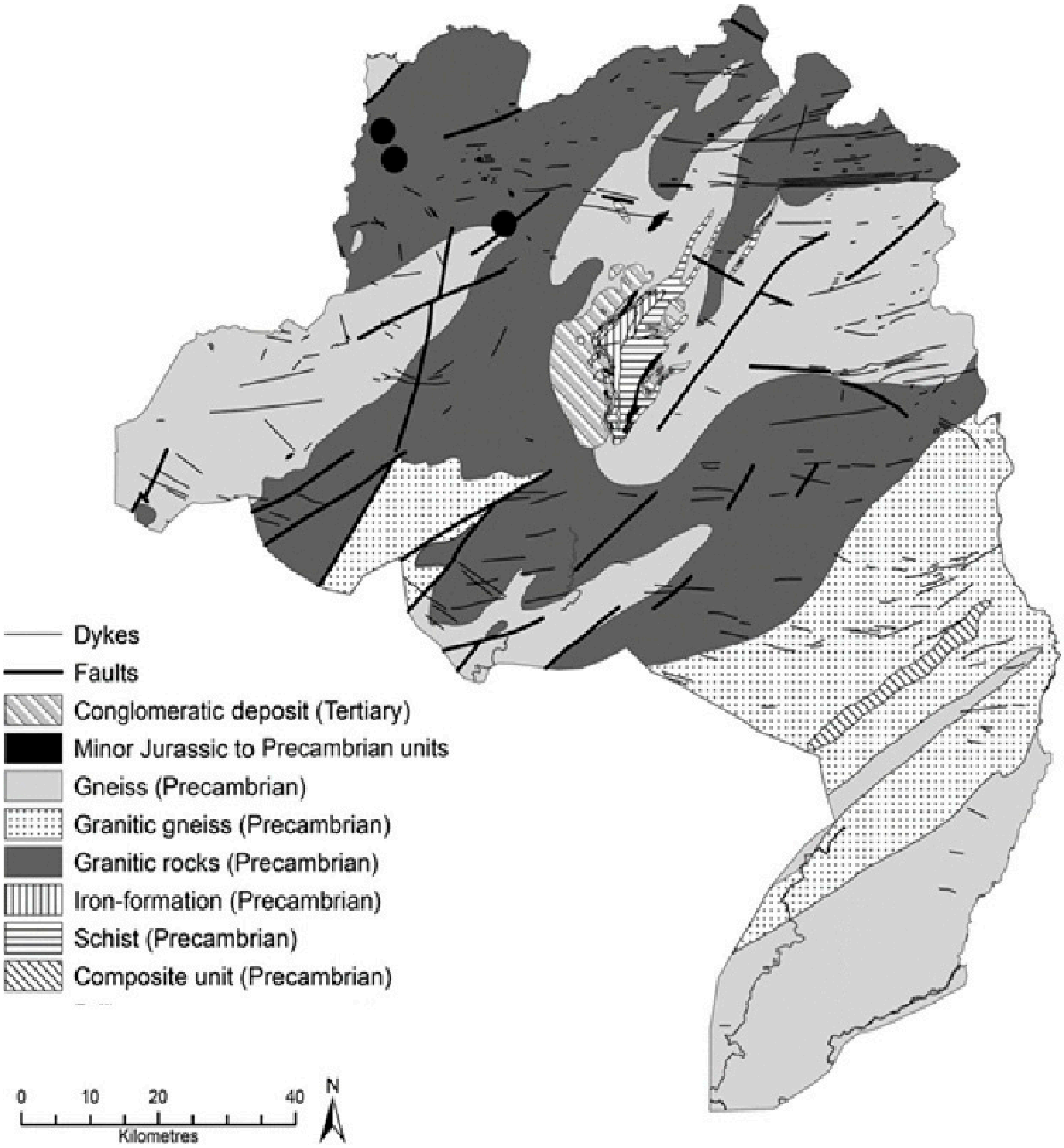

Figure S1. Geological map of Lofa County modified from (1).

Supplementary Information (SI-1)

1. Laboratory Sample Preparation

Samples were oven-dried at a temperature of 45 °C. After drying, 80g of the sample was weighed and ground in a pollution-free agate ball mill to -200 mesh. A total of 10 grams of the sample was used for the analysis of As and Hg by AFS, and the remaining sub-sample was dried at 105°C for two hours for the determination of other elements. Quality control was ensured by analyzing the detection limit and percent of report, and the accuracy and precision control of the analysis methods.

1.1. Sample laboratory analysis and quality control.

1.2. Laboratory analysis and quality control.

All samples were analyzed by the laboratory of the Central South Mineral Resources Supervision and Testing Center, People's Republic of China. Laboratory work included sample analysis, including the determination of elemental analysis methods, sample preparation, and quality control of analysis results, among which the methods and processes of sample preparation and quality control of analysis results are the same.

1.3. Sample preparation for different analytical methods.

1.3.1. Sample preparation for inductively coupled plasma mass spectrometry (ICP-MS) analysis included the decomposition of 0.1000 g of the sample by HF, HCl, HNO3, and HClO4. The solution was fixed by 1% HCl to a volume of 100 mL.

1.3.2. X-ray fluorescence spectrometry (XRF) was used to analyze Mn. Before analysis, 4 g of each sample was compressed into a powder pellet.

1.3.3. Sample preparation for atomic fluorescence spectrometry (AFS) analysis included the decomposition of 0.2500 g of the sample by aqua regia. The solution was extracted by 50% HCl and diluted with 10% hydrochloric solution, reduced by thiourea–ascorbic acid. As and Hg were respectively determined by a high-intensity hollow-cathode lamp as the excitation source and potassium borohydride as the hydride generator.

2. Detection limit and percent of reportability of each element.

The detection limits of the analysis of each element during the analysis varied, as presented in Table S1.

Table S1. Element detection limits.

S.N.	Element	Analytical Method	Detection Limits (ppm)	$\%R = \frac{\#\, of\ detected\ samples}{Total\ samples} * 100$
1	As	AFS	0.03	100%
2	Cd	ICP-MS	0.001	100%
3	Cr	ICP-MS	0.30	99.68%
4	Cu	ICP-MS	0.02	99.36%
5	Hg	AFS	0.0005	100%
6	Mn	XRF	5	100%
7	Ni	ICP-MS	0.05	99.36%
8	Pb	ICP-MS	0.05	100%
9	Zn	ICP-MS	0.025	100%

3. Laboratory Quality Control

Accuracy and Precision Control

Accuracy was controlled by using national primary standard reference materials (SRMs). Three GBW stream sediment SRMs (GBW07361, GBW07304a, and GBW07309) were inserted into the analyzed samples and were analyzed under the same conditions as the routine samples 14 times. The certified values, determined values, and recovery rates of each SRM are presented in Table S2.

The accuracy and precision of the analysis were controlled by inserting coded samples of the national standard into each batch of samples. Four coded national-grade standard substances (including high, medium, and low contents) were inserted into every 100 samples for sample analysis under the same conditions**.** Similarly, precision was controlled by using SRMs. Four stream sediment SRMs were inserted in each batch of 50 samples and simultaneously analyzed.

Table S2. Certified values (mg/kg), determined values (mg/kg), and recovery rate (%) of SRMs.

		Cu	Pb	Zn	Cd	Cr	Ni	As	Hg	Mn
GBW07309	Certified	32	23	78	0.26	85	32	8.4	0.083	620
	Determined	34.2	24.31	82.9	0.25	95.71	33.76	8.98	0.091	623.89
	Mean	33.10	23.65	80.45	0.255	90.35	32.88	8.69	0.087	621.94
	Std. Dev.	1.555	0.926	3.464	0.007	7.573	1.244	0.410	0.005	2.750
	Recovery (%)	106.88	105.70	106.28	96.15	112.60	105.50	106.90	109.64	100.63
GBW07304a	Certified	33	68	139	0.9	70	28	21	0.078	1010
	Determined	32.34	70.51	137.4	0.97	67.8	33.1	22.58	0.083	1003.88
	Mean	32.67	69.75	138.20	0.935	68.90	30.55	21.79	0.0805	1006.94
	Std. Dev.	0.494	1.774	1.414	0.049	1.555	3.606	1.060	0.003	4.384
	Recovery (%)	98.00	103.69	98.85	107.78	96.86	118.21	107.52	106.41	99.39
GBW07361	Certified	3.9	22	19	0.095	8.4	4.7	4.4	0.016	0.122
	Determined	4.1	22.91	18.48	0.098	8.33	4.73	4.28	0.0168	0.1201
	Mean	4.00	22.455	18.74	0.0965	8.365	4.715	4.34	0.0164	0.121
	Std. Dev.	0.141	0.643	0.367	0.002	0.049	0.021	0.084	0.0005	0.0013
	Recovery (%)	105.13	104.14	97.26	103.16	99.17	100.64	97.27	105.00	98.44

Table S3. Heavy metal contamination factors using both average shale and UCC background values.

	Cu		Pb		Zn		Cr		Ni		Cd		As		Hg	
	Shale	UCC	Shale	UCC	Shale	UCC	Shale	UCC	Shale	UCC	Shale	UCC	Shale	UCC	Shale	UCC*
LKD-1	0.35	0.62	0.33	0.33	0.04	0.06	0.07	0.19	0.06	0.21	0.13	0.41	0.15	1.34	0.04	0.30
LKD-2	0.53	0.96	0.26	0.26	0.11	0.14	0.24	0.62	0.04	0.15	0.11	0.35	0.13	1.11	0.04	0.25
LKD-3	0.28	0.50	0.17	0.17	0.02	0.03	0.29	0.74	0.05	0.16	0.10	0.31	0.13	1.12	0.04	0.27
LKD-4	0.01	0.02	0.70	0.70	0.03	0.04	0.02	0.05	0.00	0.00	0.13	0.41	0.05	0.39	0.04	0.25
LKD-5	0.66	1.19	0.22	0.22	0.14	0.18	0.30	0.76	0.04	0.13	0.12	0.38	0.12	1.03	0.04	0.29
LKD-6	0.01	0.02	0.09	0.09	0.02	0.03	0.06	0.15	0.00	0.00	0.14	0.43	0.08	0.68	0.04	0.25
LKD-7	0.01	0.02	0.12	0.12	0.02	0.02	0.02	0.05	0.01	0.03	0.11	0.34	0.08	0.72	0.03	0.21
LKD-8	0.02	0.04	0.57	0.57	0.06	0.09	0.02	0.05	0.00	0.00	0.12	0.38	0.03	0.30	0.03	0.18
LKD-9	0.48	0.86	0.06	0.06	0.07	0.09	0.14	0.35	0.06	0.19	0.11	0.34	0.19	1.62	0.02	0.18
LKD-10	0.01	0.02	0.09	0.09	0.01	0.02	0.02	0.04	0.01	0.03	0.11	0.35	0.10	0.83	0.04	0.25
LKD-11	0.68	1.23	0.27	0.27	0.15	0.20	0.24	0.61	0.05	0.17	0.12	0.38	0.11	0.97	0.04	0.29
LKD-12	0.64	1.14	1.05	1.05	0.15	0.20	0.20	0.51	0.04	0.14	0.12	0.37	0.10	0.89	0.04	0.27
LKD-13	0.25	0.45	0.12	0.12	0.01	0.02	0.11	0.27	0.04	0.15	0.14	0.43	0.15	1.28	0.04	0.27
LKD-14	0.18	0.32	0.08	0.08	0.02	0.02	0.10	0.25	0.03	0.12	0.12	0.36	0.10	0.90	0.04	0.25
LKD-15	0.03	0.05	0.23	0.23	0.01	0.01	0.02	0.06	0.00	0.00	0.14	0.42	0.05	0.39	0.04	0.27
LKD-16	0.00	0.00	0.16	0.16	0.00	0.00	0.03	0.09	0.00	0.00	0.14	0.43	0.08	0.67	0.04	0.30
LKD-17	0.03	0.06	0.10	0.10	0.02	0.02	0.06	0.16	0.01	0.05	0.13	0.41	0.05	0.47	0.04	0.27
LKD-18	0.02	0.04	0.17	0.17	0.03	0.05	0.09	0.23	0.03	0.09	0.15	0.46	0.07	0.62	0.04	0.25

Continuation of Table S3. Heavy metal contamination factors using both average shale and UCC background values.

	Cu		Pb		Zn		Cr		Ni		Cd		As		Hg	
	Shale	UCC	Shale	UCC	Shale	UCC	Shale	UCC	Shale	UCC	Shale	UCC	Shale	UCC	Shale	UCC*
LKD-19	0.02	0.03	0.16	0.16	0.01	0.02	0.03	0.08	0.00	0.00	0.16	0.48	0.08	0.66	0.05	0.32
LKD-20	0.02	0.03	0.07	0.07	0.01	0.01	0.02	0.05	0.00	0.00	0.14	0.42	0.09	0.80	0.04	0.27
LKD-21	0.03	0.06	0.18	0.18	0.05	0.06	0.07	0.18	0.02	0.07	0.15	0.45	0.08	0.72	0.05	0.32
LKD-22	0.01	0.02	0.04	0.04	0.00	0.01	0.05	0.12	0.00	0.02	0.15	0.45	0.09	0.79	0.04	0.30
LKD-23	0.26	0.47	0.03	0.03	0.02	0.02	0.33	0.86	0.07	0.23	0.11	0.35	0.17	1.51	0.03	0.23
LKD-24	0.23	0.41	0.19	0.19	0.04	0.05	0.35	0.89	0.06	0.20	0.16	0.48	0.13	1.11	0.03	0.23
LKD-25	0.64	1.16	0.17	0.17	0.13	0.17	0.17	0.45	0.05	0.17	0.13	0.41	0.12	1.08	0.04	0.25
LKD-26	0.57	1.02	0.55	0.55	0.12	0.15	0.08	0.19	0.05	0.17	0.15	0.45	0.09	0.79	0.04	0.25
LKD-27	1.01	1.82	0.21	0.21	0.25	0.33	0.30	0.77	0.07	0.23	0.14	0.42	0.15	1.31	0.06	0.39
LKD-28	0.74	1.33	0.01	0.01	0.10	0.14	0.44	1.13	0.10	0.35	0.13	0.40	0.23	1.97	0.04	0.25
LKD-29	0.46	0.84	0.11	0.11	0.06	0.08	0.95	2.45	0.07	0.25	0.12	0.37	0.22	1.92	0.05	0.38
LKD-30	0.37	0.66	0.12	0.12	0.02	0.02	0.12	0.32	0.08	0.27	0.13	0.40	0.16	1.39	0.03	0.21
LKD-31	0.26	0.48	0.23	0.23	0.06	0.08	0.31	0.79	0.11	0.38	0.15	0.45	0.11	0.95	0.03	0.23
LKD-32	0.29	0.52	0.96	0.96	0.03	0.03	0.30	0.77	0.06	0.20	0.16	0.48	0.12	1.07	0.03	0.23
LKD-33	0.37	0.67	0.25	0.25	0.02	0.03	0.40	1.03	0.09	0.31	0.17	0.52	0.12	1.08	0.03	0.21
LKD-34	0.28	0.50	5.40	5.40	0.02	0.03	0.46	1.19	0.11	0.39	0.13	0.40	0.14	1.19	0.03	0.21
LKD-35	0.13	0.24	0.18	0.18	0.01	0.02	0.13	0.33	0.05	0.18	0.11	0.34	0.11	0.93	0.03	0.20
LKD-36	0.24	0.44	0.24	0.24	0.06	0.08	0.17	0.44	0.11	0.38	0.13	0.39	0.13	1.09	0.04	0.25
LKD-37	0.34	0.61	0.23	0.23	0.06	0.09	0.77	1.98	0.08	0.27	0.16	0.48	0.17	1.49	0.05	0.32

Continuation of Table S3. Heavy metals contamination factor using both average shale and UCC background values.

	Cu		Pb		Zn		Cr		Ni		Cd		As		Hg	
	Shale	UCC	Shale	UCC	Shale	UCC	Shale	UCC	Shale	UCC	Shale	UCC	Shale	UCC	Shale	UCC*
LKD-38	0.25	0.46	0.19	0.19	0.02	0.03	0.26	0.66	0.08	0.29	0.16	0.50	0.14	1.23	0.03	0.23
LKD-39	0.50	0.91	0.10	0.10	0.02	0.02	0.10	0.27	0.10	0.33	0.14	0.43	0.19	1.68	0.03	0.23
LKD-40	0.40	0.72	0.11	0.11	0.05	0.07	0.72	1.84	0.09	0.31	0.13	0.41	0.16	1.36	0.04	0.27
LKD-41	0.26	0.47	0.20	0.20	0.05	0.07	0.26	0.68	0.06	0.20	0.12	0.36	0.10	0.83	0.03	0.21
LKD-42	0.22	0.40	0.24	0.24	0.05	0.07	0.22	0.56	0.05	0.17	0.13	0.41	0.15	1.31	0.04	0.25
LKD-43	0.02	0.03	0.04	0.04	0.09	0.13	0.11	0.28	0.01	0.02	0.10	0.31	0.04	0.31	0.03	0.20
LKD-44	0.04	0.07	0.10	0.10	0.03	0.04	0.07	0.19	0.02	0.07	0.16	0.48	0.07	0.57	0.04	0.25
LKD-45	0.03	0.05	0.03	0.03	0.03	0.03	0.04	0.11	0.01	0.03	0.11	0.34	0.05	0.43	0.04	0.27
LKD-46	0.01	0.02	0.12	0.12	0.04	0.05	0.03	0.09	0.01	0.04	0.14	0.42	0.05	0.41	0.03	0.20
LKD-47	0.20	0.37	0.12	0.12	0.07	0.10	0.11	0.28	0.06	0.21	0.13	0.39	0.12	1.04	0.04	0.29
LKD-48	0.30	0.54	0.21	0.21	0.08	0.10	0.49	1.27	0.08	0.26	0.09	0.29	0.14	1.20	0.04	0.30
LKD-49	0.21	0.37	1.02	1.02	0.03	0.04	0.29	0.74	0.06	0.19	0.11	0.33	0.15	1.33	0.03	0.20
LKD-50	0.30	0.54	3.07	3.07	0.03	0.04	0.35	0.91	0.06	0.21	0.15	0.45	0.16	1.38	0.03	0.18
LKD-51	0.27	0.48	0.12	0.12	0.01	0.02	0.23	0.59	0.07	0.24	0.13	0.40	0.15	1.31	0.03	0.21
LKD-52	0.40	0.72	0.04	0.04	0.02	0.03	0.09	0.23	0.08	0.27	0.10	0.30	0.16	1.37	0.03	0.23
LKD-53	0.20	0.36	0.42	0.42	0.04	0.05	0.23	0.59	0.04	0.15	0.15	0.45	0.09	0.78	0.03	0.23
LKD-54	0.28	0.50	0.11	0.11	0.06	0.07	0.28	0.73	0.06	0.21	0.11	0.35	0.07	0.62	0.04	0.27
LKD-55	0.28	0.50	0.12	0.12	0.05	0.07	0.31	0.79	0.05	0.18	0.13	0.41	0.07	0.61	0.03	0.21
LKD-56	0.18	0.32	0.23	0.23	0.03	0.05	0.24	0.63	0.04	0.14	0.14	0.44	0.06	0.55	0.03	0.20

Continuation of Table S3. Heavy metal contamination factors using both average shale and UCC background values.

	Cu		Pb		Zn		Cr		Ni		Cd		As		Hg	
	Shale	UCC	Shale	UCC	Shale	UCC	Shale	UCC	Shale	UCC	Shale	UCC	Shale	UCC	Shale	UCC*
LKD-57	0.01	0.03	0.09	0.09	0.04	0.05	0.04	0.10	0.01	0.03	0.14	0.43	0.08	0.67	0.03	0.20
LKD-58	0.04	0.07	0.09	0.09	0.04	0.05	0.06	0.16	0.02	0.08	0.12	0.36	0.01	0.13	0.03	0.23
LKD-59	0.02	0.03	0.10	0.10	0.06	0.08	0.02	0.06	0.01	0.03	0.13	0.40	0.03	0.24	0.03	0.23
LKD-60	0.03	0.05	0.10	0.10	0.05	0.07	0.03	0.08	0.01	0.04	0.09	0.29	0.01	0.06	0.03	0.21
LKD-61	0.49	0.88	0.05	0.05	0.03	0.04	0.53	1.37	0.10	0.34	0.17	0.52	0.14	1.22	0.03	0.18
LKD-62	0.48	0.87	0.07	0.07	0.06	0.08	0.12	0.30	0.07	0.23	0.14	0.43	0.12	1.03	0.03	0.18
LKD-63	0.54	0.97	0.23	0.23	0.06	0.08	0.42	1.07	0.10	0.34	0.13	0.39	0.19	1.63	0.03	0.23
LKD-64	0.25	0.46	0.50	0.50	0.03	0.04	0.33	0.85	0.06	0.19	0.13	0.39	0.15	1.27	0.04	0.25
LKD-65	0.52	0.94	0.16	0.16	0.09	0.11	0.35	0.90	0.06	0.19	0.13	0.41	0.11	0.95	0.07	0.50
LKD-66	0.50	0.89	0.09	0.09	0.01	0.01	0.11	0.29	0.10	0.33	0.13	0.41	0.16	1.39	0.03	0.21
LKD-67	0.38	0.68	0.10	0.10	0.01	0.02	0.58	1.50	0.08	0.26	0.13	0.40	0.13	1.09	0.03	0.18
LKD-68	0.32	0.58	0.02	0.02	0.02	0.03	0.31	0.80	0.07	0.24	0.15	0.45	0.14	1.19	0.03	0.18
LKD-69	0.03	0.05	0.05	0.05	0.02	0.02	0.35	0.91	0.07	0.23	0.14	0.42	0.03	0.27	0.04	0.25
LKD-70	0.17	0.30	0.03	0.03	0.03	0.04	0.15	0.39	0.04	0.14	0.11	0.33	0.11	0.98	0.03	0.20
LKD-71	0.04	0.08	0.06	0.06	0.02	0.02	0.35	0.91	0.06	0.21	0.11	0.34	0.03	0.27	0.04	0.25
LKD-72	0.02	0.03	0.03	0.03	0.01	0.02	0.03	0.08	0.01	0.03	0.11	0.33	0.01	0.10	0.03	0.18
LKD-73	0.04	0.08	0.23	0.23	0.08	0.11	0.04	0.10	0.03	0.12	0.12	0.36	0.13	1.13	0.06	0.41
LKD-74	0.03	0.05	0.14	0.14	0.03	0.04	0.05	0.14	0.01	0.05	0.13	0.41	0.03	0.25	0.04	0.25
LKD-75	0.02	0.04	0.06	0.06	0.02	0.03	0.06	0.16	0.01	0.05	0.11	0.35	0.04	0.37	0.03	0.21

Continuation of Table S3. Heavy metal contamination factors using both average shale and UCC background values.

	Cu		Pb		Zn		Cr		Ni		Cd		As		Hg	
	Shale	UCC	Shale	UCC	Shale	UCC	Shale	UCC	Shale	UCC	Shale	UCC	Shale	UCC	Shale	UCC*
LKD-76	0.01	0.02	0.06	0.06	0.01	0.02	0.03	0.08	0.01	0.02	0.10	0.30	0.03	0.24	0.03	0.21
LKD-77	0.44	0.79	0.21	0.21	0.09	0.12	0.23	0.59	0.06	0.19	0.13	0.39	0.08	0.67	0.03	0.20
LKD-78	0.65	1.16	0.30	0.30	0.15	0.21	0.10	0.26	0.06	0.19	0.14	0.42	0.09	0.77	0.04	0.25
LKD-79	0.19	0.34	0.34	0.34	0.02	0.03	0.13	0.33	0.05	0.16	0.11	0.33	0.08	0.73	0.03	0.20
LKD-80	0.16	0.29	0.16	0.16	0.01	0.02	0.20	0.51	0.03	0.09	0.16	0.50	0.07	0.57	0.03	0.18
LKD-81	0.15	0.27	0.03	0.03	0.02	0.03	0.11	0.29	0.03	0.11	0.11	0.33	0.09	0.75	0.02	0.18
LKD-82	0.32	0.58	0.00	0.00	0.02	0.02	0.07	0.19	0.06	0.20	0.12	0.37	0.07	0.64	0.03	0.20
LKD-83	0.21	0.39	0.07	0.07	0.03	0.04	0.35	0.90	0.05	0.18	0.10	0.32	0.06	0.54	0.03	0.21
LKD-84	0.27	0.49	0.05	0.05	0.03	0.05	0.26	0.67	0.05	0.19	0.09	0.28	0.08	0.67	0.03	0.20
LKD-85	0.15	0.27	0.27	0.27	0.01	0.01	0.16	0.41	0.03	0.12	0.10	0.32	0.04	0.39	0.02	0.18
LKD-86	0.39	0.70	0.11	0.11	0.15	0.20	0.35	0.89	0.07	0.24	0.12	0.38	0.14	1.19	0.03	0.20
LKD-87	0.04	0.08	0.13	0.13	0.03	0.05	0.09	0.22	0.04	0.13	0.11	0.33	0.01	0.07	0.04	0.27
LKD-88	0.07	0.13	0.18	0.18	0.08	0.11	0.18	0.46	0.04	0.13	0.13	0.40	0.01	0.10	0.03	0.21
LKD-89	0.00	0.01	0.09	0.09	0.05	0.07	0.03	0.09	0.00	0.02	0.08	0.23	0.03	0.24	0.03	0.18
LKD-90	0.04	0.07	0.05	0.05	0.03	0.05	0.05	0.12	0.00	0.00	0.12	0.38	0.00	0.02	0.02	0.16
LKD-91	0.33	0.59	0.19	0.19	0.05	0.07	0.22	0.57	0.79	2.69	0.13	0.41	0.08	0.65	0.03	0.23
LKD-92	0.26	0.47	0.18	0.18	0.02	0.02	0.08	0.20	0.73	2.49	0.13	0.41	0.09	0.79	0.03	0.21
LKD-93	0.18	0.32	0.09	0.09	0.02	0.03	0.11	0.28	0.78	2.66	0.12	0.37	0.07	0.63	0.03	0.21
LKD-94	0.13	0.23	0.11	0.11	0.02	0.03	0.15	0.38	1.36	4.63	0.11	0.34	0.08	0.70	0.03	0.20

Continuation of Table S3. Heavy metal contamination factors using both average shale and UCC background values.

	Cu		Pb		Zn		Cr		Ni		Cd		As		Hg	
	Shale	UCC	Shale	UCC	Shale	UCC	Shale	UCC	Shale	UCC	Shale	UCC	Shale	UCC	Shale	UCC*
LKD-95	0.18	0.33	0.09	0.09	0.03	0.04	0.11	0.28	0.77	2.61	0.12	0.38	0.05	0.45	0.03	0.21
LKD-96	0.42	0.76	0.07	0.07	0.02	0.03	0.10	0.26	0.90	3.07	0.10	0.32	0.11	0.95	0.03	0.18
LKD-97	0.21	0.38	0.08	0.08	0.00	0.00	0.34	0.89	0.05	0.18	0.03	0.08	0.08	0.71	0.03	0.23
LKD-98	0.16	0.28	0.10	0.10	0.01	0.01	0.17	0.45	0.02	0.08	0.01	0.04	0.10	0.90	0.04	0.29
LKD-99	0.20	0.36	0.08	0.08	0.00	0.00	0.32	0.82	0.03	0.10	0.01	0.02	0.09	0.78	0.03	0.21
LKD-100	0.19	0.33	0.15	0.15	0.00	0.00	0.18	0.45	0.03	0.10	0.07	0.20	0.07	0.61	0.04	0.25
LKD-101	0.01	0.02	0.13	0.13	0.00	0.00	0.03	0.09	0.00	0.00	0.01	0.02	0.05	0.43	0.03	0.23
LKD-102	0.01	0.02	0.46	0.46	0.01	0.01	0.03	0.09	0.00	0.00	0.01	0.02	0.02	0.13	0.04	0.29
LKD-103	0.05	0.08	0.10	0.10	0.00	0.00	0.03	0.08	0.00	0.00	0.01	0.02	0.01	0.12	0.04	0.27
LKD-104	0.16	0.29	0.31	0.31	0.00	0.00	0.14	0.35	0.01	0.05	0.05	0.15	0.06	0.53	0.04	0.25
LKD-105	0.27	0.48	0.37	0.37	0.01	0.02	0.07	0.18	0.04	0.13	0.05	0.14	0.10	0.91	0.04	0.29
LKD-106	0.21	0.38	0.10	0.10	0.00	0.00	0.11	0.28	0.22	0.74	0.10	0.32	0.09	0.81	0.03	0.23
LKD-107	0.10	0.18	0.12	0.12	0.00	0.00	0.16	0.41	0.04	0.14	0.02	0.07	0.09	0.81	0.04	0.25
LKD-108	0.21	0.38	0.08	0.08	0.02	0.02	0.17	0.44	0.10	0.34	0.01	0.02	0.10	0.85	0.03	0.23
LKD-109	0.26	0.48	0.09	0.09	0.02	0.02	0.05	0.12	0.06	0.21	0.06	0.18	0.11	0.94	0.04	0.25
LKD-110	0.25	0.45	0.13	0.13	0.08	0.10	0.33	0.85	0.05	0.17	0.08	0.23	0.09	0.76	0.03	0.20
LKD-111	0.15	0.27	0.18	0.18	0.00	0.00	0.15	0.38	0.03	0.10	0.02	0.05	0.06	0.55	0.04	0.25
LKD-112	0.74	1.32	0.16	0.16	0.15	0.20	0.20	0.52	0.03	0.09	0.01	0.02	0.11	0.91	0.03	0.21
LKD-113	0.20	0.36	0.15	0.15	0.00	0.00	0.18	0.46	0.03	0.10	0.07	0.21	0.09	0.81	0.03	0.21

Continuation of Table S3. Heavy metal contamination factors using both average shale and UCC background values.

	Cu		Pb		Zn		Cr		Ni		Cd		As		Hg	
	Shale	UCC	Shale	UCC	Shale	UCC	Shale	UCC	Shale	UCC	Shale	UCC	Shale	UCC	Shale	UCC*
LKD-114	0.14	0.26	0.10	0.10	0.00	0.01	0.14	0.37	0.03	0.09	0.02	0.07	0.05	0.40	0.04	0.25
LKD-115	0.02	0.03	0.46	0.46	0.02	0.02	0.05	0.13	0.10	0.35	0.01	0.03	0.02	0.18	0.04	0.27
LKD-116	0.02	0.03	0.37	0.37	0.00	0.00	0.02	0.06	0.03	0.09	0.08	0.24	0.04	0.36	0.04	0.27
LKD-117	0.02	0.04	0.49	0.49	0.03	0.04	0.05	0.12	0.01	0.03	0.03	0.10	0.01	0.10	0.04	0.25
LKD-118	0.19	0.34	0.32	0.32	0.04	0.05	0.17	0.45	0.04	0.13	0.02	0.06	0.08	0.73	0.04	0.29
LKD-119	0.15	0.26	0.12	0.12	0.00	0.00	0.07	0.18	0.02	0.07	0.07	0.22	0.10	0.84	0.04	0.25
LKD-120	0.22	0.40	0.28	0.28	0.08	0.11	0.29	0.74	0.05	0.19	0.04	0.13	0.14	1.18	0.04	0.29
LKD-121	0.14	0.26	0.10	0.10	0.00	0.00	0.18	0.47	0.05	0.15	0.04	0.13	0.11	0.92	0.04	0.27
LKD-122	0.13	0.24	0.08	0.08	0.00	0.00	0.13	0.33	0.04	0.13	0.10	0.30	0.08	0.72	0.03	0.23
LKD-123	0.29	0.52	0.11	0.11	0.00	0.00	0.05	0.13	0.05	0.17	0.08	0.23	0.13	1.09	0.03	0.20
LKD-124	0.21	0.38	0.09	0.09	0.00	0.00	0.31	0.81	0.03	0.09	0.07	0.20	0.12	1.01	0.04	0.25
LKD-125	0.16	0.29	0.17	0.17	0.00	0.00	0.13	0.34	0.03	0.11	0.09	0.27	0.10	0.87	0.04	0.30
LKD-126	0.19	0.34	0.12	0.12	0.00	0.00	0.22	0.57	0.02	0.08	0.06	0.18	0.14	1.19	0.03	0.21
LKD-127	0.25	0.46	0.08	0.08	0.02	0.02	0.28	0.73	0.04	0.14	0.01	0.03	0.04	0.32	0.03	0.23
LKD-128	0.01	0.02	0.21	0.21	0.00	0.00	0.02	0.05	0.00	0.00	0.04	0.13	0.14	1.22	0.04	0.27
LKD-129	0.02	0.03	0.29	0.29	0.00	0.00	0.01	0.02	0.00	0.00	0.11	0.33	0.06	0.48	0.03	0.21
LKD-130	0.03	0.06	0.47	0.47	0.05	0.06	0.06	0.15	0.03	0.10	0.01	0.02	0.01	0.09	0.03	0.23
LKD-131	0.00	0.00	0.53	0.53	0.04	0.05	0.03	0.08	0.01	0.04	0.01	0.02	0.04	0.36	0.04	0.27
LKD-132	0.20	0.35	0.15	0.15	0.03	0.04	0.21	0.54	0.04	0.13	0.04	0.11	0.10	0.84	0.04	0.25

Continuation of Table S3. Heavy metal contamination factors using both average shale and UCC background values.

	Cu		Pb		Zn		Cr		Ni		Cd		As		Hg	
	Shale	UCC	Shale	UCC	Shale	UCC	Shale	UCC	Shale	UCC	Shale	UCC	Shale	UCC	Shale	UCC*
LKD-133	0.23	0.42	0.16	0.16	0.03	0.04	0.11	0.28	0.05	0.18	0.04	0.11	0.11	0.97	0.03	0.21
LKD-134	0.20	0.36	0.18	0.18	0.02	0.03	0.27	0.68	0.05	0.16	0.01	0.02	0.13	1.11	0.04	0.25
LKD-135	0.24	0.43	0.22	0.22	0.01	0.02	0.26	0.66	0.07	0.25	0.12	0.36	0.12	1.05	0.03	0.20
LKD-136	0.23	0.42	0.24	0.24	0.04	0.05	0.36	0.92	0.08	0.29	0.03	0.10	0.09	0.80	0.04	0.27
LKD-137	0.36	0.64	0.32	0.32	0.06	0.09	0.10	0.25	0.08	0.28	0.02	0.07	0.13	1.17	0.03	0.21
LKD-138	0.22	0.39	0.09	0.09	0.00	0.00	0.35	0.90	0.05	0.16	0.07	0.22	0.11	0.92	0.02	0.17
LKD-139	0.16	0.29	0.14	0.14	0.02	0.03	0.14	0.37	0.03	0.11	0.07	0.22	0.08	0.69	0.03	0.21
LKD-140	0.18	0.32	0.11	0.11	0.03	0.04	0.15	0.39	0.03	0.09	0.03	0.08	0.12	1.05	0.03	0.21
LKD-141	0.18	0.33	0.13	0.13	0.01	0.01	0.13	0.33	0.04	0.12	0.05	0.14	0.17	1.50	0.03	0.20
LKD-142	0.01	0.02	0.23	0.23	0.01	0.02	0.04	0.11	0.00	0.00	0.01	0.02	0.05	0.39	0.03	0.20
LKD-143	0.01	0.03	0.30	0.30	0.05	0.06	0.02	0.05	0.00	0.00	0.06	0.18	0.04	0.33	0.03	0.23
LKD-144	0.00	0.01	0.31	0.31	0.04	0.05	0.00	0.01	0.00	0.00	0.01	0.02	0.06	0.53	0.03	0.20
LKD-145	0.00	0.01	0.38	0.38	0.05	0.06	0.00	0.01	0.00	0.00	0.02	0.05	0.02	0.14	0.03	0.23
LKD-146	0.27	0.49	0.00	0.00	0.00	0.00	0.36	0.93	0.05	0.16	0.01	0.02	0.16	1.38	0.03	0.20
LKD-147	0.11	0.20	0.07	0.07	0.00	0.00	0.01	0.02	0.03	0.09	0.01	0.02	0.07	0.65	0.03	0.21
LKD-148	0.15	0.27	0.10	0.10	0.04	0.06	0.12	0.31	0.05	0.19	0.01	0.02	0.11	0.95	0.04	0.27
LKD-149	0.09	0.16	0.04	0.04	0.02	0.03	0.08	0.21	0.02	0.07	0.02	0.06	0.06	0.50	0.04	0.30
LKD-150	0.13	0.24	0.06	0.06	0.00	0.00	0.16	0.41	0.04	0.13	0.01	0.02	0.07	0.63	0.04	0.25
LKD-151	0.32	0.58	0.00	0.00	0.00	0.00	0.02	0.05	0.06	0.22	0.01	0.02	0.14	1.23	0.04	0.30

Continuation of Table S3. Heavy metal contamination factors using both average shale and UCC background values.

	Cu		Pb		Zn		Cr		Ni		Cd		As		Hg	
	Shale	UCC	Shale	UCC	Shale	UCC	Shale	UCC	Shale	UCC	Shale	UCC	Shale	UCC	Shale	UCC*
LKD-152	0.28	0.50	0.03	0.03	0.00	0.00	0.45	1.17	0.07	0.23	0.01	0.02	0.11	0.99	0.04	0.25
LKD-153	0.33	0.60	0.08	0.08	0.03	0.04	0.34	0.88	0.06	0.20	0.01	0.02	0.16	1.41	0.04	0.27
LKD-154	0.10	0.19	0.01	0.01	0.02	0.03	0.10	0.26	0.01	0.05	0.21	0.63	0.10	0.90	0.03	0.21
LKD-155	0.17	0.30	0.36	0.36	0.03	0.04	0.24	0.61	0.04	0.15	0.01	0.03	0.10	0.89	0.03	0.21
LKD-156	0.00	0.00	0.16	0.16	0.02	0.03	0.00	0.01	0.00	0.00	0.01	0.03	0.02	0.21	0.04	0.27
LKD-157	0.02	0.03	0.26	0.26	0.04	0.06	0.02	0.06	0.01	0.02	0.02	0.07	0.03	0.26	0.03	0.23
LKD-158	0.08	0.15	0.02	0.02	0.10	0.13	0.75	1.92	0.08	0.27	0.01	0.03	0.03	0.23	0.04	0.30
LKD-159	0.09	0.17	0.05	0.05	0.02	0.03	0.09	0.24	0.04	0.13	0.02	0.07	0.07	0.57	0.04	0.30
LKD-160	0.23	0.41	0.19	0.19	0.18	0.24	0.15	0.39	0.16	0.54	0.01	0.02	0.13	1.15	0.15	1.09
LKD-161	0.15	0.27	0.07	0.07	0.10	0.13	0.78	2.02	0.04	0.14	0.01	0.02	0.18	1.57	0.06	0.45
LKD-162	0.16	0.29	0.05	0.05	0.05	0.07	0.15	0.37	0.04	0.13	0.01	0.02	0.09	0.79	0.05	0.32
LKD-163	0.27	0.48	0.00	0.00	0.08	0.11	0.06	0.15	0.06	0.21	0.02	0.05	0.11	0.91	0.04	0.27
LKD-164	0.15	0.27	0.02	0.02	0.06	0.08	0.24	0.62	0.03	0.12	0.01	0.02	0.07	0.63	0.03	0.23
LKD-165	0.50	0.90	0.15	0.15	0.09	0.11	0.53	1.37	0.12	0.42	0.25	0.78	0.22	1.94	0.03	0.18
LKD-166	0.19	0.34	0.03	0.03	0.00	0.00	0.22	0.57	0.05	0.17	0.01	0.02	0.11	0.97	0.03	0.21
LKD-167	0.14	0.25	0.00	0.00	0.00	0.00	0.17	0.44	0.03	0.09	0.01	0.02	0.19	1.61	0.04	0.27
LKD-168	0.22	0.40	0.01	0.01	0.03	0.03	0.27	0.70	0.07	0.24	0.01	0.02	0.10	0.87	0.03	0.23
LKD-169	0.01	0.02	0.05	0.05	0.02	0.03	0.00	0.01	0.01	0.02	0.01	0.02	0.04	0.39	0.04	0.25
LKD-170	0.00	0.01	0.02	0.02	0.03	0.04	0.01	0.02	0.01	0.02	0.01	0.02	0.13	1.14	0.04	0.27

Continuation of Table S3. Heavy metal contamination factors using both average shale and UCC background values.

	Cu		Pb		Zn		Cr		Ni		Cd		As		Hg	
	Shale	UCC	Shale	UCC	Shale	UCC	Shale	UCC	Shale	UCC	Shale	UCC	Shale	UCC	Shale	UCC*
LKD-171	0.14	0.26	0.03	0.03	0.01	0.01	0.18	0.47	0.04	0.12	0.01	0.02	0.13	1.11	0.03	0.23
LKD-172	0.26	0.47	0.10	0.10	0.01	0.01	0.03	0.09	0.05	0.17	0.01	0.02	0.23	1.97	0.06	0.41
LKD-173	0.19	0.35	0.04	0.04	0.01	0.01	0.35	0.89	0.03	0.11	0.02	0.05	0.14	1.19	0.04	0.30
LKD-174	0.13	0.23	0.00	0.00	0.02	0.02	0.06	0.15	0.02	0.08	0.16	0.49	0.17	1.44	0.04	0.29
LKD-175	0.17	0.30	0.00	0.00	0.01	0.02	0.19	0.49	0.03	0.11	0.01	0.02	0.11	0.97	0.03	0.23
LKD-176	0.27	0.48	0.00	0.00	0.02	0.03	0.35	0.90	0.09	0.30	0.01	0.02	0.18	1.55	0.04	0.29
LKD-177	0.19	0.34	0.07	0.07	0.04	0.05	0.20	0.52	0.06	0.21	0.01	0.02	0.16	1.37	0.03	0.21
LKD-178	0.32	0.58	0.00	0.00	0.01	0.01	0.35	0.90	0.07	0.24	0.01	0.02	0.20	1.75	0.04	0.29
LKD-179	0.51	0.92	0.02	0.02	0.10	0.14	0.04	0.10	0.04	0.15	0.01	0.02	0.11	0.91	0.04	0.25
LKD-180	0.16	0.29	0.15	0.15	0.07	0.09	0.24	0.61	0.07	0.24	0.01	0.02	0.13	1.16	0.03	0.23
LKD-181	0.02	0.03	0.19	0.19	0.11	0.15	0.01	0.03	0.00	0.01	0.00	0.01	0.04	0.34	0.04	0.25
LKD-182	0.00	0.00	0.18	0.18	0.02	0.03	0.03	0.06	0.01	0.03	0.15	0.47	0.02	0.16	0.04	0.27
LKD-183	0.02	0.04	0.16	0.16	0.02	0.03	0.20	0.52	0.03	0.11	0.01	0.02	0.06	0.50	0.05	0.36
LKD-184	0.01	0.01	0.14	0.14	0.11	0.14	0.02	0.06	0.02	0.07	0.01	0.02	0.04	0.35	0.05	0.32
LKD-185	0.16	0.28	0.47	0.47	0.09	0.12	0.16	0.42	0.03	0.11	0.01	0.02	0.11	0.93	0.03	0.21
LKD-186	0.32	0.58	0.04	0.04	0.04	0.05	0.32	0.83	0.06	0.22	0.01	0.03	0.28	2.42	0.07	0.46
LKD-187	0.12	0.22	0.00	0.00	0.02	0.02	0.07	0.17	0.03	0.11	0.18	0.54	0.15	1.27	0.04	0.25
LKD-188	0.12	0.22	0.00	0.00	0.07	0.10	0.17	0.44	0.04	0.12	0.01	0.03	0.09	0.76	0.03	0.21
LKD-189	0.28	0.50	0.08	0.08	0.05	0.06	0.43	1.10	0.08	0.26	0.12	0.36	0.12	1.05	0.02	0.17

Continuation of Table S3. Heavy metal contamination factors using both average shale and UCC background values.

	Cu		Pb		Zn		Cr		Ni		Cd		As		Hg	
	Shale	UCC	Shale	UCC	Shale	UCC	Shale	UCC	Shale	UCC	Shale	UCC	Shale	UCC	Shale	UCC*
LKD-190	0.21	0.38	0.09	0.09	0.05	0.07	0.15	0.40	0.02	0.08	0.07	0.22	0.11	0.98	0.03	0.21
LKD-191	0.26	0.46	0.12	0.12	0.06	0.08	0.26	0.67	0.07	0.24	0.06	0.18	0.16	1.37	0.02	0.16
LKD-192	0.35	0.62	0.13	0.13	0.07	0.09	0.39	0.99	0.07	0.25	0.06	0.18	0.17	1.43	0.03	0.18
LKD-193	0.28	0.51	0.13	0.13	0.08	0.11	0.21	0.55	0.13	0.45	0.08	0.26	0.12	1.05	0.03	0.21
LKD-194	0.23	0.41	0.13	0.13	0.06	0.08	0.35	0.91	0.09	0.29	0.05	0.15	0.14	1.21	0.03	0.21
LKD-195	0.03	0.05	0.19	0.19	0.06	0.08	0.02	0.06	0.00	0.00	0.05	0.16	0.02	0.20	0.03	0.20
LKD-196	0.02	0.04	0.18	0.18	0.06	0.08	0.02	0.06	0.00	0.00	0.08	0.23	0.02	0.18	0.03	0.21
LKD-197	0.02	0.04	0.40	0.40	0.07	0.09	0.07	0.18	0.02	0.07	0.07	0.22	0.01	0.11	0.03	0.18
LKD-198	0.03	0.05	0.35	0.35	0.07	0.09	0.11	0.29	0.02	0.08	0.08	0.24	0.02	0.16	0.03	0.18
LKD-199	0.25	0.45	0.15	0.15	0.08	0.10	0.17	0.44	0.01	0.03	0.15	0.45	0.05	0.42	0.03	0.20
LKD-200	0.37	0.66	0.12	0.12	0.06	0.08	0.10	0.26	0.01	0.02	0.07	0.21	0.11	0.95	0.03	0.20
LKD-201	0.18	0.33	0.12	0.12	0.07	0.10	0.27	0.69	0.09	0.31	0.08	0.23	0.07	0.64	0.03	0.21
LKD-202	0.21	0.39	0.10	0.10	0.06	0.08	0.22	0.58	0.05	0.16	0.09	0.28	0.09	0.80	0.03	0.18
LKD-203	0.26	0.47	0.13	0.13	0.06	0.09	0.46	1.17	0.08	0.26	0.06	0.17	0.12	1.08	0.03	0.20
LKD-204	0.32	0.57	0.13	0.13	0.08	0.11	0.29	0.74	0.08	0.29	0.10	0.32	0.11	0.99	0.03	0.18
LKD-205	0.19	0.34	0.12	0.12	0.07	0.09	0.14	0.35	0.02	0.08	0.08	0.23	0.13	1.09	0.03	0.21
LKD-206	0.18	0.33	0.14	0.14	0.07	0.10	0.06	0.15	0.00	0.00	0.07	0.21	0.07	0.57	0.02	0.16
LKD-207	0.07	0.13	0.15	0.15	0.04	0.06	0.06	0.16	0.00	0.00	0.08	0.24	0.06	0.53	0.02	0.17
LKD-208	0.28	0.50	0.16	0.16	0.05	0.07	0.30	0.78	0.03	0.12	0.05	0.15	0.14	1.25	0.03	0.23

Continuation of Table S3. Heavy metals contamination factor using both average shale and UCC background values.

	Cu		Pb		Zn		Cr		Ni		Cd		As		Hg	
	Shale	UCC	Shale	UCC	Shale	UCC	Shale	UCC	Shale	UCC	Shale	UCC	Shale	UCC	Shale	UCC*
LKD-209	0.02	0.04	0.16	0.16	0.05	0.07	0.03	0.09	0.04	0.13	0.06	0.19	0.02	0.17	0.02	0.17
LKD-210	0.04	0.07	2.31	2.31	0.15	0.20	0.26	0.68	0.11	0.36	0.09	0.27	0.02	0.15	0.02	0.17
LKD-211	0.02	0.04	0.15	0.15	0.05	0.06	0.02	0.06	0.05	0.15	0.06	0.17	0.01	0.13	0.02	0.17
LKD-212	0.03	0.05	0.09	0.09	0.05	0.06	0.09	0.23	0.00	0.00	0.07	0.22	0.01	0.07	0.04	0.25
LKD-213	0.19	0.34	0.08	0.08	0.04	0.06	0.36	0.94	0.01	0.04	0.08	0.23	0.09	0.80	0.02	0.17
LKD-214	0.42	0.76	0.14	0.14	0.05	0.07	0.10	0.26	0.08	0.27	0.06	0.18	0.14	1.25	0.02	0.16
LKD-215	0.37	0.66	0.20	0.20	0.15	0.20	1.09	2.81	0.17	0.59	0.10	0.31	0.17	1.50	0.02	0.17
LKD-216	0.22	0.40	0.18	0.18	0.05	0.06	0.19	0.48	0.04	0.13	0.03	0.10	0.08	0.73	0.03	0.20
LKD-217	0.13	0.23	0.24	0.24	0.07	0.09	0.35	0.89	0.05	0.18	0.07	0.20	0.12	1.00	0.02	0.16
LKD-218	0.37	0.66	0.13	0.13	0.08	0.11	0.46	1.17	0.10	0.34	0.07	0.21	0.16	1.38	0.02	0.16
LKD-219	0.26	0.47	0.18	0.18	0.07	0.10	0.32	0.83	0.03	0.10	0.04	0.13	0.12	1.06	0.03	0.21
LKD-220	0.26	0.48	0.17	0.17	0.07	0.09	0.17	0.45	0.02	0.08	0.05	0.16	0.13	1.10	0.04	0.29
LKD-221	0.15	0.27	0.12	0.12	0.04	0.06	0.26	0.67	0.00	0.00	0.05	0.16	0.11	0.97	0.03	0.18
LKD-222	0.32	0.58	0.20	0.20	0.06	0.08	0.41	1.05	0.07	0.23	0.05	0.15	0.16	1.35	0.03	0.21
LKD-223	0.02	0.04	0.12	0.12	0.05	0.06	0.10	0.25	0.03	0.11	0.07	0.20	0.03	0.24	0.02	0.17
LKD-224	0.03	0.05	0.10	0.10	0.05	0.06	0.08	0.21	0.00	0.00	0.06	0.19	0.04	0.34	0.02	0.17
LKD-225	0.03	0.06	0.12	0.12	0.06	0.08	0.11	0.29	0.09	0.32	0.07	0.21	0.01	0.11	0.03	0.18
LKD-236	0.04	0.06	0.19	0.19	0.07	0.09	0.15	0.39	0.09	0.31	0.11	0.34	0.03	0.29	0.03	0.23
LKD-227	0.12	0.22	0.28	0.28	0.07	0.09	0.35	0.90	0.06	0.21	0.09	0.28	0.08	0.72	0.03	0.20

Continuation of Table S3. Heavy metal contamination factors using both average shale and UCC background values.

	Cu		Pb		Zn		Cr		Ni		Cd		As		Hg	
	Shale	UCC	Shale	UCC	Shale	UCC	Shale	UCC	Shale	UCC	Shale	UCC	Shale	UCC	Shale	UCC*
LKD-228	0.36	0.64	0.35	0.35	0.19	0.26	0.84	2.16	0.10	0.35	0.07	0.22	0.13	1.13	0.02	0.14
LKD-229	0.21	0.38	0.15	0.15	0.07	0.10	0.36	0.94	0.01	0.05	0.08	0.23	0.08	0.68	0.03	0.23
LKD-230	0.24	0.42	0.24	0.24	0.11	0.14	0.28	0.72	0.07	0.23	0.08	0.23	0.08	0.67	0.04	0.25
LKD-231	0.23	0.41	1.02	1.02	0.04	0.05	0.24	0.63	0.06	0.20	0.06	0.17	0.14	1.18	0.02	0.14
LKD-232	0.18	0.32	0.14	0.14	0.05	0.06	0.17	0.43	0.05	0.17	0.10	0.31	0.11	0.93	0.02	0.14
LKD-233	0.25	0.45	0.27	0.27	0.06	0.08	0.14	0.36	0.06	0.19	0.08	0.26	0.08	0.70	0.02	0.13
LKD-234	0.18	0.33	0.11	0.11	0.05	0.07	0.36	0.91	0.05	0.18	0.06	0.18	0.14	1.22	0.02	0.17
LKD-235	0.27	0.49	0.11	0.11	0.06	0.07	0.25	0.64	0.09	0.31	0.08	0.26	0.08	0.67	0.02	0.17
LKD-236	0.03	0.05	0.25	0.25	0.06	0.08	0.08	0.20	0.00	0.00	0.08	0.23	0.02	0.21	0.02	0.15
LKD-237	0.02	0.04	0.13	0.13	0.06	0.07	0.10	0.25	0.03	0.11	0.08	0.23	0.01	0.12	0.03	0.21
LKD-238	0.02	0.04	0.17	0.17	0.05	0.07	0.10	0.25	0.03	0.10	0.10	0.30	0.05	0.43	0.04	0.25
LKD-239	0.04	0.06	0.26	0.26	0.07	0.10	0.04	0.10	0.00	0.00	0.07	0.22	0.02	0.18	0.03	0.18
LKD-240	0.32	0.58	0.55	0.55	0.08	0.11	0.12	0.32	0.20	0.69	0.09	0.28	0.11	0.93	0.03	0.18
LKD-241	0.28	0.51	0.15	0.15	0.05	0.06	0.47	1.20	0.04	0.14	0.07	0.22	0.16	1.37	0.02	0.16
LKD-242	0.20	0.37	0.16	0.16	0.06	0.08	0.33	0.85	0.07	0.25	0.06	0.19	0.09	0.77	0.04	0.27
LKD-243	0.24	0.42	0.12	0.12	0.07	0.09	0.37	0.94	0.09	0.31	0.10	0.32	0.12	1.01	0.03	0.18
LKD-244	0.34	0.60	0.32	0.32	0.10	0.14	0.52	1.33	0.24	0.82	0.08	0.23	0.15	1.27	0.04	0.25
LKD-245	0.30	0.54	0.27	0.27	0.05	0.07	0.21	0.54	0.06	0.21	0.08	0.24	0.14	1.17	0.03	0.23
LKD-246	0.24	0.43	0.22	0.22	0.06	0.08	0.26	0.67	0.07	0.24	0.08	0.24	0.10	0.88	0.04	0.25

Continuation of Table S3. Heavy metal contamination factors using both average shale and UCC background values.

	Cu		Pb		Zn		Cr		Ni		Cd		As		Hg	
	Shale	UCC	Shale	UCC	Shale	UCC	Shale	UCC	Shale	UCC	Shale	UCC	Shale	UCC	Shale	UCC*
LKD-247	0.20	0.36	0.13	0.13	0.05	0.07	0.28	0.71	0.04	0.14	0.06	0.19	0.12	1.00	0.02	0.18
LKD-248	0.31	0.55	0.23	0.23	0.09	0.12	0.35	0.91	0.04	0.15	0.09	0.28	0.15	1.28	0.03	0.20
LKD-249	0.29	0.52	0.21	0.21	0.05	0.07	0.31	0.80	0.05	0.15	0.07	0.20	0.10	0.87	0.03	0.18
LKD-250	0.03	0.05	0.12	0.12	0.05	0.07	0.08	0.21	0.00	0.00	0.06	0.18	0.02	0.15	0.03	0.23
LKD-251	0.03	0.05	0.16	0.16	0.05	0.07	0.08	0.21	0.01	0.02	0.10	0.31	0.96	8.33	7.18	51.25
LKD-252	0.02	0.03	0.10	0.10	0.05	0.07	0.01	0.04	0.00	0.00	0.07	0.21	0.03	0.30	0.02	0.16
LKD-253	0.02	0.04	0.24	0.24	0.07	0.09	0.03	0.08	0.00	0.00	0.13	0.39	0.01	0.05	0.02	0.15
LKD-254	0.38	0.68	0.19	0.19	0.08	0.11	0.14	0.37	0.10	0.33	0.10	0.31	0.13	1.11	0.04	0.29
LKD-255	0.34	0.62	0.33	0.33	0.08	0.10	0.70	1.80	0.15	0.52	0.09	0.27	0.15	1.29	0.04	0.25
LKD-256	0.19	0.35	0.20	0.20	0.07	0.09	0.15	0.39	0.10	0.33	0.08	0.23	0.12	1.00	0.03	0.20
LKD-257	0.24	0.44	0.12	0.12	0.05	0.07	0.23	0.60	0.04	0.13	0.06	0.18	0.11	0.99	0.03	0.20
LKD-258	0.33	0.59	0.14	0.14	0.06	0.09	0.37	0.96	0.06	0.20	0.09	0.27	0.17	1.43	0.03	0.20
LKD-259	0.26	0.48	0.12	0.12	0.05	0.07	0.19	0.49	0.06	0.19	0.07	0.20	0.12	1.07	0.03	0.20
LKD-260	0.26	0.47	0.21	0.21	0.07	0.10	0.10	0.26	0.03	0.11	0.09	0.28	0.12	1.01	0.03	0.20
LKD-261	0.21	0.37	0.33	0.33	0.09	0.12	0.25	0.65	0.04	0.14	0.09	0.29	0.14	1.18	0.04	0.27
LKD-262	0.44	0.79	0.39	0.39	0.11	0.15	0.37	0.95	0.05	0.16	0.06	0.18	0.21	1.79	0.03	0.23
LKD-263	0.29	0.52	0.24	0.24	0.07	0.10	0.29	0.74	0.05	0.18	0.10	0.32	0.14	1.22	0.04	0.30
LKD-264	0.03	0.05	0.12	0.12	0.05	0.07	0.02	0.06	0.04	0.13	0.08	0.24	0.03	0.26	0.03	0.23
LKD-265	0.04	0.07	0.21	0.21	0.07	0.09	0.04	0.10	0.00	0.00	0.08	0.23	0.03	0.23	0.03	0.23

Continuation of Table S3. Heavy metal contamination factors using both average shale and UCC background values.

	Cu		Pb		Zn		Cr		Ni		Cd		As		Hg	
	Shale	UCC	Shale	UCC	Shale	UCC	Shale	UCC	Shale	UCC	Shale	UCC	Shale	UCC	Shale	UCC*
LKD-266	0.03	0.06	0.13	0.13	0.07	0.09	0.02	0.06	0.01	0.05	0.05	0.15	0.01	0.11	0.03	0.20
LKD-267	0.03	0.05	0.22	0.22	0.07	0.09	0.02	0.06	0.02	0.06	0.07	0.22	0.01	0.11	0.03	0.18
LKD-268	0.44	0.80	0.19	0.19	0.09	0.12	0.07	0.18	0.03	0.11	0.06	0.19	0.11	0.93	0.03	0.23
LKD-269	0.41	0.73	0.27	0.27	0.08	0.11	0.65	1.66	0.11	0.39	0.09	0.29	0.16	1.35	0.03	0.23
LKD-270	0.28	0.50	0.51	0.51	0.11	0.15	0.42	1.08	0.07	0.24	0.07	0.21	0.15	1.31	0.02	0.17
LKD-271	0.24	0.42	1.10	1.10	0.09	0.13	0.32	0.83	0.06	0.21	0.07	0.21	0.13	1.09	0.02	0.18
LKD-272	0.22	0.40	0.41	0.41	0.07	0.10	0.22	0.56	0.03	0.11	0.10	0.32	0.13	1.16	0.03	0.20
LKD-273	0.35	0.64	0.51	0.51	0.08	0.10	0.41	1.06	0.07	0.23	0.06	0.19	0.15	1.33	0.03	0.18
LKD-274	0.03	0.05	0.25	0.25	0.09	0.12	0.03	0.07	0.02	0.06	0.08	0.23	0.01	0.12	0.03	0.18
LKD-275	0.23	0.41	0.55	0.55	0.10	0.13	0.24	0.62	0.13	0.44	0.11	0.35	0.10	0.88	0.03	0.20
LKD-276	0.36	0.65	0.39	0.39	0.10	0.13	0.43	1.10	0.14	0.47	0.17	0.51	0.11	0.91	0.03	0.23
LKD-277	0.34	0.62	0.18	0.18	0.11	0.15	0.34	0.88	0.25	0.84	0.11	0.35	0.13	1.11	0.02	0.18
LKD-278	0.02	0.04	0.19	0.19	0.06	0.08	0.07	0.17	0.02	0.07	0.11	0.34	0.02	0.17	0.03	0.20
LKD-279	0.03	0.06	0.28	0.28	0.07	0.10	0.07	0.19	0.00	0.00	0.12	0.37	0.01	0.12	0.03	0.18
LKD-280	0.03	0.05	0.24	0.24	0.05	0.06	0.08	0.21	0.02	0.08	0.12	0.36	0.01	0.09	0.04	0.25
LKD-281	0.02	0.04	0.33	0.33	0.07	0.09	0.07	0.18	0.14	0.49	0.11	0.34	0.02	0.14	0.02	0.16
LKD-282	0.34	0.61	0.32	0.32	0.03	0.04	0.15	0.37	0.00	0.00	0.09	0.29	0.10	0.86	0.03	0.18
LKD-283	0.38	0.68	0.48	0.48	0.09	0.13	0.65	1.67	0.21	0.73	0.12	0.37	0.11	0.91	0.03	0.18
LKD-284	0.16	0.29	0.39	0.39	0.09	0.12	0.20	0.51	0.00	0.00	0.06	0.19	0.06	0.55	0.02	0.14

Continuation of Table S3. Heavy metal contamination factors using both average shale and UCC background values.

	Cu		Pb		Zn		Cr		Ni		Cd		As		Hg	
	Shale	UCC	Shale	UCC	Shale	UCC	Shale	UCC	Shale	UCC	Shale	UCC	Shale	UCC	Shale	UCC*
LKD-285	0.21	0.38	0.39	0.39	0.07	0.09	0.25	0.64	0.11	0.38	0.08	0.24	0.11	0.93	0.03	0.23
LKD-286	0.28	0.50	0.50	0.50	0.06	0.08	0.26	0.68	0.18	0.61	0.09	0.28	0.12	1.03	0.03	0.21
LKD-287	0.25	0.45	0.16	0.16	0.04	0.05	0.18	0.46	0.20	0.68	0.10	0.31	0.13	1.09	0.03	0.20
LKD-288	0.28	0.51	0.30	0.30	0.05	0.07	0.09	0.24	0.16	0.56	0.10	0.30	0.09	0.75	0.02	0.17
LKD-289	0.25	0.45	0.27	0.27	0.07	0.09	0.26	0.66	0.09	0.32	0.06	0.17	0.16	1.36	0.03	0.20
LKD-290	0.33	0.60	0.25	0.25	0.04	0.05	0.27	0.70	0.04	0.14	0.08	0.24	0.11	0.95	0.03	0.18
LKD-291	0.22	0.40	0.28	0.28	0.06	0.08	0.16	0.42	0.26	0.90	0.08	0.26	0.10	0.88	0.03	0.20
LKD-292	0.03	0.05	0.46	0.46	0.06	0.08	0.05	0.12	0.05	0.16	0.07	0.22	0.03	0.29	0.02	0.17
LKD-293	0.03	0.05	0.26	0.26	0.05	0.06	0.03	0.08	0.04	0.12	0.05	0.14	0.06	0.49	0.03	0.20
LKD-294	0.02	0.04	0.27	0.27	0.06	0.08	0.02	0.06	0.07	0.23	0.05	0.16	0.04	0.31	0.04	0.25
LKD-295	0.03	0.06	0.53	0.53	0.08	0.11	0.04	0.09	0.30	1.01	0.07	0.20	0.04	0.31	0.03	0.20
LKD-296	0.31	0.56	0.36	0.36	0.07	0.09	0.06	0.14	0.14	0.47	0.10	0.31	0.09	0.80	0.03	0.21
LKD-297	0.24	0.44	0.14	0.14	0.08	0.10	0.30	0.78	0.10	0.35	0.08	0.23	0.12	1.01	0.02	0.17
LKD-298	0.16	0.29	0.18	0.18	0.07	0.09	0.20	0.52	0.15	0.53	0.07	0.21	0.09	0.81	0.02	0.16
LKD-299	0.20	0.36	0.21	0.21	0.08	0.11	0.19	0.49	0.24	0.82	0.10	0.32	0.11	0.99	0.02	0.17
LKD-300	0.27	0.49	0.34	0.34	0.11	0.15	0.22	0.57	0.32	1.10	0.11	0.34	0.15	1.27	0.04	0.25
LKD-301	0.30	0.54	0.33	0.33	0.10	0.13	0.20	0.51	0.08	0.27	0.07	0.20	0.15	1.32	0.02	0.17
LKD-302	0.16	0.29	0.42	0.42	0.08	0.11	0.04	0.09	0.10	0.34	0.07	0.21	0.08	0.73	0.03	0.20
LKD-303	0.10	0.19	0.33	0.33	0.07	0.09	0.13	0.34	0.21	0.72	0.10	0.32	0.09	0.77	0.03	0.21

Continuation of Table S3. Heavy metal contamination factors using both average shale and UCC background values.

	Cu		Pb		Zn		Cr		Ni		Cd		As		Hg	
	Shale	UCC	Shale	UCC	Shale	UCC	Shale	UCC	Shale	UCC	Shale	UCC	Shale	UCC	Shale	UCC*
LKD-304	0.16	0.29	0.41	0.41	0.07	0.10	0.23	0.60	0.12	0.40	0.09	0.27	0.12	1.01	0.04	0.25
LKD-305	0.28	0.50	0.53	0.53	0.05	0.07	0.30	0.77	0.15	0.50	0.07	0.21	0.13	1.16	0.02	0.17
LKD-306	0.30	0.54	0.89	0.89	0.10	0.14	0.05	0.14	0.25	0.87	0.07	0.22	0.11	0.97	0.03	0.18
LKD-307	0.22	0.40	0.31	0.31	0.08	0.11	0.27	0.69	0.19	0.66	0.07	0.20	0.12	1.01	0.04	0.27
LKD-308	0.16	0.28	0.30	0.30	0.07	0.10	0.23	0.60	0.09	0.31	0.07	0.22	0.12	1.00	0.04	0.25
LKD-309	0.45	0.81	0.82	0.82	0.34	0.46	0.63	1.62	0.58	1.96	0.13	0.41	0.11	0.93	0.18	1.29

UCC: Upper continental crust value (2); Shale: Average shale values (3); UCC* (4).
Note: To maintain two digits after the decimal, values were rounded.

Table S4. Heavy metal enrichment factors using both average shale and UCC background values.

	Cu		Pb		Cr		Ni		Zn		Cd		As		Hg	
	Shale	UCC	Shale	UCC	Shale	UCC	Shale	UCC	Shale	UCC	Shale	UCC	Shale	UCC	Shale	UCC*
LKD-1	2.27	2.88	2.16	1.52	0.27	0.26	0.47	0.86	0.40	0.96	0.87	1.88	1.01	6.18	0.28	1.40
LKD-2	4.87	6.19	2.35	1.66	0.96	0.91	2.21	4.02	0.40	0.95	1.04	2.24	1.17	7.14	0.32	1.61
LKD-3	1.82	2.31	1.11	0.78	0.14	0.13	1.88	3.42	0.31	0.74	0.65	1.41	0.84	5.17	0.25	1.24
LKD-4	0.30	0.39	19.06	13.45	0.81	0.76	0.50	0.91	0.02	0.06	3.66	7.90	1.24	7.61	0.96	4.84
LKD-5	5.63	7.15	1.87	1.32	1.15	1.09	2.52	4.58	0.33	0.80	1.05	2.27	1.01	6.16	0.34	1.71
LKD-6	0.76	0.96	4.93	3.48	1.16	1.09	3.32	6.02	0.05	0.12	7.93	17.14	4.45	27.20	1.98	10.00
LKD-7	0.07	0.09	0.70	0.50	0.10	0.09	0.10	0.18	0.05	0.12	0.62	1.35	0.47	2.88	0.17	0.86
LKD-8	0.31	0.39	8.65	6.11	0.97	0.91	0.32	0.58	0.01	0.03	1.87	4.05	0.53	3.21	0.38	1.91
LKD-9	4.08	5.18	0.51	0.36	0.58	0.55	1.15	2.09	0.48	1.16	0.94	2.02	1.59	9.72	0.21	1.05
LKD-10	0.09	0.11	0.60	0.42	0.09	0.09	0.11	0.20	0.05	0.12	0.74	1.60	0.62	3.82	0.23	1.15
LKD-11	3.05	3.88	1.21	0.85	0.66	0.62	1.06	1.93	0.22	0.54	0.55	1.19	0.50	3.05	0.18	0.90
LKD-12	6.36	8.08	10.50	7.41	1.51	1.42	1.98	3.59	0.41	1.00	1.20	2.59	1.02	6.26	0.38	1.89
LKD-13	2.27	2.89	1.09	0.77	0.10	0.10	0.97	1.75	0.39	0.94	1.28	2.76	1.35	8.26	0.34	1.73
LKD-14	1.60	2.04	0.74	0.53	0.16	0.15	0.88	1.61	0.31	0.74	1.07	2.30	0.95	5.81	0.32	1.61
LKD-15	0.98	1.24	8.59	6.07	0.20	0.19	0.92	1.67	0.03	0.07	5.05	10.91	1.68	10.26	1.39	6.99
LKD-16	0.14	0.18	8.98	6.34	0.20	0.19	1.96	3.57	0.05	0.12	7.93	17.14	4.40	26.93	2.41	12.14
LKD-17	1.75	2.22	5.75	4.06	1.02	0.96	3.61	6.55	0.76	1.82	7.56	16.33	3.05	18.67	2.13	10.71
LKD-18	0.45	0.57	3.78	2.67	0.75	0.71	1.93	3.50	0.57	1.38	3.27	7.06	1.56	9.54	0.76	3.85
LKD-19	0.64	0.81	6.01	4.24	0.51	0.48	1.19	2.17	0.03	0.08	5.79	12.51	2.81	17.22	1.66	8.39
LKD-20	0.97	1.23	3.91	2.76	0.45	0.43	1.08	1.95	0.05	0.12	7.74	16.73	5.23	32.00	2.13	10.71

Continuation of Table S4. Heavy metal enrichment factors using both average shale and UCC background values.

	Cu		Pb		Cr		Ni		Zn		Cd		As		Hg	
	Shale	UCC	Shale	UCC	Shale	UCC	Shale	UCC	Shale	UCC	Shale	UCC	Shale	UCC	Shale	UCC*
LKD-21	1.17	1.49	6.60	4.66	1.76	1.66	2.59	4.70	0.73	1.76	5.42	11.71	3.07	18.78	1.66	8.39
LKD-22	1.15	1.46	4.31	3.04	0.42	0.40	5.29	9.60	0.52	1.25	16.19	34.99	10.10	61.82	4.69	23.65
LKD-23	1.49	1.89	0.19	0.13	0.09	0.09	1.89	3.43	0.38	0.91	0.64	1.39	0.99	6.03	0.18	0.93
LKD-24	1.02	1.30	0.83	0.58	0.18	0.17	1.55	2.81	0.26	0.63	0.70	1.51	0.57	3.52	0.15	0.73
LKD-25	5.48	6.96	1.47	1.04	1.07	1.01	1.47	2.67	0.44	1.04	1.13	2.45	1.06	6.48	0.30	1.50
LKD-26	4.38	5.56	4.25	3.00	0.89	0.85	0.58	1.06	0.38	0.91	1.13	2.45	0.71	4.33	0.27	1.36
LKD-27	3.91	4.96	0.81	0.57	0.96	0.91	1.15	2.09	0.26	0.63	0.53	1.14	0.58	3.56	0.21	1.07
LKD-28	6.27	7.97	0.09	0.07	0.88	0.83	3.73	6.77	0.89	2.12	1.11	2.39	1.94	11.84	0.30	1.50
LKD-29	3.29	4.18	0.80	0.57	0.41	0.38	6.74	12.24	0.53	1.27	0.85	1.84	1.57	9.60	0.37	1.88
LKD-30	2.08	2.64	0.67	0.47	0.10	0.10	0.70	1.27	0.45	1.07	0.74	1.59	0.91	5.55	0.17	0.86
LKD-31	1.32	1.68	1.17	0.82	0.30	0.28	1.54	2.80	0.56	1.34	0.73	1.58	0.55	3.34	0.16	0.82
LKD-32	2.06	2.62	6.76	4.78	0.18	0.17	2.13	3.86	0.43	1.02	1.11	2.40	0.88	5.37	0.23	1.16
LKD-33	1.97	2.51	1.30	0.92	0.13	0.12	2.13	3.86	0.48	1.15	0.90	1.95	0.66	4.05	0.16	0.80
LKD-34	2.15	2.73	41.73	29.45	0.17	0.17	3.57	6.48	0.88	2.10	1.00	2.17	1.06	6.51	0.23	1.17
LKD-35	0.67	0.85	0.91	0.64	0.07	0.07	0.65	1.18	0.27	0.65	0.55	1.19	0.54	3.29	0.14	0.69
LKD-36	1.30	1.65	1.25	0.88	0.31	0.30	0.90	1.64	0.59	1.41	0.67	1.45	0.67	4.08	0.19	0.94
LKD-37	2.39	3.04	1.60	1.13	0.45	0.43	5.46	9.91	0.57	1.37	1.11	2.40	1.22	7.47	0.32	1.61
LKD-38	1.96	2.49	1.47	1.04	0.19	0.18	1.97	3.58	0.65	1.56	1.26	2.73	1.09	6.69	0.25	1.27
LKD-39	2.86	3.63	0.55	0.39	0.10	0.09	0.59	1.08	0.54	1.31	0.79	1.71	1.10	6.72	0.18	0.93
LKD-40	2.00	2.54	0.55	0.39	0.25	0.23	3.58	6.49	0.46	1.10	0.67	1.44	0.78	4.80	0.19	0.95

Continuation of Table S4. Heavy metal enrichment factors using both average shale and UCC background values.

	Cu		Pb		Cr		Ni		Zn		Cd		As		Hg	
	Shale	UCC	Shale	UCC	Shale	UCC	Shale	UCC	Shale	UCC	Shale	UCC	Shale	UCC	Shale	UCC*
LKD-41	2.40	3.05	1.79	1.26	0.50	0.47	2.41	4.37	0.53	1.28	1.07	2.30	0.88	5.38	0.27	1.38
LKD-42	2.01	2.55	2.16	1.53	0.50	0.47	2.00	3.63	0.45	1.07	1.22	2.63	1.38	8.43	0.32	1.61
LKD-43	2.11	2.68	3.86	2.73	10.33	9.76	12.18	22.11	0.80	1.91	11.04	23.85	3.99	24.42	3.04	15.31
LKD-44	2.25	2.86	5.67	4.00	1.52	1.43	4.12	7.47	1.12	2.68	8.88	19.18	3.75	22.93	1.98	10.00
LKD-45	2.82	3.58	3.31	2.34	2.81	2.66	4.69	8.50	0.84	2.03	12.14	26.24	5.52	33.77	4.14	20.87
LKD-46	0.45	0.57	4.25	3.00	1.34	1.27	1.26	2.29	0.39	0.93	5.05	10.91	1.76	10.78	1.02	5.12
LKD-47	1.58	2.01	0.95	0.67	0.55	0.52	0.84	1.52	0.49	1.17	0.98	2.12	0.93	5.67	0.31	1.56
LKD-48	0.99	1.26	0.69	0.49	0.25	0.23	1.61	2.93	0.25	0.61	0.31	0.66	0.45	2.77	0.14	0.70
LKD-49	0.92	1.17	4.56	3.22	0.13	0.13	1.29	2.34	0.25	0.61	0.48	1.03	0.69	4.21	0.12	0.62
LKD-50	1.58	2.01	16.28	11.49	0.17	0.16	1.88	3.41	0.33	0.79	0.78	1.68	0.85	5.18	0.13	0.67
LKD-51	2.06	2.62	0.96	0.68	0.11	0.11	1.77	3.21	0.55	1.33	1.00	2.17	1.17	7.16	0.23	1.17
LKD-52	1.62	2.06	0.17	0.12	0.09	0.09	0.36	0.65	0.32	0.77	0.39	0.85	0.64	3.90	0.13	0.66
LKD-53	1.14	1.45	2.37	1.67	0.22	0.21	1.31	2.38	0.25	0.59	0.83	1.80	0.51	3.12	0.18	0.93
LKD-54	1.98	2.52	0.75	0.53	0.39	0.37	2.01	3.64	0.44	1.05	0.80	1.73	0.51	3.10	0.27	1.34
LKD-55	1.97	2.50	0.85	0.60	0.37	0.35	2.19	3.97	0.38	0.92	0.94	2.04	0.50	3.03	0.21	1.07
LKD-56	1.01	1.28	1.33	0.94	0.19	0.18	1.38	2.50	0.24	0.57	0.81	1.76	0.36	2.21	0.16	0.79
LKD-57	1.55	1.96	9.66	6.82	4.16	3.93	4.39	7.97	1.04	2.49	15.45	33.40	8.49	51.95	3.04	15.31
LKD-58	2.09	2.66	5.36	3.78	2.10	1.98	3.48	6.32	1.29	3.10	6.61	14.29	0.83	5.07	1.84	9.29
LKD-59	1.06	1.34	5.72	4.04	3.59	3.39	1.40	2.55	0.56	1.34	7.37	15.92	1.57	9.60	1.84	9.29
LKD-60	1.71	2.18	5.64	3.98	3.04	2.87	1.67	3.03	0.67	1.60	5.29	11.43	0.37	2.27	1.70	8.57

Table S4. Heavy metal enrichment factors using both average shale and UCC background values.

	Cu		Pb		Cr		Ni		Zn		Cd		As		Hg	
	Shale	UCC	Shale	UCC	Shale	UCC	Shale	UCC	Shale	UCC	Shale	UCC	Shale	UCC	Shale	UCC*
LKD-61	2.78	3.54	0.29	0.21	0.18	0.17	3.02	5.49	0.57	1.36	0.96	2.08	0.80	4.88	0.14	0.71
LKD-62	3.17	4.02	0.48	0.34	0.37	0.35	0.76	1.38	0.44	1.06	0.92	1.98	0.77	4.74	0.16	0.82
LKD-63	3.06	3.89	1.32	0.93	0.35	0.33	2.37	4.30	0.57	1.37	0.72	1.55	1.06	6.51	0.18	0.93
LKD-64	1.27	1.61	2.50	1.76	0.14	0.13	1.65	2.99	0.28	0.66	0.63	1.37	0.73	4.47	0.18	0.88
LKD-65	1.71	2.17	0.53	0.38	0.28	0.26	1.14	2.08	0.18	0.43	0.44	0.94	0.36	2.20	0.23	1.15
LKD-66	2.22	2.82	0.41	0.29	0.04	0.04	0.51	0.92	0.43	1.03	0.60	1.29	0.72	4.40	0.13	0.68
LKD-67	1.77	2.25	0.45	0.32	0.07	0.07	2.75	4.99	0.36	0.87	0.61	1.33	0.59	3.62	0.12	0.60
LKD-68	1.84	2.34	0.13	0.09	0.11	0.11	1.76	3.19	0.39	0.94	0.83	1.80	0.78	4.77	0.14	0.71
LKD-69	1.64	2.08	2.92	2.06	1.01	0.95	19.96	36.23	3.90	9.36	7.74	16.73	1.79	10.93	1.98	10.00
LKD-70	1.03	1.31	0.21	0.15	0.16	0.15	0.92	1.67	0.25	0.59	0.65	1.40	0.69	4.20	0.17	0.84
LKD-71	2.44	3.10	3.66	2.58	1.04	0.98	20.02	36.34	3.50	8.40	6.23	13.47	1.79	10.93	1.98	10.00
LKD-72	1.94	2.46	2.98	2.10	1.55	1.46	3.58	6.50	1.04	2.49	11.77	25.44	1.27	7.79	2.76	13.91
LKD-73	0.93	1.19	4.95	3.49	1.80	1.70	0.89	1.61	0.76	1.82	2.54	5.49	2.83	17.33	1.25	6.32
LKD-74	0.72	0.91	3.73	2.63	0.89	0.84	1.46	2.65	0.38	0.90	3.66	7.90	0.78	4.77	0.96	4.84
LKD-75	0.84	1.06	2.20	1.55	0.85	0.80	2.28	4.14	0.53	1.27	4.19	9.05	1.59	9.74	1.11	5.59
LKD-76	0.68	0.86	3.15	2.22	0.79	0.75	1.67	3.04	0.40	0.96	5.48	11.84	1.57	9.60	1.70	8.57
LKD-77	3.40	4.32	1.63	1.15	0.71	0.67	1.77	3.21	0.43	1.03	0.98	2.12	0.59	3.64	0.21	1.07
LKD-78	3.66	4.66	1.69	1.19	0.88	0.83	0.58	1.05	0.32	0.77	0.77	1.67	0.50	3.07	0.20	1.00
LKD-79	1.88	2.39	3.44	2.43	0.21	0.20	1.29	2.34	0.48	1.16	1.07	2.30	0.85	5.18	0.28	1.39
LKD-80	1.60	2.03	1.63	1.15	0.12	0.11	1.97	3.57	0.26	0.63	1.63	3.53	0.65	4.00	0.25	1.26

Continuation of Table S4. Heavy metal enrichment factors using both average shale and UCC background values.

	Cu		Pb		Cr		Ni		Zn		Cd		As		Hg	
	Shale	UCC	Shale	UCC	Shale	UCC	Shale	UCC	Shale	UCC	Shale	UCC	Shale	UCC	Shale	UCC*
LKD-81	0.74	0.94	0.14	0.10	0.11	0.10	0.57	1.03	0.16	0.38	0.53	1.15	0.43	2.66	0.12	0.62
LKD-82	2.30	2.92	0.02	0.02	0.13	0.12	0.51	0.93	0.43	1.02	0.85	1.84	0.52	3.20	0.19	0.98
LKD-83	1.52	1.93	0.48	0.34	0.23	0.22	2.48	4.50	0.38	0.92	0.73	1.58	0.44	2.70	0.21	1.07
LKD-84	2.73	3.47	0.55	0.38	0.34	0.32	2.60	4.72	0.54	1.31	0.90	1.94	0.78	4.75	0.28	1.39
LKD-85	1.49	1.89	2.73	1.93	0.09	0.08	1.59	2.88	0.34	0.82	1.03	2.23	0.45	2.73	0.25	1.24
LKD-86	1.84	2.33	0.50	0.36	0.70	0.66	1.63	2.96	0.33	0.79	0.58	1.26	0.65	3.98	0.13	0.65
LKD-87	4.83	6.14	14.30	10.09	3.79	3.58	9.54	17.32	4.16	9.97	11.77	25.44	0.93	5.71	4.14	20.87
LKD-88	2.04	2.59	4.87	3.44	2.15	2.03	4.94	8.96	1.08	2.59	3.56	7.70	0.32	1.94	0.82	4.15
LKD-89	0.23	0.29	5.13	3.62	2.79	2.63	1.94	3.52	0.25	0.60	4.34	9.39	1.57	9.60	1.42	7.14
LKD-90	2.09	2.66	2.78	1.96	1.98	1.87	2.74	4.97	0.05	0.12	6.99	15.10	0.14	0.88	1.30	6.57
LKD-91	3.29	4.18	1.86	1.31	0.54	0.51	2.21	4.01	7.91	18.99	1.33	2.88	0.75	4.61	0.33	1.64
LKD-92	3.01	3.83	2.03	1.44	0.21	0.20	0.87	1.58	8.40	20.15	1.53	3.31	1.05	6.43	0.34	1.74
LKD-93	1.24	1.58	0.61	0.43	0.15	0.14	0.76	1.38	5.54	13.30	0.85	1.84	0.52	3.17	0.21	1.07
LKD-94	1.55	1.97	1.28	0.90	0.28	0.26	1.81	3.28	16.54	39.69	1.34	2.89	0.98	6.00	0.33	1.68
LKD-95	1.03	1.31	0.50	0.35	0.17	0.16	0.61	1.10	4.34	10.42	0.70	1.51	0.30	1.81	0.17	0.86
LKD-96	2.23	2.84	0.37	0.26	0.12	0.11	0.54	0.98	4.80	11.51	0.55	1.19	0.58	3.55	0.13	0.67
LKD-97	1.19	1.52	0.45	0.32	0.00	0.00	1.95	3.54	0.31	0.73	0.15	0.33	0.46	2.83	0.18	0.93
LKD-98	1.74	2.22	1.15	0.81	0.08	0.08	1.91	3.47	0.26	0.63	0.13	0.28	1.15	7.01	0.44	2.23
LKD-99	1.55	1.96	0.63	0.44	0.00	0.00	2.47	4.49	0.23	0.55	0.05	0.12	0.70	4.25	0.23	1.17
LKD-100	1.05	1.33	0.86	0.60	0.00	0.00	0.99	1.81	0.17	0.40	0.38	0.82	0.40	2.45	0.20	1.00

Continuation of Table S4. Heavy metal enrichment factors using both average shale and UCC background values.

	Cu		Pb		Cr		Ni		Zn		Cd		As		Hg	
	Shale	UCC	Shale	UCC	Shale	UCC	Shale	UCC	Shale	UCC	Shale	UCC	Shale	UCC	Shale	UCC*
LKD-101	0.67	0.85	7.62	5.38	0.02	0.02	1.95	3.53	0.05	0.12	0.38	0.82	2.79	17.07	1.84	9.29
LKD-102	0.18	0.22	8.47	5.98	0.16	0.15	0.61	1.11	0.02	0.04	0.13	0.28	0.28	1.74	0.74	3.73
LKD-103	5.03	6.39	10.49	7.40	0.03	0.03	3.32	6.03	0.10	0.23	0.81	1.75	1.53	9.35	4.14	20.87
LKD-104	1.77	2.25	3.38	2.38	0.00	0.00	1.51	2.74	0.15	0.35	0.55	1.19	0.68	4.16	0.39	1.95
LKD-105	2.69	3.42	3.65	2.58	0.12	0.11	0.69	1.25	0.38	0.91	0.47	1.01	1.05	6.40	0.40	2.02
LKD-106	1.94	2.46	0.88	0.62	0.00	0.00	0.99	1.80	1.99	4.77	0.94	2.04	0.85	5.20	0.30	1.50
LKD-107	0.89	1.13	1.10	0.77	0.00	0.00	1.44	2.62	0.36	0.87	0.20	0.44	0.85	5.20	0.32	1.61
LKD-108	1.20	1.52	0.44	0.31	0.09	0.08	0.97	1.76	0.57	1.37	0.04	0.09	0.56	3.41	0.18	0.93
LKD-119	2.04	2.60	0.71	0.50	0.13	0.12	0.35	0.64	0.48	1.15	0.46	1.00	0.84	5.13	0.27	1.36
LKD-110	1.92	2.44	0.99	0.70	0.59	0.56	2.54	4.61	0.38	0.90	0.59	1.28	0.68	4.15	0.21	1.07
LKD-111	1.49	1.90	1.78	1.26	0.03	0.03	1.47	2.66	0.30	0.72	0.16	0.35	0.64	3.91	0.35	1.76
LKD-112	6.72	8.54	1.45	1.03	1.40	1.32	1.85	3.35	0.24	0.58	0.07	0.15	0.96	5.89	0.27	1.38
LKD-113	1.23	1.56	0.93	0.66	0.00	0.00	1.09	1.98	0.18	0.44	0.43	0.92	0.57	3.49	0.18	0.92
LKD-114	1.32	1.68	0.95	0.67	0.03	0.03	1.32	2.40	0.24	0.57	0.19	0.42	0.42	2.58	0.32	1.61
LKD-115	0.62	0.78	17.13	12.09	0.61	0.58	1.86	3.37	3.83	9.20	0.37	0.80	0.77	4.70	1.39	6.99
LKD-116	1.84	2.34	40.57	28.64	0.03	0.03	2.38	4.32	2.84	6.82	8.83	19.08	4.59	28.05	4.14	20.87
LKD-117	0.46	0.58	9.02	6.37	0.59	0.55	0.85	1.55	0.16	0.38	0.60	1.30	0.21	1.30	0.65	3.26
LKD-118	1.47	1.87	2.44	1.72	0.32	0.30	1.34	2.43	0.29	0.69	0.15	0.33	0.65	3.96	0.31	1.56
LKD-119	2.72	3.45	2.16	1.53	0.01	0.01	1.27	2.30	0.37	0.88	1.36	2.93	1.79	10.96	0.65	3.26
LKD-120	2.43	3.09	3.05	2.15	0.89	0.84	3.16	5.74	0.60	1.45	0.48	1.03	1.50	9.19	0.44	2.23

Continuation of Table S4. Heavy metal enrichment factors using both average shale and UCC background values.

	Cu		Pb		Cr		Ni		Zn		Cd		As		Hg	
	Shale	UCC	Shale	UCC	Shale	UCC	Shale	UCC	Shale	UCC	Shale	UCC	Shale	UCC	Shale	UCC*
LKD-121	1.58	2.00	1.08	0.76	0.00	0.00	2.02	3.67	0.50	1.20	0.48	1.03	1.17	7.17	0.41	2.09
LKD-122	0.87	1.10	0.54	0.38	0.00	0.00	0.84	1.52	0.24	0.58	0.63	1.37	0.54	3.32	0.21	1.07
LKD-123	1.64	2.08	0.63	0.44	0.00	0.00	0.28	0.51	0.29	0.70	0.43	0.94	0.71	4.35	0.16	0.79
LKD-124	1.62	2.06	0.70	0.50	0.00	0.00	2.42	4.39	0.20	0.49	0.52	1.11	0.90	5.53	0.27	1.36
LKD-125	1.58	2.01	1.69	1.19	0.04	0.03	1.31	2.38	0.31	0.74	0.87	1.87	1.00	6.12	0.43	2.14
LKD-126	1.46	1.86	0.90	0.64	0.00	0.00	1.72	3.12	0.17	0.41	0.46	1.00	1.06	6.51	0.23	1.17
LKD-127	1.44	1.82	0.47	0.33	0.09	0.09	1.61	2.93	0.24	0.57	0.05	0.10	0.21	1.28	0.18	0.93
LKD-128	0.76	0.96	12.01	8.48	0.02	0.02	1.18	2.15	0.05	0.11	2.46	5.31	7.98	48.80	2.13	10.71
LKD-129	2.01	2.56	31.52	22.25	0.03	0.03	0.78	1.42	0.09	0.22	11.77	25.44	6.11	37.40	3.31	16.70
LKD-130	0.23	0.29	3.33	2.35	0.34	0.32	0.40	0.73	0.22	0.52	0.04	0.10	0.07	0.43	0.23	1.16
LKD-131	0.09	0.12	57.95	40.91	4.11	3.89	3.25	5.90	1.23	2.96	0.66	1.43	4.59	28.05	4.14	20.87
LKD-132	1.80	2.28	1.38	0.97	0.26	0.24	1.91	3.47	0.34	0.81	0.34	0.72	0.89	5.42	0.32	1.61
LKD-133	1.80	2.29	1.24	0.87	0.21	0.20	0.84	1.52	0.40	0.96	0.28	0.61	0.87	5.31	0.23	1.17
LKD-134	1.98	2.52	1.84	1.30	0.21	0.19	2.66	4.82	0.47	1.14	0.06	0.14	1.28	7.86	0.35	1.76
LKD-135	1.85	2.36	1.71	1.21	0.09	0.08	1.99	3.62	0.56	1.35	0.90	1.95	0.93	5.71	0.21	1.07
LKD-136	1.03	1.31	1.07	0.75	0.18	0.17	1.60	2.91	0.38	0.90	0.14	0.31	0.41	2.53	0.17	0.85
LKD-137	1.79	2.27	1.59	1.12	0.32	0.31	0.48	0.88	0.41	0.98	0.11	0.24	0.67	4.12	0.15	0.76
LKD-138	1.23	1.57	0.51	0.36	0.00	0.00	1.99	3.61	0.26	0.63	0.42	0.90	0.60	3.68	0.14	0.69
LKD-139	1.38	1.76	1.21	0.85	0.20	0.19	1.21	2.19	0.28	0.68	0.62	1.35	0.68	4.16	0.26	1.29
LKD-140	1.50	1.90	0.91	0.64	0.27	0.25	1.28	2.33	0.24	0.56	0.22	0.47	1.03	6.28	0.26	1.29

Continuation of Table S4. Heavy metal enrichment factors using both average shale and UCC background values.

	Cu		Pb		Cr		Ni		Zn		Cd		As		Hg	
	Shale	UCC	Shale	UCC	Shale	UCC	Shale	UCC	Shale	UCC	Shale	UCC	Shale	UCC	Shale	UCC*
LKD-141	1.12	1.42	0.78	0.55	0.06	0.06	0.78	1.42	0.22	0.52	0.28	0.61	1.05	6.43	0.17	0.84
LKD-142	1.10	1.40	24.95	17.61	1.38	1.31	4.69	8.50	0.10	0.23	0.63	1.35	5.01	30.65	3.04	15.31
LKD-143	1.62	2.06	33.06	23.34	5.09	4.81	2.27	4.12	0.10	0.23	6.62	14.31	4.25	25.97	3.59	18.09
LKD-144	0.11	0.14	11.42	8.06	1.32	1.25	0.14	0.26	0.03	0.08	0.25	0.53	2.25	13.74	1.02	5.12
LKD-145	0.02	0.03	1.90	1.34	0.23	0.22	0.02	0.04	0.00	0.01	0.08	0.17	0.08	0.49	0.16	0.82
LKD-146	1.54	1.95	0.02	0.01	0.00	0.00	2.05	3.73	0.27	0.66	0.04	0.08	0.90	5.52	0.16	0.79
LKD-147	1.13	1.43	0.73	0.51	0.00	0.00	0.08	0.14	0.28	0.67	0.07	0.14	0.75	4.56	0.30	1.51
LKD-148	1.39	1.77	0.93	0.66	0.41	0.39	1.10	1.99	0.50	1.20	0.06	0.14	1.00	6.11	0.34	1.73
LKD-149	0.52	0.66	0.25	0.18	0.12	0.11	0.46	0.84	0.12	0.28	0.12	0.25	0.33	2.00	0.24	1.21
LKD-150	0.65	0.83	0.29	0.20	0.00	0.00	0.79	1.43	0.19	0.46	0.04	0.08	0.36	2.21	0.18	0.88
LKD-151	1.71	2.18	0.02	0.01	0.00	0.00	0.09	0.17	0.34	0.81	0.03	0.07	0.75	4.60	0.23	1.14
LKD-152	1.31	1.67	0.14	0.10	0.00	0.00	2.14	3.89	0.32	0.76	0.03	0.07	0.54	3.29	0.17	0.83
LKD-153	1.89	2.40	0.44	0.31	0.17	0.16	1.94	3.52	0.33	0.79	0.04	0.09	0.92	5.65	0.21	1.07
LKD-154	1.15	1.46	0.11	0.08	0.27	0.26	1.13	2.06	0.16	0.37	2.28	4.93	1.15	7.01	0.33	1.67
LKD-155	0.64	0.81	1.38	0.97	0.13	0.12	0.91	1.65	0.17	0.40	0.03	0.07	0.40	2.44	0.12	0.58
LKD-156	0.14	0.18	8.87	6.26	1.09	1.03	0.22	0.40	0.05	0.12	0.47	1.02	1.35	8.27	2.13	10.71
LKD-157	0.29	0.37	4.74	3.35	0.78	0.73	0.46	0.83	0.13	0.30	0.41	0.88	0.55	3.39	0.60	3.03
LKD-158	0.54	0.69	0.12	0.09	0.64	0.60	4.88	8.86	0.51	1.23	0.06	0.14	0.18	1.08	0.28	1.40
LKD-159	0.66	0.84	0.35	0.25	0.16	0.15	0.67	1.21	0.26	0.64	0.17	0.36	0.46	2.83	0.30	1.52
LKD-160	1.62	2.06	1.35	0.95	1.26	1.19	1.07	1.94	1.11	2.68	0.05	0.11	0.94	5.73	1.08	5.45

Continuation of Table S4. Heavy metal enrichment factors using both average shale and UCC background values.

	Cu		Pb		Cr		Ni		Zn		Cd		As		Hg	
	Shale	UCC	Shale	UCC	Shale	UCC	Shale	UCC	Shale	UCC	Shale	UCC	Shale	UCC	Shale	UCC*
LKD-161	1.26	1.61	0.63	0.44	0.82	0.78	6.67	12.10	0.35	0.83	0.06	0.13	1.54	9.44	0.53	2.68
LKD-162	0.72	0.92	0.21	0.15	0.22	0.21	0.65	1.18	0.18	0.42	0.03	0.07	0.41	2.51	0.20	1.02
LKD-163	1.76	2.23	0.02	0.01	0.51	0.49	0.39	0.71	0.40	0.95	0.11	0.24	0.69	4.22	0.25	1.24
LKD-164	1.37	1.74	0.16	0.11	0.53	0.50	2.20	4.00	0.31	0.75	0.07	0.14	0.66	4.04	0.30	1.50
LKD-165	1.71	2.17	0.51	0.36	0.29	0.27	1.82	3.30	0.42	1.00	0.86	1.86	0.76	4.66	0.09	0.43
LKD-166	1.34	1.70	0.23	0.17	0.00	0.00	1.57	2.86	0.35	0.85	0.05	0.10	0.80	4.87	0.21	1.07
LKD-167	0.84	1.07	0.02	0.01	0.00	0.00	1.03	1.87	0.16	0.38	0.04	0.09	1.13	6.89	0.23	1.15
LKD-168	0.83	1.05	0.05	0.03	0.09	0.09	1.01	1.83	0.26	0.62	0.03	0.06	0.37	2.26	0.12	0.61
LKD-169	0.09	0.11	0.41	0.29	0.22	0.21	0.04	0.06	0.05	0.13	0.06	0.14	0.41	2.49	0.32	1.61
LKD-170	0.07	0.09	0.38	0.27	0.49	0.46	0.12	0.21	0.11	0.27	0.14	0.29	2.43	14.87	0.69	3.49
LKD-171	0.76	0.96	0.18	0.13	0.05	0.05	0.97	1.77	0.19	0.45	0.04	0.08	0.68	4.15	0.17	0.87
LKD-172	1.47	1.87	0.56	0.39	0.04	0.04	0.20	0.36	0.28	0.66	0.04	0.09	1.29	7.87	0.33	1.64
LKD-173	1.36	1.73	0.31	0.22	0.05	0.05	2.46	4.46	0.23	0.56	0.11	0.23	0.97	5.93	0.30	1.52
LKD-174	1.39	1.76	0.03	0.02	0.20	0.19	0.65	1.18	0.27	0.65	1.77	3.82	1.83	11.22	0.44	2.23
LKD-175	1.19	1.52	0.02	0.02	0.09	0.09	1.36	2.47	0.24	0.57	0.05	0.12	0.79	4.83	0.23	1.16
LKD-176	1.89	2.40	0.02	0.02	0.14	0.13	2.48	4.50	0.63	1.50	0.05	0.10	1.27	7.77	0.28	1.43
LKD-177	1.35	1.72	0.51	0.36	0.25	0.24	1.42	2.59	0.44	1.05	0.05	0.11	1.12	6.87	0.21	1.07
LKD-178	1.96	2.49	0.02	0.01	0.06	0.06	2.12	3.84	0.44	1.05	0.04	0.09	1.22	7.49	0.24	1.22
LKD-179	3.36	4.26	0.15	0.11	0.67	0.64	0.25	0.45	0.28	0.68	0.05	0.10	0.69	4.22	0.23	1.15
LKD-180	0.77	0.97	0.72	0.51	0.32	0.30	1.11	2.02	0.33	0.79	0.03	0.07	0.63	3.87	0.15	0.77

Continuation of Table S4. Heavy metal enrichment factors using both average shale and UCC background values.

	Cu		Pb		Cr		Ni		Zn		Cd		As		Hg	
	Shale	UCC	Shale	UCC	Shale	UCC	Shale	UCC	Shale	UCC	Shale	UCC	Shale	UCC	Shale	UCC*
LKD-181	0.12	0.15	1.23	0.87	0.74	0.70	0.07	0.13	0.03	0.06	0.03	0.06	0.26	1.57	0.23	1.15
LKD-182	0.07	0.09	4.80	3.39	0.51	0.49	0.69	1.26	0.22	0.52	4.20	9.08	0.51	3.10	1.03	5.18
LKD-183	0.44	0.55	3.38	2.38	0.47	0.44	4.41	8.00	0.68	1.62	0.15	0.31	1.26	7.69	1.09	5.49
LKD-184	0.21	0.26	3.85	2.72	2.89	2.73	0.62	1.13	0.59	1.42	0.20	0.43	1.12	6.84	1.23	6.22
LKD-185	0.83	1.05	2.48	1.75	0.46	0.43	0.87	1.58	0.17	0.40	0.04	0.09	0.57	3.50	0.16	0.80
LKD-186	1.94	2.47	0.22	0.16	0.24	0.23	1.96	3.56	0.39	0.94	0.05	0.11	1.70	10.37	0.39	1.99
LKD-187	1.04	1.32	0.03	0.02	0.16	0.15	0.56	1.01	0.27	0.65	1.50	3.24	1.25	7.64	0.30	1.50
LKD-188	1.37	1.74	0.03	0.02	0.82	0.77	1.90	3.45	0.39	0.93	0.11	0.24	0.97	5.92	0.33	1.67
LKD-189	1.97	2.50	0.56	0.40	0.33	0.31	3.04	5.51	0.53	1.28	0.83	1.79	0.86	5.23	0.16	0.83
LKD-190	1.28	1.63	0.52	0.37	0.30	0.28	0.94	1.70	0.15	0.36	0.45	0.96	0.69	4.20	0.18	0.92
LKD-191	1.83	2.32	0.86	0.61	0.43	0.41	1.85	3.36	0.50	1.21	0.43	0.92	1.12	6.83	0.16	0.82
LKD-192	2.68	3.40	0.99	0.70	0.54	0.51	2.98	5.41	0.57	1.37	0.46	1.00	1.28	7.82	0.19	0.97
LKD-193	2.01	2.56	0.94	0.66	0.56	0.53	1.50	2.73	0.93	2.24	0.59	1.28	0.86	5.27	0.21	1.07
LKD-194	1.62	2.06	0.92	0.65	0.41	0.39	2.49	4.53	0.61	1.46	0.35	0.77	0.99	6.03	0.21	1.07
LKD-195	0.69	0.87	5.24	3.70	1.68	1.58	0.63	1.15	0.02	0.06	1.46	3.16	0.63	3.87	0.75	3.80
LKD-196	1.26	1.60	9.92	7.00	3.42	3.23	1.39	2.51	0.05	0.12	4.34	9.39	1.18	7.20	1.70	8.57
LKD-197	0.46	0.58	8.79	6.21	1.49	1.40	1.54	2.79	0.43	1.03	1.60	3.45	0.27	1.64	0.54	2.75
LKD-198	0.70	0.89	9.47	6.69	1.94	1.83	3.11	5.64	0.61	1.47	2.19	4.74	0.51	3.10	0.69	3.46
LKD-199	1.92	2.44	1.17	0.82	0.61	0.57	1.33	2.42	0.07	0.16	1.13	2.45	0.37	2.29	0.21	1.07
LKD-200	2.60	3.30	0.83	0.59	0.41	0.38	0.72	1.30	0.04	0.09	0.50	1.07	0.78	4.77	0.19	0.98

Continuation of Table S4. Heavy metal enrichment factors using both average shale and UCC background values.

	Cu		Pb		Cr		Ni		Zn		Cd		As		Hg	
	Shale	UCC	Shale	UCC	Shale	UCC	Shale	UCC	Shale	UCC	Shale	UCC	Shale	UCC	Shale	UCC*
LKD-201	1.42	1.81	0.90	0.64	0.55	0.52	2.09	3.79	0.71	1.70	0.59	1.28	0.57	3.49	0.23	1.17
LKD-202	1.96	2.49	0.95	0.67	0.53	0.50	2.05	3.72	0.42	1.02	0.82	1.78	0.84	5.16	0.23	1.15
LKD-203	2.01	2.55	1.02	0.72	0.50	0.47	3.53	6.41	0.58	1.40	0.44	0.95	0.96	5.89	0.21	1.07
LKD-204	1.35	1.72	0.57	0.40	0.33	0.32	1.22	2.22	0.36	0.86	0.44	0.95	0.49	2.98	0.11	0.54
LKD-205	1.73	2.20	1.06	0.75	0.60	0.57	1.26	2.29	0.22	0.52	0.70	1.51	1.15	7.05	0.27	1.38
LKD-206	1.54	1.96	1.19	0.84	0.62	0.59	0.50	0.91	0.01	0.02	0.60	1.29	0.56	3.40	0.19	0.94
LKD-207	0.86	1.10	1.80	1.27	0.55	0.52	0.77	1.40	0.01	0.03	0.97	2.10	0.75	4.57	0.29	1.47
LKD-208	2.16	2.75	1.25	0.88	0.40	0.38	2.34	4.24	0.26	0.63	0.39	0.83	1.11	6.80	0.25	1.27
LKD-209	1.28	1.63	9.15	6.46	3.08	2.91	1.89	3.43	2.10	5.04	3.59	7.76	1.13	6.93	1.35	6.79
LKD-210	0.34	0.44	21.07	14.87	1.40	1.32	2.42	4.39	0.96	2.31	0.79	1.71	0.15	0.95	0.22	1.09
LKD-211	1.36	1.73	8.22	5.80	2.73	2.58	1.39	2.51	2.58	6.18	3.21	6.94	0.83	5.07	1.36	6.86
LKD-212	0.95	1.21	3.25	2.30	1.68	1.59	3.32	6.02	0.03	0.08	2.71	5.86	0.31	1.91	1.29	6.52
LKD-213	0.85	1.08	0.36	0.26	0.20	0.19	1.63	2.96	0.05	0.11	0.34	0.74	0.41	2.53	0.11	0.55
LKD-214	2.25	2.87	0.77	0.54	0.27	0.25	0.54	0.99	0.43	1.03	0.32	0.69	0.76	4.68	0.12	0.60
LKD-215	2.23	2.83	1.22	0.86	0.92	0.87	6.62	12.02	1.04	2.51	0.61	1.31	1.05	6.43	0.15	0.74
LKD-216	1.57	2.00	1.27	0.90	0.34	0.32	1.33	2.41	0.26	0.64	0.24	0.51	0.59	3.63	0.19	0.98
LKD-217	1.08	1.37	2.06	1.45	0.59	0.56	2.96	5.37	0.45	1.08	0.57	1.22	0.98	6.00	0.20	0.99
LKD-218	1.49	1.90	0.52	0.37	0.32	0.31	1.84	3.35	0.40	0.96	0.28	0.61	0.64	3.94	0.09	0.47
LKD-219	1.71	2.18	1.20	0.84	0.48	0.46	2.10	3.81	0.20	0.48	0.28	0.61	0.80	4.89	0.20	0.99
LKD-220	1.50	1.90	0.98	0.69	0.39	0.37	0.99	1.79	0.13	0.31	0.30	0.65	0.72	4.40	0.23	1.14

Continuation of Table S4. Heavy metal enrichment factors using both average shale and UCC background values.

	Cu		Pb		Cr		Ni		Zn		Cd		As		Hg	
	Shale	UCC	Shale	UCC	Shale	UCC	Shale	UCC	Shale	UCC	Shale	UCC	Shale	UCC	Shale	UCC*
LKD-221	1.28	1.63	0.99	0.70	0.37	0.35	2.22	4.03	0.01	0.02	0.45	0.98	0.95	5.84	0.21	1.07
LKD-222	1.43	1.82	0.91	0.65	0.28	0.26	1.83	3.32	0.31	0.73	0.22	0.48	0.70	4.27	0.13	0.68
LKD-223	0.53	0.67	2.66	1.88	1.02	0.96	2.11	3.83	0.67	1.62	1.45	3.14	0.60	3.69	0.51	2.58
LKD-224	1.50	1.90	5.87	4.14	2.57	2.43	4.58	8.31	0.05	0.12	3.59	7.76	2.22	13.60	1.36	6.86
LKD-225	0.87	1.10	3.30	2.33	1.69	1.59	3.11	5.64	2.58	6.20	1.92	4.15	0.36	2.19	0.69	3.46
LKD-226	0.48	0.61	2.55	1.80	0.90	0.85	2.05	3.73	1.25	3.00	1.48	3.21	0.45	2.73	0.44	2.21
LKD-227	0.70	0.89	1.57	1.11	0.37	0.35	1.98	3.59	0.34	0.82	0.51	1.10	0.47	2.88	0.16	0.79
LKD-228	1.78	2.26	1.76	1.24	0.96	0.91	4.19	7.61	0.51	1.22	0.37	0.79	0.65	3.98	0.10	0.49
LKD-229	1.50	1.90	1.08	0.77	0.53	0.50	2.58	4.69	0.10	0.25	0.54	1.17	0.56	3.40	0.23	1.16
LKD-230	1.67	2.12	1.71	1.21	0.75	0.71	1.99	3.61	0.48	1.14	0.54	1.17	0.54	3.33	0.25	1.25
LKD-231	1.13	1.44	5.10	3.60	0.19	0.18	1.22	2.22	0.29	0.70	0.28	0.61	0.68	4.16	0.10	0.50
LKD-232	1.62	2.06	1.30	0.92	0.42	0.40	1.54	2.80	0.45	1.09	0.91	1.97	0.98	5.98	0.18	0.92
LKD-233	1.41	1.79	1.52	1.07	0.32	0.30	0.79	1.44	0.32	0.76	0.47	1.02	0.46	2.80	0.10	0.51
LKD-234	1.82	2.32	1.09	0.77	0.53	0.50	3.56	6.45	0.52	1.25	0.60	1.30	1.41	8.61	0.24	1.21
LKD-235	2.11	2.68	0.87	0.62	0.43	0.41	1.91	3.48	0.71	1.69	0.64	1.39	0.59	3.64	0.18	0.93
LKD-236	0.55	0.70	5.39	3.81	1.28	1.21	1.68	3.05	0.02	0.05	1.67	3.61	0.54	3.28	0.47	2.36
LKD-237	0.72	0.92	4.82	3.40	2.05	1.93	3.55	6.45	1.18	2.84	2.83	6.12	0.51	3.13	1.11	5.59
LKD-238	2.33	2.96	18.93	13.36	5.94	5.61	10.78	19.57	3.26	7.83	10.67	23.06	5.52	33.77	3.86	19.48
LKD-239	0.96	1.22	7.09	5.00	1.98	1.87	1.08	1.97	0.02	0.06	2.01	4.34	0.57	3.48	0.69	3.46
LKD-240	1.53	1.95	2.60	1.83	0.40	0.38	0.58	1.06	0.96	2.30	0.43	0.92	0.51	3.11	0.12	0.60

Continuation of Table S4. Heavy metal enrichment factors using both average shale and UCC background values.

	Cu		Pb		Cr		Ni		Zn		Cd		As		Hg	
	Shale	UCC	Shale	UCC	Shale	UCC	Shale	UCC	Shale	UCC	Shale	UCC	Shale	UCC	Shale	UCC*
LKD-241	1.60	2.03	0.84	0.60	0.27	0.25	2.64	4.79	0.23	0.55	0.42	0.90	0.89	5.47	0.13	0.64
LKD-242	0.79	1.00	0.63	0.44	0.23	0.22	1.28	2.33	0.28	0.68	0.24	0.53	0.34	2.09	0.14	0.73
LKD-243	1.54	1.96	0.78	0.55	0.44	0.42	2.40	4.35	0.59	1.41	0.68	1.46	0.76	4.65	0.16	0.82
LKD-244	1.50	1.91	1.43	1.01	0.45	0.43	2.32	4.20	1.07	2.57	0.34	0.74	0.65	4.00	0.16	0.79
LKD-245	2.13	2.70	1.92	1.36	0.36	0.34	1.48	2.69	0.44	1.06	0.57	1.22	0.96	5.87	0.23	1.16
LKD-246	1.68	2.14	1.57	1.11	0.42	0.40	1.84	3.34	0.50	1.20	0.57	1.22	0.72	4.40	0.25	1.25
LKD-247	1.30	1.65	0.87	0.62	0.33	0.31	1.82	3.30	0.27	0.65	0.41	0.89	0.75	4.62	0.16	0.81
LKD-248	2.17	2.76	1.62	1.15	0.62	0.58	2.49	4.53	0.31	0.75	0.64	1.38	1.05	6.40	0.19	0.98
LKD-249	2.03	2.58	1.48	1.05	0.38	0.36	2.21	4.01	0.32	0.77	0.47	1.02	0.71	4.37	0.18	0.89
LKD-250	1.71	2.18	6.89	4.86	2.76	2.61	4.56	8.29	0.05	0.12	3.40	7.35	0.96	5.87	1.84	9.29
LKD-251	0.43	0.55	2.44	1.72	0.83	0.79	1.27	2.30	0.11	0.27	1.57	3.40	15.14	92.59	112.94	569.44
LKD-252	1.43	1.81	8.08	5.70	3.90	3.68	1.08	1.96	0.07	0.16	5.41	11.69	2.67	16.36	1.72	8.67
LKD-253	0.76	0.97	8.96	6.33	2.62	2.47	1.09	1.98	0.03	0.08	4.68	10.12	0.23	1.41	0.76	3.82
LKD-254	2.01	2.55	0.99	0.70	0.42	0.40	0.76	1.38	0.52	1.24	0.53	1.15	0.68	4.15	0.21	1.07
LKD-255	1.12	1.42	1.09	0.77	0.25	0.23	2.28	4.15	0.50	1.20	0.28	0.61	0.49	2.98	0.11	0.58
LKD-256	1.02	1.29	1.04	0.73	0.36	0.34	0.81	1.48	0.52	1.25	0.41	0.88	0.61	3.75	0.15	0.74
LKD-257	1.47	1.87	0.72	0.51	0.33	0.31	1.42	2.57	0.22	0.54	0.36	0.79	0.70	4.26	0.17	0.84
LKD-258	1.64	2.09	0.69	0.48	0.32	0.31	1.86	3.38	0.29	0.69	0.43	0.94	0.83	5.06	0.14	0.69
LKD-259	1.87	2.38	0.85	0.60	0.39	0.37	1.36	2.47	0.40	0.96	0.47	1.02	0.88	5.37	0.19	0.98
LKD-260	1.71	2.18	1.39	0.98	0.47	0.44	0.67	1.22	0.22	0.52	0.59	1.27	0.76	4.68	0.18	0.91

Continuation of Table S4. Heavy metal enrichment factors using both average shale and UCC background values.

	Cu		Pb		Cr		Ni		Zn		Cd		As		Hg	
	Shale	UCC	Shale	UCC	Shale	UCC	Shale	UCC	Shale	UCC	Shale	UCC	Shale	UCC	Shale	UCC*
LKD-261	1.35	1.72	2.13	1.50	0.56	0.53	1.64	2.98	0.28	0.66	0.61	1.32	0.89	5.45	0.25	1.24
LKD-262	1.96	2.49	1.75	1.23	0.51	0.48	1.65	3.00	0.21	0.51	0.27	0.58	0.93	5.66	0.15	0.73
LKD-263	1.87	2.38	1.54	1.09	0.49	0.46	1.88	3.42	0.34	0.81	0.68	1.46	0.92	5.63	0.28	1.40
LKD-264	1.59	2.02	6.57	4.64	3.11	2.94	1.22	2.21	2.12	5.08	4.53	9.80	1.70	10.40	1.84	9.29
LKD-265	1.44	1.83	7.78	5.49	2.41	2.27	1.38	2.50	0.03	0.08	2.83	6.12	0.97	5.91	1.20	6.06
LKD-266	1.98	2.51	7.08	5.00	3.82	3.61	1.36	2.47	0.78	1.88	2.83	6.12	0.74	4.53	1.56	7.86
LKD-267	0.20	0.25	1.54	1.09	0.47	0.44	0.17	0.32	0.12	0.28	0.52	1.12	0.09	0.57	0.18	0.89
LKD-268	2.36	3.00	1.02	0.72	0.48	0.45	0.37	0.68	0.17	0.41	0.34	0.73	0.57	3.50	0.17	0.87
LKD-269	1.57	2.00	1.05	0.74	0.32	0.30	2.50	4.54	0.44	1.06	0.36	0.78	0.60	3.69	0.13	0.63
LKD-270	1.03	1.31	1.87	1.32	0.40	0.38	1.55	2.82	0.26	0.62	0.26	0.56	0.56	3.41	0.09	0.44
LKD-271	0.91	1.16	4.23	2.99	0.37	0.35	1.25	2.28	0.23	0.56	0.27	0.58	0.49	2.98	0.10	0.48
LKD-272	1.18	1.50	2.18	1.54	0.40	0.37	1.15	2.09	0.18	0.42	0.55	1.19	0.71	4.35	0.15	0.74
LKD-273	2.00	2.54	2.89	2.04	0.44	0.42	2.34	4.25	0.39	0.93	0.36	0.78	0.87	5.31	0.14	0.71
LKD-274	0.20	0.26	1.77	1.25	0.61	0.58	0.19	0.35	0.13	0.31	0.54	1.17	0.10	0.60	0.18	0.89
LKD-275	1.14	1.45	2.73	1.92	0.50	0.47	1.21	2.20	0.64	1.54	0.57	1.22	0.51	3.11	0.14	0.69
LKD-276	2.55	3.24	2.75	1.94	0.69	0.65	3.04	5.51	0.99	2.37	1.18	2.55	0.75	4.57	0.23	1.16
LKD-277	1.95	2.48	1.03	0.73	0.63	0.59	1.94	3.52	1.40	3.36	0.64	1.39	0.73	4.45	0.14	0.71
LKD-278	1.72	2.18	13.74	9.70	4.46	4.21	4.64	8.43	1.35	3.25	7.79	16.84	1.36	8.33	1.95	9.82
LKD-279	0.99	1.26	8.91	6.29	2.33	2.20	2.36	4.28	0.03	0.07	3.78	8.16	0.44	2.67	0.79	3.97
LKD-280	2.89	3.68	26.05	18.39	4.98	4.71	8.90	16.16	2.73	6.55	12.88	27.83	1.19	7.27	3.86	19.48

Continuation of Table S4. Heavy metal enrichment factors using both average shale and UCC background values.

	Cu		Pb		Cr		Ni		Zn		Cd		As		Hg	
	Shale	UCC	Shale	UCC	Shale	UCC	Shale	UCC	Shale	UCC	Shale	UCC	Shale	UCC	Shale	UCC*
LKD-281	0.92	1.17	12.34	8.71	2.41	2.27	2.64	4.80	5.30	12.73	4.07	8.78	0.60	3.65	0.81	4.10
LKD-282	2.39	3.04	2.24	1.58	0.24	0.22	1.03	1.87	0.01	0.02	0.66	1.43	0.70	4.30	0.18	0.89
LKD-283	2.29	2.91	2.94	2.07	0.57	0.54	3.95	7.16	1.30	3.13	0.73	1.57	0.64	3.91	0.15	0.77
LKD-284	1.15	1.46	2.79	1.97	0.62	0.59	1.41	2.56	0.01	0.02	0.45	0.97	0.45	2.77	0.14	0.70
LKD-285	1.62	2.06	3.02	2.13	0.51	0.48	1.91	3.48	0.85	2.05	0.62	1.34	0.83	5.09	0.25	1.27
LKD-286	1.69	2.14	3.02	2.13	0.37	0.35	1.60	2.90	1.08	2.59	0.55	1.18	0.72	4.43	0.18	0.92
LKD-287	1.92	2.44	1.26	0.89	0.28	0.26	1.38	2.51	1.53	3.68	0.77	1.67	0.97	5.93	0.21	1.07
LKD-288	2.01	2.56	2.13	1.50	0.38	0.36	0.65	1.18	1.17	2.80	0.68	1.48	0.62	3.77	0.17	0.84
LKD-289	1.52	1.94	1.66	1.17	0.41	0.39	1.56	2.83	0.56	1.35	0.34	0.74	0.95	5.83	0.17	0.84
LKD-290	2.35	2.98	1.75	1.24	0.28	0.26	1.92	3.49	0.29	0.70	0.57	1.22	0.77	4.73	0.18	0.89
LKD-291	1.56	1.98	1.99	1.41	0.42	0.40	1.16	2.10	1.88	4.50	0.59	1.28	0.72	4.40	0.19	0.98
LKD-292	3.24	4.11	50.34	35.53	6.82	6.44	4.99	9.06	5.13	12.31	8.10	17.49	3.74	22.86	2.65	13.36
LKD-293	1.73	2.19	14.54	10.26	2.68	2.53	1.87	3.39	2.02	4.84	2.64	5.71	3.23	19.73	1.56	7.86
LKD-294	1.35	1.71	15.44	10.90	3.30	3.12	1.26	2.29	3.89	9.34	3.02	6.53	2.01	12.27	1.98	10.00
LKD-295	0.93	1.18	14.53	10.26	2.31	2.18	0.99	1.80	8.10	19.45	1.83	3.95	0.99	6.06	0.75	3.80
LKD-296	1.78	2.26	2.02	1.43	0.39	0.37	0.32	0.58	0.78	1.87	0.57	1.22	0.52	3.20	0.17	0.86
LKD-297	1.73	2.20	0.96	0.68	0.54	0.51	2.16	3.91	0.73	1.75	0.54	1.17	0.83	5.07	0.17	0.84
LKD-298	0.92	1.17	1.03	0.73	0.39	0.36	1.15	2.09	0.88	2.10	0.40	0.86	0.53	3.25	0.12	0.63
LKD-299	1.72	2.19	1.81	1.28	0.71	0.67	1.62	2.95	2.04	4.89	0.88	1.90	0.97	5.92	0.20	1.01
LKD-300	1.29	1.64	1.63	1.15	0.52	0.49	1.05	1.91	1.53	3.67	0.52	1.12	0.69	4.24	0.17	0.83

Continuation of Table S4. Heavy metal enrichment factors using both average shale and UCC background values.

	Cu		Pb		Cr		Ni		Zn		Cd		As		Hg	
	Shale	UCC	Shale	UCC	Shale	UCC	Shale	UCC	Shale	UCC	Shale	UCC	Shale	UCC	Shale	UCC*
LKD-301	2.11	2.68	2.32	1.64	0.70	0.66	1.42	2.57	0.57	1.36	0.47	1.02	1.08	6.60	0.17	0.87
LKD-302	1.07	1.36	2.74	1.94	0.53	0.50	0.23	0.42	0.66	1.58	0.46	0.99	0.55	3.35	0.18	0.91
LKD-303	0.95	1.21	3.02	2.13	0.62	0.59	1.21	2.19	1.92	4.61	0.94	2.04	0.82	4.99	0.27	1.38
LKD-304	0.80	1.02	2.03	1.43	0.37	0.35	1.17	2.13	0.58	1.39	0.43	0.94	0.58	3.55	0.18	0.88
LKD-305	2.52	3.20	4.80	3.39	0.50	0.47	2.74	4.98	1.34	3.22	0.64	1.38	1.22	7.48	0.22	1.11
LKD-306	1.95	2.47	5.82	4.11	0.68	0.64	0.34	0.63	1.66	3.99	0.48	1.04	0.73	4.49	0.16	0.82
LKD-307	1.56	1.99	2.16	1.53	0.57	0.54	1.90	3.44	1.38	3.30	0.47	1.02	0.82	5.03	0.27	1.34
LKD-308	0.79	1.00	1.51	1.06	0.37	0.35	1.16	2.11	0.45	1.08	0.37	0.79	0.58	3.53	0.18	0.88
LKD-309	2.73	3.46	4.95	3.49	2.09	1.97	3.83	6.96	3.49	8.38	0.81	1.75	0.65	4.00	1.09	5.51

UCC: Upper continental crust value (2); Shale: Average shale values (3); UCC* (4).
Note: To maintain two digits after the decimal, values were rounded.

Table S5. Geoaccumulation index of heavy metal(loid)s.

	Cu		Pb		Zn		Cr		Ni		Cd		As		Hg	
	Shale	UCC	Shale	UCC	Shale	UCC	Shale	UCC	Shale	UCC	Shale	UCC	Shale	UCC	Shale	UCC*
LKD1	-2.11	-1.27	-2.18	-2.18	-5.17	-4.75	-4.37	-3.01	-4.62	-2.86	-3.49	-1.88	-3.28	-0.16	-5.14	-2.30
LKD2	-1.49	-0.64	-2.54	-2.54	-3.83	-3.41	-2.63	-1.27	-5.11	-3.34	-3.73	-2.11	-3.55	0.73	-5.42	-2.58
LKD3	-2.43	-1.58	-3.15	-3.15	-6.13	-5.71	-2.38	-1.02	-4.99	-3.22	-3.91	-2.29	-3.54	0.75	-5.32	-2.49
LKD4	-7.08	-6.23	-1.11	-1.11	-5.67	-5.25	-6.35	-4.99	-10.73	-8.97	-3.49	-1.88	-5.05	-0.76	-5.42	-2.58
LKD5	-1.18	-0.33	-2.77	-2.77	-3.47	-3.05	-2.34	-0.98	-5.26	-3.49	-3.60	-1.99	-3.66	0.62	-5.23	-2.39
LKD6	-6.81	-5.97	-4.11	-4.11	-6.20	-5.78	-4.68	-3.32	-10.73	-8.97	-3.42	-1.81	-4.26	0.03	-5.42	-2.58
LKD7	-6.84	-5.99	-3.60	-3.60	-6.42	-6.00	-6.42	-5.05	-7.41	-5.64	-3.77	-2.16	-4.17	0.11	-5.64	-2.81
LKD8	-6.21	-5.36	-1.40	-1.40	-4.56	-4.14	-6.15	-4.79	-10.73	-8.97	-3.60	-1.99	-5.44	-1.15	-5.91	-3.07
LKD9	-1.64	-0.80	-4.63	-4.63	-4.45	-4.03	-3.47	-2.11	-4.72	-2.95	-3.77	-2.16	-3.00	1.28	-5.94	-3.10
LKD10	-6.79	-5.94	-4.04	-4.04	-6.69	-6.27	-6.47	-5.11	-7.62	-5.85	-3.73	-2.11	-3.98	0.31	-5.42	-2.58
LKD11	-1.14	-0.29	-2.47	-2.47	-3.35	-2.93	-2.66	-1.29	-4.90	-3.14	-3.60	-1.99	-3.75	0.54	-5.23	-2.39
LKD12	-1.24	-0.39	-0.51	-0.51	-3.32	-2.90	-2.92	-1.56	-5.18	-3.41	-3.64	-2.03	-3.87	0.41	-5.32	-2.49
LKD13	-2.59	-1.74	-3.66	-3.66	-7.06	-6.64	-3.83	-2.46	-5.13	-3.37	-3.42	-1.81	-3.34	0.94	-5.32	-2.49
LKD14	-3.10	-2.25	-4.20	-4.20	-6.42	-6.00	-3.95	-2.59	-5.47	-3.71	-3.68	-2.07	-3.85	0.43	-5.42	-2.58
LKD15	-5.83	-4.98	-2.69	-2.69	-8.10	-7.68	-5.91	-4.55	-10.81	-9.04	-3.46	-1.84	-5.05	-0.76	-5.32	-2.49
LKD16	-9.26	-8.41	-3.24	-3.24	-8.71	-8.29	-5.44	-4.07	-10.73	-8.97	-3.42	-1.81	-4.27	0.01	-5.14	-2.30
LKD17	-5.60	-4.75	-3.89	-3.89	-6.38	-5.96	-4.56	-3.20	-6.81	-5.04	-3.49	-1.88	-4.80	-0.51	-5.32	-2.49
LKD18	-6.20	-5.35	-3.11	-3.11	-5.45	-5.03	-4.08	-2.72	-5.83	-4.07	-3.32	-1.71	-4.39	-0.10	-5.42	-2.58
LKD19	-6.44	-5.59	-3.21	-3.21	-6.77	-6.35	-5.54	-4.17	-10.73	-8.97	-3.26	-1.65	-4.30	-0.01	-5.06	-2.22
LKD20	-6.45	-5.61	-4.44	-4.44	-7.55	-7.13	-6.30	-4.94	-10.73	-8.97	-3.46	-1.84	-4.02	0.26	-5.32	-2.49
LKD21	-5.56	-4.71	-3.07	-3.07	-4.98	-4.56	-4.42	-3.06	-6.24	-4.47	-3.35	-1.74	-4.17	0.11	-5.06	-2.22
LKD22	-7.17	-6.32	-5.27	-5.27	-8.63	-8.21	-4.97	-3.61	-8.32	-6.55	-3.35	-1.74	-4.03	0.25	-5.14	-2.30
LKD23	-2.52	-1.67	-5.51	-5.51	-6.51	-6.09	-2.17	-0.81	-4.48	-2.71	-3.73	-2.11	-3.11	1.18	-5.53	-2.69
LKD24	-2.71	-1.86	-3.02	-3.02	-5.20	-4.77	-2.12	-0.76	-4.67	-2.90	-3.26	-1.65	-3.55	0.74	-5.53	-2.69
LKD25	-1.22	-0.37	-3.12	-3.12	-3.57	-3.15	-3.11	-1.75	-4.87	-3.11	-3.49	-1.88	-3.59	0.70	-5.42	-2.58
LKD26	-1.40	-0.56	-1.45	-1.45	-3.70	-3.28	-4.32	-2.96	-4.93	-3.16	-3.35	-1.74	-4.03	0.25	-5.42	-2.58
LKD27	-0.57	0.28	-2.84	-2.84	-2.59	-2.17	-2.33	-0.97	-4.47	-2.71	-3.46	-1.84	-3.31	0.97	-4.77	-1.93

Continuation of Table S5. Geoaccumulation index of heavy metal(loid)s.

	Cu		Pb		Zn		Cr		Ni		Cd		As		Hg	
	Shale	UCC	Shale	UCC	Shale	UCC	Shale	UCC	Shale	UCC	Shale	UCC	Shale	UCC	Shale	UCC*
LKD28	-1.02	-0.18	-7.09	-7.09	-3.85	-3.43	-1.77	-0.41	-3.85	-2.08	-3.53	-1.91	-2.72	1.57	-5.42	-2.58
LKD29	-1.69	-0.84	-3.72	-3.72	-4.71	-4.29	-0.66	0.71	-4.33	-2.57	-3.64	-2.03	-2.76	1.53	-4.84	-2.00
LKD30	-2.03	-1.18	-3.66	-3.66	-6.36	-5.94	-3.60	-2.24	-4.25	-2.48	-3.53	-1.91	-3.23	1.06	-5.64	-2.81
LKD31	-2.50	-1.66	-2.68	-2.68	-4.66	-4.24	-2.28	-0.92	-3.75	-1.98	-3.35	-1.74	-3.78	0.51	-5.53	-2.69
LKD32	-2.37	-1.52	-0.65	-0.65	-5.89	-5.47	-2.32	-0.96	-4.64	-2.88	-3.26	-1.65	-3.60	0.69	-5.53	-2.69
LKD33	-2.02	-1.17	-2.61	-2.61	-5.98	-5.56	-1.91	-0.54	-4.06	-2.29	-3.14	-1.53	-3.59	0.70	-5.64	-2.81
LKD34	-2.43	-1.58	1.85	1.85	-6.05	-5.63	-1.70	-0.34	-3.73	-1.96	-3.53	-1.91	-3.45	0.84	-5.64	-2.81
LKD35	-3.48	-2.64	-3.04	-3.04	-6.72	-6.30	-3.53	-2.17	-4.79	-3.02	-3.77	-2.16	-3.80	0.49	-5.77	-2.93
LKD36	-2.62	-1.77	-2.67	-2.67	-4.66	-4.24	-3.14	-1.78	-3.76	-2.00	-3.57	-1.95	-3.58	0.70	-5.42	-2.58
LKD37	-2.15	-1.30	-2.73	-2.73	-4.55	-4.13	-0.96	0.40	-4.22	-2.45	-3.26	-1.65	-3.12	1.16	-5.06	-2.22
LKD38	-2.57	-1.72	-2.98	-2.98	-5.93	-5.51	-2.55	-1.19	-4.15	-2.39	-3.20	-1.58	-3.41	0.88	-5.53	-2.69
LKD39	-1.57	-0.72	-3.96	-3.96	-6.41	-5.99	-3.84	-2.48	-3.97	-2.20	-3.42	-1.81	-2.95	1.33	-5.53	-2.69
LKD40	-1.91	-1.06	-3.77	-3.77	-4.93	-4.51	-1.07	0.29	-4.03	-2.26	-3.49	-1.88	-3.26	1.03	-5.32	-2.49
LKD41	-2.52	-1.67	-2.94	-2.94	-4.79	-4.37	-2.51	-1.15	-4.68	-2.91	-3.68	-2.07	-3.96	0.32	-5.64	-2.81
LKD42	-2.77	-1.92	-2.67	-2.67	-4.79	-4.37	-2.78	-1.41	-4.94	-3.17	-3.49	-1.88	-3.31	0.97	-5.42	-2.58
LKD43	-6.29	-5.45	-5.42	-5.42	-4.00	-3.58	-3.77	-2.40	-7.70	-5.94	-3.91	-2.29	-5.37	-1.09	-5.77	-2.93
LKD44	-5.24	-4.39	-3.91	-3.91	-5.81	-5.39	-4.37	-3.00	-6.25	-4.48	-3.26	-1.65	-4.50	-0.22	-5.42	-2.58
LKD45	-5.88	-5.03	-5.64	-5.64	-5.88	-5.46	-5.14	-3.78	-7.62	-5.85	-3.77	-2.16	-4.91	-0.62	-5.32	-2.49
LKD46	-6.94	-6.09	-3.71	-3.71	-5.37	-4.95	-5.46	-4.10	-7.17	-5.40	-3.46	-1.84	-4.98	-0.69	-5.77	-2.93
LKD47	-2.87	-2.03	-3.61	-3.61	-4.39	-3.97	-3.79	-2.42	-4.57	-2.81	-3.57	-1.95	-3.64	0.64	-5.23	-2.39
LKD48	-2.31	-1.46	-2.83	-2.83	-4.31	-3.89	-1.60	-0.24	-4.27	-2.51	-4.01	-2.39	-3.44	0.85	-5.14	-2.30
LKD49	-2.86	-2.02	-0.56	-0.56	-5.65	-5.23	-2.38	-1.02	-4.72	-2.96	-3.81	-2.20	-3.29	1.00	-5.77	-2.93
LKD50	-2.33	-1.48	1.03	1.03	-5.54	-5.12	-2.09	-0.72	-4.60	-2.84	-3.35	-1.74	-3.24	1.05	-5.91	-3.07
LKD51	-2.49	-1.64	-3.59	-3.59	-6.69	-6.27	-2.71	-1.35	-4.39	-2.62	-3.53	-1.91	-3.31	0.98	-5.64	-2.81
LKD52	-1.91	-1.06	-5.14	-5.14	-6.02	-5.60	-4.09	-2.72	-4.25	-2.48	-3.96	-2.34	-3.25	1.04	-5.53	-2.69
LKD53	-2.90	-2.05	-1.85	-1.85	-5.27	-4.85	-2.70	-1.34	-5.11	-3.34	-3.35	-1.74	-4.06	0.23	-5.53	-2.69
LKD54	-2.42	-1.57	-3.82	-3.82	-4.75	-4.33	-2.40	-1.04	-4.60	-2.84	-3.73	-2.11	-4.39	-0.10	-5.32	-2.49

Continuation of Table S5. Geoaccumulation index of heavy metal(loid)s.

	Cu		Pb		Zn		Cr		Ni		Cd		As		Hg	
	Shale	UCC	Shale	UCC	Shale	UCC	Shale	UCC	Shale	UCC	Shale	UCC	Shale	UCC	Shale	UCC*
LKD55	-2.43	-1.58	-3.65	-3.65	-4.86	-4.44	-2.28	-0.92	-4.79	-3.03	-3.49	-1.88	-4.42	-0.14	-5.64	-2.81
LKD56	-3.08	-2.23	-2.68	-2.68	-5.47	-5.05	-2.62	-1.26	-5.16	-3.40	-3.39	-1.77	-4.55	-0.27	-5.77	-2.93
LKD57	-6.74	-5.90	-4.10	-4.10	-5.31	-4.89	-5.24	-3.87	-7.32	-5.55	-3.42	-1.81	-4.29	0.00	-5.77	-2.93
LKD58	-5.35	-4.50	-3.99	-3.99	-5.34	-4.92	-4.61	-3.25	-6.04	-4.27	-3.68	-2.07	-6.68	-2.40	-5.53	-2.69
LKD59	-6.33	-5.48	-3.89	-3.89	-4.57	-4.14	-5.92	-4.56	-7.25	-5.48	-3.53	-1.91	-5.76	-1.47	-5.53	-2.69
LKD60	-5.63	-4.79	-3.91	-3.91	-4.81	-4.39	-5.67	-4.31	-6.99	-5.23	-4.01	-2.39	-7.84	-3.56	-5.64	-2.81
LKD61	-1.61	-0.76	-4.85	-4.85	-5.55	-5.13	-1.49	-0.13	-3.91	-2.15	-3.14	-1.53	-3.41	0.87	-5.91	-3.07
LKD62	-1.63	-0.78	-4.34	-4.34	-4.73	-4.31	-3.68	-2.32	-4.47	-2.71	-3.42	-1.81	-3.66	0.62	-5.91	-3.07
LKD63	-1.47	-0.63	-2.69	-2.69	-4.60	-4.18	-1.84	-0.48	-3.89	-2.13	-3.57	-1.95	-3.00	1.29	-5.53	-2.69
LKD64	-2.57	-1.72	-1.58	-1.58	-5.77	-5.35	-2.18	-0.82	-4.77	-3.00	-3.57	-1.95	-3.36	0.93	-5.42	-2.58
LKD65	-1.52	-0.67	-3.20	-3.20	-4.14	-3.72	-2.10	-0.74	-4.77	-3.00	-3.49	-1.88	-3.77	0.52	-4.42	-1.58
LKD66	-1.60	-0.75	-4.02	-4.02	-7.28	-6.86	-3.73	-2.36	-3.97	-2.20	-3.49	-1.88	-3.22	1.06	-5.64	-2.81
LKD67	-2.00	-1.15	-3.97	-3.97	-6.67	-6.25	-1.37	0.00	-4.28	-2.52	-3.53	-1.91	-3.58	0.70	-5.91	-3.07
LKD68	-2.21	-1.36	-6.00	-6.00	-6.24	-5.82	-2.27	-0.91	-4.44	-2.67	-3.35	-1.74	-3.45	0.84	-5.91	-3.07
LKD69	-5.70	-4.85	-4.86	-4.86	-6.40	-5.98	-2.09	-0.73	-4.45	-2.68	-3.46	-1.84	-5.57	-1.29	-5.42	-2.58
LKD70	-3.15	-2.30	-5.44	-5.44	-5.83	-5.41	-3.31	-1.95	-5.21	-3.44	-3.81	-2.20	-3.73	0.56	-5.77	-2.93
LKD71	-5.12	-4.27	-4.54	-4.54	-6.36	-5.94	-2.09	-0.72	-4.60	-2.84	-3.77	-2.16	-5.57	-1.29	-5.42	-2.58
LKD72	-6.42	-5.57	-5.80	-5.80	-6.74	-6.32	-5.53	-4.17	-7.32	-5.55	-3.81	-2.20	-7.02	-2.74	-5.91	-3.07
LKD73	-5.13	-4.28	-2.72	-2.72	-4.18	-3.76	-5.20	-3.84	-5.43	-3.66	-3.68	-2.07	-3.53	0.76	-4.71	-1.87
LKD74	-5.84	-4.99	-3.46	-3.46	-5.53	-5.11	-4.81	-3.45	-6.78	-5.01	-3.49	-1.88	-5.72	-1.43	-5.42	-2.58
LKD75	-6.05	-5.20	-4.66	-4.66	-6.02	-5.60	-4.60	-3.24	-6.72	-4.95	-3.73	-2.11	-5.12	-0.84	-5.64	-2.81
LKD76	-6.97	-6.12	-4.76	-4.76	-6.74	-6.32	-5.67	-4.30	-7.73	-5.97	-3.96	-2.34	-5.76	-1.47	-5.64	-2.81
LKD77	-1.77	-0.92	-2.83	-2.83	-4.04	-3.62	-2.71	-1.35	-4.75	-2.98	-3.57	-1.95	-4.29	0.00	-5.77	-2.93
LKD78	-1.21	-0.37	-2.33	-2.33	-3.28	-2.86	-3.87	-2.51	-4.72	-2.96	-3.46	-1.84	-4.08	0.20	-5.42	-2.58
LKD79	-2.99	-2.15	-2.12	-2.12	-6.15	-5.73	-3.54	-2.18	-4.95	-3.19	-3.81	-2.20	-4.15	0.14	-5.77	-2.93
LKD80	-3.23	-2.38	-3.21	-3.21	-6.95	-6.53	-2.93	-1.57	-5.84	-4.08	-3.20	-1.58	-4.52	-0.23	-5.91	-3.07
LKD81	-3.34	-2.49	-5.77	-5.77	-6.13	-5.71	-3.73	-2.36	-5.57	-3.80	-3.81	-2.20	-4.11	0.18	-5.94	-3.10

Continuation of Table S5. Geoaccumulation index of heavy metal(loid)s.

	Cu		Pb		Zn		Cr		Ni		Cd		As		Hg	
	Shale	UCC	Shale	UCC	Shale	UCC	Shale	UCC	Shale	UCC	Shale	UCC	Shale	UCC	Shale	UCC*
LKD82	-2.21	-1.36	-8.97	-8.97	-6.41	-5.99	-4.37	-3.01	-4.64	-2.88	-3.64	-2.03	-4.34	-0.06	-5.77	-2.93
LKD83	-2.81	-1.96	-4.47	-4.47	-5.52	-5.10	-2.10	-0.74	-4.80	-3.03	-3.86	-2.25	-4.59	-0.30	-5.64	-2.81
LKD84	-2.46	-1.61	-4.78	-4.78	-5.45	-5.03	-2.53	-1.17	-4.78	-3.02	-4.06	-2.44	-4.27	0.01	-5.77	-2.93
LKD85	-3.33	-2.48	-2.46	-2.46	-7.41	-6.99	-3.24	-1.88	-5.46	-3.69	-3.86	-2.25	-5.07	-0.79	-5.94	-3.10
LKD86	-1.95	-1.10	-3.82	-3.82	-3.34	-2.92	-2.12	-0.76	-4.43	-2.67	-3.60	-1.99	-3.45	0.84	-5.77	-2.93
LKD87	-5.10	-4.25	-3.53	-3.53	-5.45	-5.03	-4.12	-2.75	-5.32	-3.55	-3.81	-2.20	-7.47	-3.18	-5.32	-2.49
LKD88	-4.34	-3.49	-3.08	-3.08	-4.26	-3.84	-3.06	-1.70	-5.25	-3.48	-3.53	-1.91	-7.02	-2.74	-5.64	-2.81
LKD89	-8.55	-7.70	-4.05	-4.05	-4.93	-4.51	-5.45	-4.09	-8.41	-6.64	-4.29	-2.68	-5.76	-1.47	-5.91	-3.07
LKD90	-5.35	-4.50	-4.94	-4.94	-5.42	-5.00	-4.96	-3.59	-10.73	-8.97	-3.60	-1.99	-9.21	-4.92	-6.03	-3.19
LKD91	-2.19	-1.34	-3.01	-3.01	-4.80	-4.38	-2.76	-1.40	-0.92	0.84	-3.49	-1.88	-4.31	-0.03	-5.53	-2.69
LKD92	-2.52	-1.67	-3.08	-3.08	-6.36	-5.94	-4.30	-2.94	-1.04	0.73	-3.49	-1.88	-4.03	0.25	-5.64	-2.81
LKD93	-3.10	-2.25	-4.12	-4.12	-6.16	-5.74	-3.81	-2.44	-0.94	0.83	-3.64	-2.03	-4.36	-0.07	-5.64	-2.81
LKD94	-3.56	-2.71	-3.84	-3.84	-6.02	-5.60	-3.33	-1.97	-0.14	1.63	-3.77	-2.16	-4.22	0.07	-5.77	-2.93
LKD95	-3.04	-2.19	-4.10	-4.10	-5.63	-5.21	-3.81	-2.45	-0.97	0.80	-3.60	-1.99	-4.84	-0.56	-5.64	-2.81
LKD96	-1.84	-0.99	-4.41	-4.41	-6.06	-5.64	-3.88	-2.52	-0.73	1.03	-3.86	-2.25	-3.78	0.51	-5.91	-3.07
LKD97	-2.83	-1.98	-4.23	-4.23	-12.21	-11.79	-2.12	-0.76	-4.80	-3.04	-5.81	-4.20	-4.20	0.08	-5.53	-2.69
LKD98	-3.25	-2.40	-3.85	-3.85	-7.63	-7.21	-3.11	-1.75	-5.98	-4.21	-7.01	-5.39	-3.85	0.43	-5.23	-2.39
LKD99	-2.91	-2.06	-4.21	-4.21	-12.21	-11.79	-2.23	-0.87	-5.67	-3.90	-7.74	-6.13	-4.06	0.23	-5.64	-2.81
LKD100	-3.02	-2.17	-3.31	-3.31	-12.21	-11.79	-3.09	-1.73	-5.66	-3.89	-4.49	-2.88	-4.41	-0.12	-5.42	-2.58
LKD101	-6.99	-6.14	-3.48	-3.48	-12.21	-11.79	-5.45	-4.09	-10.73	-8.97	-7.81	-6.20	-4.93	-0.64	-5.53	-2.69
LKD102	-7.29	-6.45	-1.71	-1.71	-7.42	-7.00	-5.50	-4.14	-10.73	-8.97	-7.74	-6.13	-6.61	-2.32	-5.23	-2.39
LKD103	-5.04	-4.19	-3.98	-3.98	-12.21	-11.79	-5.64	-4.28	-10.73	-8.97	-7.68	-6.06	-6.76	-2.47	-5.32	-2.49
LKD104	-3.22	-2.37	-2.29	-2.29	-12.21	-11.79	-3.46	-2.09	-6.81	-5.04	-4.91	-3.29	-4.61	-0.32	-5.42	-2.58
LKD105	-2.48	-1.63	-2.04	-2.04	-6.95	-6.53	-4.44	-3.08	-5.30	-3.53	-5.01	-3.39	-3.84	0.44	-5.23	-2.39
LKD106	-2.82	-1.97	-3.96	-3.96	-12.21	-11.79	-3.79	-2.43	-2.78	-1.02	-3.86	-2.25	-4.01	0.28	-5.53	-2.69
LKD107	-3.95	-3.10	-3.64	-3.64	-12.21	-11.79	-3.25	-1.89	-5.24	-3.47	-6.07	-4.46	-4.01	0.28	-5.42	-2.58
LKD108	-2.83	-1.98	-4.27	-4.27	-6.57	-6.15	-3.13	-1.77	-3.90	-2.13	-7.74	-6.13	-3.93	0.36	-5.53	-2.69

Continuation of Table S5. Geoaccumulation index of heavy metal(loid)s.

	Cu		Pb		Zn		Cr		Ni		Cd		As		Hg	
	Shale	UCC	Shale	UCC	Shale	UCC	Shale	UCC	Shale	UCC	Shale	UCC	Shale	UCC	Shale	UCC*
LKD109	-2.50	-1.66	-4.03	-4.03	-6.52	-6.10	-5.03	-3.67	-4.60	-2.84	-4.64	-3.03	-3.79	0.50	-5.42	-2.58
LKD110	-2.59	-1.74	-3.55	-3.55	-4.29	-3.87	-2.19	-0.83	-4.95	-3.18	-4.29	-2.68	-4.10	0.19	-5.77	-2.93
LKD111	-3.33	-2.48	-3.08	-3.08	-8.75	-8.33	-3.35	-1.99	-5.65	-3.89	-6.52	-4.91	-4.55	-0.27	-5.42	-2.58
LKD112	-1.03	-0.18	-3.24	-3.24	-3.30	-2.88	-2.89	-1.53	-5.83	-4.07	-7.61	-6.00	-3.83	0.45	-5.64	-2.81
LKD113	-2.89	-2.04	-3.29	-3.29	-12.21	-11.79	-3.06	-1.70	-5.64	-3.88	-4.42	-2.81	-4.00	0.29	-5.64	-2.81
LKD114	-3.37	-2.52	-3.86	-3.86	-8.63	-8.21	-3.38	-2.01	-5.85	-4.08	-6.14	-4.52	-5.02	-0.74	-5.42	-2.58
LKD115	-6.49	-5.64	-1.69	-1.69	-6.50	-6.08	-4.90	-3.54	-3.85	-2.09	-7.23	-5.61	-6.17	-1.89	-5.32	-2.49
LKD116	-6.49	-5.64	-2.03	-2.03	-12.21	-11.79	-6.12	-4.76	-5.87	-4.10	-4.23	-2.61	-5.17	-0.89	-5.32	-2.49
LKD117	-5.93	-5.08	-1.62	-1.62	-5.56	-5.14	-5.02	-3.66	-7.46	-5.69	-5.52	-3.91	-7.02	-2.74	-5.42	-2.58
LKD118	-2.98	-2.13	-2.25	-2.25	-5.20	-4.78	-3.11	-1.75	-5.34	-3.57	-6.23	-4.61	-4.16	0.12	-5.23	-2.39
LKD119	-3.35	-2.50	-3.68	-3.68	-12.21	-11.79	-4.45	-3.09	-6.24	-4.47	-4.35	-2.74	-3.95	0.33	-5.42	-2.58
LKD120	-2.77	-1.92	-2.44	-2.44	-4.22	-3.80	-2.39	-1.02	-4.78	-3.01	-5.11	-3.50	-3.46	0.82	-5.23	-2.39
LKD121	-3.39	-2.54	-3.94	-3.94	-12.21	-11.79	-3.03	-1.67	-5.05	-3.29	-5.11	-3.50	-3.82	0.46	-5.32	-2.49
LKD122	-3.50	-2.65	-4.18	-4.18	-12.21	-11.79	-3.55	-2.19	-5.33	-3.57	-3.96	-2.34	-4.17	0.11	-5.53	-2.69
LKD123	-2.38	-1.53	-3.76	-3.76	-12.21	-11.79	-4.93	-3.56	-4.87	-3.11	-4.29	-2.68	-3.58	0.70	-5.77	-2.93
LKD124	-2.84	-1.99	-4.04	-4.04	-12.21	-11.79	-2.26	-0.90	-5.82	-4.06	-4.49	-2.88	-3.68	0.60	-5.42	-2.58
LKD125	-3.24	-2.39	-3.15	-3.15	-8.67	-8.25	-3.52	-2.15	-5.60	-3.84	-4.11	-2.50	-3.91	0.38	-5.14	-2.30
LKD126	-2.99	-2.14	-3.68	-3.68	-12.21	-11.79	-2.75	-1.39	-6.09	-4.32	-4.64	-3.03	-3.45	0.84	-5.64	-2.81
LKD127	-2.57	-1.72	-4.18	-4.18	-6.49	-6.07	-2.40	-1.04	-5.16	-3.39	-7.49	-5.88	-5.34	-1.06	-5.53	-2.69
LKD128	-6.81	-5.97	-2.82	-2.82	-12.21	-11.79	-6.17	-4.80	-10.86	-9.09	-5.11	-3.50	-3.41	0.87	-5.32	-2.49
LKD129	-6.36	-5.52	-2.39	-2.39	-12.21	-11.79	-7.72	-6.36	-10.83	-9.07	-3.81	-2.20	-4.76	-0.47	-5.64	-2.81
LKD130	-5.52	-4.67	-1.68	-1.68	-4.96	-4.54	-4.73	-3.37	-5.62	-3.86	-7.89	-6.27	-7.23	-2.94	-5.53	-2.69
LKD131	-10.79	-9.95	-1.51	-1.51	-5.33	-4.91	-5.67	-4.31	-7.07	-5.30	-7.97	-6.35	-5.17	-0.89	-5.32	-2.49
LKD132	-2.93	-2.08	-3.31	-3.31	-5.73	-5.31	-2.84	-1.48	-5.35	-3.58	-5.35	-3.74	-3.95	0.33	-5.42	-2.58
LKD133	-2.68	-1.84	-3.23	-3.23	-5.77	-5.35	-3.79	-2.43	-4.85	-3.09	-5.35	-3.74	-3.74	0.55	-5.64	-2.81
LKD134	-2.92	-2.07	-3.03	-3.03	-6.18	-5.76	-2.50	-1.14	-4.99	-3.22	-7.89	-6.27	-3.55	0.74	-5.42	-2.58
LKD135	-2.64	-1.80	-2.76	-2.76	-7.03	-6.61	-2.54	-1.18	-4.36	-2.60	-3.68	-2.07	-3.63	0.65	-5.77	-2.93

Continuation of Table S5. Geoaccumulation index of heavy metal(loid)s.

	Cu		Pb		Zn		Cr		Ni		Cd		As		Hg	
	Shale	UCC	Shale	UCC	Shale	UCC	Shale	UCC	Shale	UCC	Shale	UCC	Shale	UCC	Shale	UCC*
LKD136	-2.70	-1.85	-2.65	-2.65	-5.21	-4.79	-2.07	-0.71	-4.15	-2.39	-5.57	-3.95	-4.02	0.26	-5.32	-2.49
LKD137	-2.07	-1.22	-2.24	-2.24	-4.53	-4.11	-3.96	-2.60	-4.20	-2.44	-6.07	-4.46	-3.48	0.81	-5.64	-2.81
LKD138	-2.79	-1.94	-4.07	-4.07	-12.21	-11.79	-2.09	-0.73	-5.03	-3.26	-4.35	-2.74	-3.82	0.46	-5.95	-3.11
LKD139	-3.20	-2.36	-3.40	-3.40	-6.00	-5.58	-3.40	-2.04	-5.50	-3.73	-4.35	-2.74	-4.23	0.06	-5.64	-2.81
LKD140	-3.09	-2.24	-3.82	-3.82	-5.57	-5.14	-3.31	-1.95	-5.76	-4.00	-5.87	-4.25	-3.63	0.65	-5.64	-2.81
LKD141	-3.02	-2.17	-3.55	-3.55	-7.26	-6.84	-3.54	-2.18	-5.40	-3.63	-5.01	-3.39	-3.12	1.17	-5.77	-2.93
LKD142	-7.23	-6.38	-2.73	-2.73	-6.90	-6.48	-5.14	-3.78	-10.73	-8.97	-8.05	-6.43	-5.05	-0.76	-5.77	-2.93
LKD143	-6.68	-5.83	-2.32	-2.32	-5.02	-4.60	-6.19	-4.83	-10.73	-8.97	-4.64	-3.03	-5.29	-1.00	-5.53	-2.69
LKD144	-9.02	-8.17	-2.28	-2.28	-5.39	-4.97	-8.59	-7.23	-10.73	-8.97	-7.81	-6.20	-4.63	-0.34	-5.77	-2.93
LKD145	-8.55	-7.70	-1.98	-1.98	-5.00	-4.58	-8.59	-7.23	-10.73	-8.97	-6.55	-4.94	-6.54	-2.25	-5.53	-2.69
LKD146	-2.47	-1.62	-8.97	-8.97	-12.21	-11.79	-2.05	-0.69	-4.95	-3.19	-7.89	-6.27	-3.24	1.05	-5.77	-2.93
LKD147	-3.73	-2.88	-4.37	-4.37	-12.21	-11.79	-7.59	-6.23	-5.75	-3.99	-7.81	-6.20	-4.33	-0.04	-5.64	-2.81
LKD148	-3.30	-2.45	-3.88	-3.88	-5.06	-4.64	-3.64	-2.28	-4.78	-3.01	-7.74	-6.13	-3.78	0.51	-5.32	-2.49
LKD149	-4.03	-3.19	-5.09	-5.09	-6.14	-5.72	-4.20	-2.84	-6.19	-4.42	-6.20	-4.59	-4.70	-0.42	-5.14	-2.30
LKD150	-3.52	-2.67	-4.71	-4.71	-12.21	-11.79	-3.25	-1.89	-5.29	-3.52	-7.61	-6.00	-4.37	-0.09	-5.42	-2.58
LKD151	-2.22	-1.37	-8.97	-8.97	-12.21	-11.79	-6.41	-5.05	-4.57	-2.80	-7.89	-6.27	-3.41	0.88	-5.14	-2.30
LKD152	-2.43	-1.58	-5.67	-5.67	-12.21	-11.79	-1.73	-0.36	-4.49	-2.72	-7.81	-6.20	-3.72	0.57	-5.42	-2.58
LKD153	-2.17	-1.32	-4.26	-4.26	-5.65	-5.23	-2.13	-0.77	-4.69	-2.92	-7.74	-6.13	-3.20	1.08	-5.32	-2.49
LKD154	-3.84	-3.00	-7.23	-7.23	-5.93	-5.51	-3.87	-2.51	-6.73	-4.97	-2.86	-1.25	-3.85	0.43	-5.64	-2.81
LKD155	-3.18	-2.33	-2.07	-2.07	-5.49	-5.07	-2.67	-1.31	-5.11	-3.34	-7.44	-5.82	-3.86	0.42	-5.64	-2.81
LKD156	-9.26	-8.41	-3.26	-3.26	-6.28	-5.86	-8.59	-7.23	-10.73	-8.97	-7.49	-5.88	-5.98	-1.69	-5.32	-2.49
LKD157	-6.59	-5.74	-2.55	-2.55	-5.15	-4.73	-5.93	-4.56	-7.79	-6.03	-6.09	-4.48	-5.64	-1.36	-5.53	-2.69
LKD158	-4.18	-3.33	-6.34	-6.34	-3.94	-3.52	-1.01	0.36	-4.25	-2.49	-7.28	-5.66	-5.80	-1.51	-5.14	-2.30
LKD159	-4.01	-3.16	-4.94	-4.94	-6.08	-5.66	-3.99	-2.63	-5.33	-3.56	-5.99	-4.37	-4.52	-0.23	-5.14	-2.30
LKD160	-2.71	-1.86	-2.98	-2.98	-3.08	-2.66	-3.31	-1.95	-3.25	-1.49	-7.74	-6.13	-3.50	0.78	-3.30	-0.46
LKD161	-3.33	-2.49	-4.34	-4.34	-3.96	-3.54	-0.94	0.43	-5.20	-3.43	-7.68	-6.06	-3.05	1.24	-4.58	-1.75
LKD162	-3.21	-2.36	-5.00	-5.00	-4.94	-4.52	-3.37	-2.00	-5.26	-3.50	-7.61	-6.00	-4.03	0.25	-5.06	-2.22

Continuation of Table S5. Geoaccumulation index of heavy metal(loid)s.

	Cu		Pb		Zn		Cr		Ni		Cd		As		Hg	
	Shale	UCC	Shale	UCC	Shale	UCC	Shale	UCC	Shale	UCC	Shale	UCC	Shale	UCC	Shale	UCC*
LKD163	-2.48	-1.63	-8.97	-8.97	-4.25	-3.83	-4.65	-3.28	-4.63	-2.86	-6.46	-4.85	-3.83	0.45	-5.32	-2.49
LKD164	-3.33	-2.48	-6.42	-6.42	-4.70	-4.28	-2.64	-1.27	-5.46	-3.69	-7.68	-6.06	-4.37	-0.09	-5.53	-2.69
LKD165	-1.58	-0.73	-3.32	-3.32	-4.14	-3.72	-1.49	-0.13	-3.61	-1.85	-2.57	-0.95	-2.74	1.54	-5.91	-3.07
LKD166	-2.99	-2.14	-5.51	-5.51	-12.37	-11.95	-2.75	-1.39	-4.92	-3.15	-7.81	-6.20	-3.74	0.55	-5.64	-2.81
LKD167	-3.44	-2.59	-8.97	-8.97	-12.21	-11.79	-3.14	-1.78	-5.85	-4.08	-7.74	-6.13	-3.02	1.27	-5.32	-2.49
LKD168	-2.74	-1.89	-6.85	-6.85	-5.89	-5.47	-2.46	-1.10	-4.41	-2.65	-7.61	-6.00	-3.91	0.38	-5.53	-2.69
LKD169	-7.33	-6.48	-5.06	-5.06	-5.96	-5.54	-8.59	-7.23	-7.99	-6.23	-7.74	-6.13	-5.07	-0.79	-5.42	-2.58
LKD170	-8.55	-7.70	-6.19	-6.19	-5.83	-5.41	-7.89	-6.53	-7.96	-6.19	-7.68	-6.06	-3.51	0.77	-5.32	-2.49
LKD171	-3.39	-2.55	-5.48	-5.48	-7.28	-6.86	-3.03	-1.67	-5.40	-3.64	-7.68	-6.06	-3.55	0.73	-5.53	-2.69
LKD172	-2.53	-1.68	-3.93	-3.93	-7.57	-7.15	-5.43	-4.07	-4.95	-3.18	-7.74	-6.13	-2.72	1.56	-4.71	-1.87
LKD173	-2.97	-2.12	-5.09	-5.09	-7.61	-7.19	-2.11	-0.75	-5.51	-3.74	-6.61	-5.00	-3.45	0.83	-5.14	-2.30
LKD174	-3.58	-2.73	-8.97	-8.97	-6.34	-5.92	-4.67	-3.31	-5.92	-4.16	-3.23	-1.61	-3.17	1.11	-5.23	-2.39
LKD175	-3.15	-2.30	-8.97	-8.97	-6.83	-6.41	-2.96	-1.60	-5.48	-3.71	-7.61	-6.00	-3.75	0.54	-5.53	-2.69
LKD176	-2.49	-1.64	-8.74	-8.74	-6.25	-5.83	-2.10	-0.74	-4.09	-2.32	-7.81	-6.20	-3.07	1.22	-5.23	-2.39
LKD177	-2.98	-2.13	-4.37	-4.37	-5.41	-4.99	-2.90	-1.54	-4.60	-2.84	-7.68	-6.06	-3.24	1.04	-5.64	-2.81
LKD178	-2.22	-1.37	-8.97	-8.97	-7.20	-6.78	-2.10	-0.74	-4.38	-2.62	-7.74	-6.13	-2.90	1.39	-5.23	-2.39
LKD179	-1.55	-0.70	-6.03	-6.03	-3.86	-3.44	-5.29	-3.93	-5.12	-3.36	-7.68	-6.06	-3.83	0.45	-5.42	-2.58
LKD180	-3.21	-2.36	-3.30	-3.30	-4.48	-4.06	-2.67	-1.31	-4.43	-2.66	-7.81	-6.20	-3.49	0.80	-5.53	-2.69
LKD181	-6.35	-5.50	-2.99	-2.99	-3.74	-3.32	-7.12	-5.76	-8.62	-6.85	-8.44	-6.82	-5.26	-0.97	-5.42	-2.58
LKD182	-9.14	-8.29	-3.10	-3.10	-6.32	-5.90	-5.89	-4.53	-7.56	-5.80	-3.29	-1.68	-6.34	-2.06	-5.32	-2.49
LKD183	-6.23	-5.38	-3.27	-3.27	-6.13	-5.71	-2.89	-1.53	-5.60	-3.83	-7.81	-6.20	-4.70	-0.42	-4.91	-2.07
LKD184	-7.63	-6.79	-3.42	-3.42	-3.83	-3.41	-6.05	-4.69	-6.12	-4.35	-7.68	-6.06	-5.20	-0.92	-5.06	-2.22
LKD185	-3.27	-2.42	-1.68	-1.68	-4.12	-3.70	-3.20	-1.84	-5.58	-3.82	-7.61	-6.00	-3.80	0.49	-5.64	-2.81
LKD186	-2.23	-1.38	-5.34	-5.34	-5.26	-4.84	-2.21	-0.85	-4.53	-2.77	-7.49	-5.88	-2.43	1.86	-4.53	-1.69
LKD187	-3.62	-2.77	-8.97	-8.97	-6.35	-5.93	-4.52	-3.15	-5.56	-3.80	-3.09	-1.47	-3.35	0.93	-5.42	-2.58
LKD188	-3.59	-2.75	-8.97	-8.97	-4.34	-3.92	-3.12	-1.76	-5.42	-3.65	-7.23	-5.61	-4.10	0.19	-5.64	-2.81
LKD189	-2.43	-1.58	-4.25	-4.25	-4.99	-4.57	-1.81	-0.44	-4.32	-2.55	-3.68	-2.07	-3.63	0.65	-6.01	-3.18

Continuation of Table S5. Geoaccumulation index of heavy metal(loid)s.

	Cu		Pb		Zn		Cr		Ni		Cd		As		Hg	
	Shale	UCC	Shale	UCC	Shale	UCC	Shale	UCC	Shale	UCC	Shale	UCC	Shale	UCC	Shale	UCC*
LKD190	-2.83	-1.98	-4.13	-4.13	-4.93	-4.51	-3.28	-1.92	-5.92	-4.16	-4.35	-2.74	-3.73	0.56	-5.64	-2.81
LKD191	-2.54	-1.69	-3.63	-3.63	-4.63	-4.21	-2.52	-1.16	-4.40	-2.63	-4.64	-3.03	-3.25	1.04	-6.03	-3.19
LKD192	-2.11	-1.27	-3.56	-3.56	-4.43	-4.01	-1.96	-0.60	-4.34	-2.57	-4.64	-3.03	-3.18	1.10	-5.91	-3.07
LKD193	-2.40	-1.55	-3.50	-3.50	-4.25	-3.83	-2.82	-1.46	-3.51	-1.75	-4.17	-2.56	-3.63	0.66	-5.64	-2.81
LKD194	-2.71	-1.86	-3.52	-3.52	-4.69	-4.27	-2.09	-0.73	-4.13	-2.37	-4.91	-3.29	-3.43	0.86	-5.64	-2.81
LKD195	-5.90	-5.05	-2.97	-2.97	-4.62	-4.20	-6.02	-4.66	-10.73	-8.97	-4.81	-3.20	-6.02	-1.74	-5.77	-2.93
LKD196	-6.08	-5.23	-3.10	-3.10	-4.64	-4.22	-5.94	-4.58	-10.73	-8.97	-4.29	-2.68	-6.17	-1.89	-5.64	-2.81
LKD197	-6.15	-5.30	-1.89	-1.89	-4.46	-4.04	-4.41	-3.05	-6.25	-4.48	-4.35	-2.74	-6.93	-2.64	-5.91	-3.07
LKD198	-5.88	-5.03	-2.12	-2.12	-4.40	-3.98	-3.73	-2.36	-6.07	-4.30	-4.23	-2.61	-6.34	-2.06	-5.91	-3.07
LKD199	-2.59	-1.74	-3.31	-3.31	-4.26	-3.84	-3.12	-1.76	-7.43	-5.67	-3.35	-1.74	-4.95	-0.67	-5.77	-2.93
LKD200	-2.03	-1.18	-3.67	-3.67	-4.71	-4.29	-3.89	-2.53	-8.23	-6.46	-4.42	-2.81	-3.77	0.52	-5.77	-2.93
LKD201	-3.03	-2.18	-3.68	-3.68	-4.40	-3.98	-2.47	-1.11	-4.03	-2.27	-4.29	-2.68	-4.34	-0.06	-5.64	-2.81
LKD202	-2.81	-1.96	-3.86	-3.86	-4.70	-4.28	-2.74	-1.38	-5.02	-3.25	-4.06	-2.44	-4.02	0.26	-5.91	-3.07
LKD203	-2.53	-1.68	-3.50	-3.50	-4.53	-4.11	-1.72	-0.35	-4.31	-2.55	-4.73	-3.11	-3.59	0.70	-5.77	-2.93
LKD204	-2.24	-1.39	-3.49	-3.49	-4.25	-3.83	-2.38	-1.02	-4.16	-2.40	-3.86	-2.25	-3.71	0.58	-5.91	-3.07
LKD205	-2.99	-2.14	-3.69	-3.69	-4.51	-4.09	-3.44	-2.08	-5.98	-4.21	-4.29	-2.68	-3.57	0.71	-5.64	-2.81
LKD206	-3.05	-2.20	-3.42	-3.42	-4.36	-3.94	-4.67	-3.31	-10.73	-8.97	-4.42	-2.81	-4.52	-0.23	-6.09	-3.25
LKD207	-4.40	-3.55	-3.34	-3.34	-5.06	-4.64	-4.56	-3.20	-10.73	-8.97	-4.23	-2.61	-4.61	-0.32	-5.97	-3.13
LKD208	-2.42	-1.57	-3.21	-3.21	-4.86	-4.44	-2.31	-0.95	-5.47	-3.71	-4.91	-3.29	-3.38	0.90	-5.53	-2.69
LKD209	-6.05	-5.20	-3.22	-3.22	-4.78	-4.36	-5.49	-4.13	-5.34	-3.57	-4.57	-2.95	-6.23	-1.94	-5.98	-3.14
LKD210	-5.32	-4.47	0.62	0.62	-3.30	-2.88	-2.50	-1.14	-3.83	-2.07	-4.11	-2.50	-6.47	-2.18	-5.98	-3.14
LKD211	-5.97	-5.12	-3.37	-3.37	-4.96	-4.54	-5.94	-4.58	-5.04	-3.28	-4.73	-3.11	-6.68	-2.40	-5.97	-3.13
LKD212	-5.86	-5.01	-4.09	-4.09	-5.04	-4.62	-4.06	-2.70	-10.73	-8.97	-4.35	-2.74	-7.47	-3.18	-5.42	-2.58
LKD213	-2.98	-2.13	-4.20	-4.20	-5.06	-4.64	-2.04	-0.68	-7.19	-5.42	-4.29	-2.68	-4.02	0.26	-5.95	-3.11
LKD214	-1.82	-0.97	-3.38	-3.38	-4.90	-4.48	-3.87	-2.51	-4.22	-2.45	-4.64	-3.03	-3.38	0.90	-6.08	-3.24
LKD215	-2.03	-1.18	-2.90	-2.90	-3.31	-2.89	-0.46	0.90	-3.12	-1.36	-3.91	-2.29	-3.12	1.17	-5.95	-3.11
LKD216	-2.76	-1.91	-3.07	-3.07	-4.97	-4.55	-3.00	-1.64	-5.33	-3.56	-5.49	-3.88	-4.16	0.12	-5.77	-2.93

Continuation of Table S5. Geoaccumulation index of heavy metal(loid)s.

	Cu		Pb		Zn		Cr		Ni		Cd		As		Hg	
	Shale	UCC	Shale	UCC	Shale	UCC	Shale	UCC	Shale	UCC	Shale	UCC	Shale	UCC	Shale	UCC*
LKD217	-3.57	-2.72	-2.63	-2.63	-4.44	-4.02	-2.11	-0.75	-4.82	-3.05	-4.49	-2.88	-3.70	0.58	-6.03	-3.19
LKD218	-2.02	-1.18	-3.55	-3.55	-4.23	-3.81	-1.72	-0.36	-3.92	-2.15	-4.42	-2.81	-3.24	1.05	-6.03	-3.19
LKD219	-2.52	-1.67	-3.04	-3.04	-4.34	-3.92	-2.22	-0.86	-5.62	-3.86	-5.11	-3.50	-3.62	0.67	-5.64	-2.81
LKD220	-2.50	-1.66	-3.12	-3.12	-4.45	-4.03	-3.10	-1.74	-6.04	-4.27	-4.81	-3.20	-3.56	0.72	-5.23	-2.39
LKD221	-3.31	-2.46	-3.68	-3.68	-5.12	-4.70	-2.52	-1.16	-10.73	-8.97	-4.81	-3.20	-3.74	0.55	-5.91	-3.07
LKD222	-2.23	-1.38	-2.87	-2.87	-4.60	-4.18	-1.88	-0.51	-4.46	-2.69	-4.91	-3.29	-3.26	1.02	-5.64	-2.81
LKD223	-5.95	-5.10	-3.62	-3.62	-5.00	-4.58	-3.95	-2.59	-5.60	-3.84	-4.49	-2.88	-5.76	-1.47	-6.00	-3.16
LKD224	-5.83	-4.98	-3.86	-3.86	-5.05	-4.63	-4.21	-2.85	-10.73	-8.97	-4.57	-2.95	-5.26	-0.97	-5.97	-3.13
LKD225	-5.57	-4.72	-3.64	-3.64	-4.61	-4.19	-3.73	-2.36	-3.99	-2.23	-4.42	-2.81	-6.84	-2.56	-5.91	-3.07
LKD226	-5.39	-4.54	-2.99	-2.99	-4.49	-4.07	-3.30	-1.94	-4.02	-2.25	-3.77	-2.16	-5.50	-1.22	-5.53	-2.69
LKD227	-3.60	-2.75	-2.44	-2.44	-4.51	-4.09	-2.10	-0.74	-4.63	-2.87	-4.06	-2.44	-4.17	0.11	-5.77	-2.93
LKD228	-2.08	-1.23	-2.10	-2.10	-2.96	-2.54	-0.84	0.52	-3.88	-2.11	-4.35	-2.74	-3.53	0.76	-6.28	-3.45
LKD229	-2.83	-1.98	-3.29	-3.29	-4.32	-3.90	-2.04	-0.68	-6.67	-4.91	-4.29	-2.68	-4.26	0.03	-5.53	-2.69
LKD230	-2.67	-1.82	-2.64	-2.64	-3.82	-3.40	-2.42	-1.05	-4.48	-2.71	-4.29	-2.68	-4.29	0.00	-5.42	-2.58
LKD231	-2.73	-1.88	-0.56	-0.56	-5.28	-4.86	-2.62	-1.25	-4.68	-2.91	-4.73	-3.11	-3.46	0.82	-6.23	-3.39
LKD232	-3.08	-2.23	-3.40	-3.40	-5.02	-4.60	-3.15	-1.79	-4.92	-3.15	-3.91	-2.29	-3.81	0.48	-6.23	-3.39
LKD233	-2.59	-1.74	-2.48	-2.48	-4.73	-4.31	-3.42	-2.06	-4.74	-2.98	-4.17	-2.56	-4.22	0.07	-6.40	-3.56
LKD234	-3.04	-2.19	-3.78	-3.78	-4.82	-4.40	-2.08	-0.71	-4.84	-3.08	-4.64	-3.03	-3.41	0.87	-5.97	-3.13
LKD235	-2.46	-1.61	-3.73	-3.73	-4.75	-4.33	-2.60	-1.24	-4.04	-2.27	-4.17	-2.56	-4.29	0.00	-5.98	-3.14
LKD236	-5.90	-5.05	-2.60	-2.60	-4.67	-4.25	-4.28	-2.92	-10.73	-8.97	-4.29	-2.68	-5.93	-1.64	-6.12	-3.29
LKD237	-6.26	-5.41	-3.52	-3.52	-4.76	-4.34	-3.96	-2.60	-5.55	-3.78	-4.29	-2.68	-6.76	-2.47	-5.64	-2.81
LKD238	-6.15	-5.30	-3.13	-3.13	-4.80	-4.38	-3.94	-2.58	-5.67	-3.90	-3.96	-2.34	-4.91	-0.62	-5.42	-2.58
LKD239	-5.42	-4.57	-2.54	-2.54	-4.37	-3.95	-5.24	-3.88	-10.73	-8.97	-4.35	-2.74	-6.17	-1.89	-5.91	-3.07
LKD240	-2.21	-1.36	-1.45	-1.45	-4.15	-3.73	-3.60	-2.24	-2.89	-1.12	-4.06	-2.44	-3.80	0.49	-5.91	-3.07
LKD241	-2.41	-1.56	-3.33	-3.33	-4.99	-4.57	-1.69	-0.33	-5.21	-3.45	-4.35	-2.74	-3.25	1.04	-6.06	-3.22
LKD242	-2.88	-2.03	-3.20	-3.20	-4.65	-4.23	-2.17	-0.81	-4.35	-2.58	-4.57	-2.95	-4.08	0.20	-5.32	-2.49
LKD243	-2.67	-1.82	-3.64	-3.64	-4.47	-4.05	-2.03	-0.67	-4.06	-2.29	-3.86	-2.25	-3.69	0.59	-5.91	-3.07

Continuation of Table S5. Geoaccumulation index of heavy metal(loid)s.

	Cu		Pb		Zn		Cr		Ni		Cd		As		Hg	
	Shale	UCC	Shale	UCC	Shale	UCC	Shale	UCC	Shale	UCC	Shale	UCC	Shale	UCC	Shale	UCC*
LKD244	-2.16	-1.31	-2.23	-2.23	-3.89	-3.47	-1.53	-0.17	-2.65	-0.88	-4.29	-2.68	-3.36	0.93	-5.42	-2.58
LKD245	-2.32	-1.47	-2.47	-2.47	-4.88	-4.46	-2.84	-1.48	-4.59	-2.82	-4.23	-2.61	-3.47	0.82	-5.53	-2.69
LKD246	-2.66	-1.81	-2.76	-2.76	-4.65	-4.23	-2.53	-1.17	-4.41	-2.64	-4.23	-2.61	-3.88	0.40	-5.42	-2.58
LKD247	-2.92	-2.07	-3.49	-3.49	-4.91	-4.49	-2.43	-1.07	-5.18	-3.41	-4.57	-2.95	-3.70	0.58	-5.94	-3.10
LKD248	-2.29	-1.44	-2.71	-2.71	-4.11	-3.69	-2.09	-0.73	-5.09	-3.33	-4.06	-2.44	-3.34	0.94	-5.77	-2.93
LKD249	-2.39	-1.54	-2.84	-2.84	-4.80	-4.38	-2.26	-0.90	-5.06	-3.29	-4.49	-2.88	-3.90	0.39	-5.91	-3.07
LKD250	-5.63	-4.79	-3.63	-3.63	-4.94	-4.52	-4.22	-2.86	-10.73	-8.97	-4.64	-3.03	-6.47	-2.18	-5.53	-2.69
LKD251	-5.77	-4.92	-3.27	-3.27	-4.83	-4.41	-4.22	-2.86	-7.73	-5.97	-3.91	-2.29	-0.64	3.64	2.26	5.09
LKD252	-6.35	-5.50	-3.84	-3.84	-4.89	-4.47	-6.74	-5.38	-10.73	-8.97	-4.42	-2.81	-5.44	-1.15	-6.08	-3.24
LKD253	-6.18	-5.33	-2.63	-2.63	-4.40	-3.98	-5.67	-4.30	-10.73	-8.97	-3.57	-1.95	-7.91	-3.63	-6.19	-3.36
LKD254	-1.99	-1.14	-3.02	-3.02	-4.23	-3.81	-3.39	-2.02	-3.94	-2.18	-3.91	-2.29	-3.55	0.73	-5.23	-2.39
LKD255	-2.13	-1.28	-2.17	-2.17	-4.30	-3.88	-1.10	0.26	-3.29	-1.53	-4.11	-2.50	-3.33	0.96	-5.42	-2.58
LKD256	-2.97	-2.12	-2.94	-2.94	-4.45	-4.03	-3.29	-1.93	-3.93	-2.17	-4.29	-2.68	-3.70	0.58	-5.77	-2.93
LKD257	-2.63	-1.78	-3.66	-3.66	-4.80	-4.38	-2.68	-1.32	-5.35	-3.58	-4.64	-3.03	-3.71	0.58	-5.77	-2.93
LKD258	-2.19	-1.34	-3.45	-3.45	-4.53	-4.11	-2.01	-0.65	-4.71	-2.94	-4.11	-2.50	-3.18	1.10	-5.77	-2.93
LKD259	-2.50	-1.66	-3.64	-3.64	-4.78	-4.36	-2.96	-1.60	-4.74	-2.97	-4.49	-2.88	-3.60	0.69	-5.77	-2.93
LKD260	-2.52	-1.67	-2.82	-2.82	-4.40	-3.98	-3.87	-2.51	-5.50	-3.73	-4.06	-2.44	-3.68	0.60	-5.77	-2.93
LKD261	-2.86	-2.01	-2.21	-2.21	-4.12	-3.70	-2.58	-1.22	-5.15	-3.39	-4.01	-2.39	-3.46	0.82	-5.32	-2.49
LKD262	-1.78	-0.93	-1.94	-1.94	-3.72	-3.30	-2.02	-0.66	-4.98	-3.22	-4.64	-3.03	-2.86	1.43	-5.53	-2.69
LKD263	-2.39	-1.54	-2.67	-2.67	-4.34	-3.92	-2.38	-1.02	-4.87	-3.10	-3.86	-2.25	-3.41	0.87	-5.14	-2.30
LKD264	-5.74	-4.90	-3.69	-3.69	-4.77	-4.35	-6.13	-4.77	-5.33	-3.56	-4.23	-2.61	-5.64	-1.36	-5.53	-2.69
LKD265	-5.27	-4.42	-2.83	-2.83	-4.52	-4.10	-5.33	-3.97	-10.73	-8.97	-4.29	-2.68	-5.84	-1.56	-5.53	-2.69
LKD266	-5.43	-4.58	-3.58	-3.58	-4.47	-4.05	-5.97	-4.60	-6.76	-5.00	-4.91	-3.29	-6.84	-2.56	-5.77	-2.93
LKD267	-5.73	-4.88	-2.79	-2.79	-4.50	-4.08	-5.93	-4.57	-6.51	-4.74	-4.35	-2.74	-6.84	-2.56	-5.91	-3.07
LKD268	-1.75	-0.91	-2.97	-2.97	-4.07	-3.65	-4.41	-3.05	-5.56	-3.80	-4.57	-2.95	-3.80	0.49	-5.53	-2.69
LKD269	-1.88	-1.04	-2.46	-2.46	-4.20	-3.78	-1.21	0.15	-3.71	-1.95	-4.01	-2.39	-3.26	1.02	-5.53	-2.69
LKD270	-2.42	-1.57	-1.57	-1.57	-3.78	-3.36	-1.84	-0.47	-4.41	-2.65	-4.42	-2.81	-3.31	0.97	-5.98	-3.14

Continuation of Table S5. Geoaccumulation index of heavy metal(loid)s.

	Cu		Pb		Zn		Cr		Ni		Cd		As		Hg	
	Shale	UCC	Shale	UCC	Shale	UCC	Shale	UCC	Shale	UCC	Shale	UCC	Shale	UCC	Shale	UCC*
LKD271	-2.67	-1.82	-0.45	-0.45	-3.98	-3.56	-2.21	-0.85	-4.63	-2.87	-4.42	-2.81	-3.57	0.71	-5.92	-3.08
LKD272	-2.75	-1.91	-1.87	-1.87	-4.33	-3.91	-2.79	-1.43	-5.50	-3.74	-3.86	-2.25	-3.49	0.80	-5.77	-2.93
LKD273	-2.09	-1.24	-1.56	-1.56	-4.27	-3.85	-1.86	-0.50	-4.46	-2.69	-4.57	-2.95	-3.29	0.99	-5.91	-3.07
LKD274	-5.72	-4.87	-2.58	-2.58	-4.12	-3.70	-5.78	-4.42	-6.39	-4.62	-4.29	-2.68	-6.76	-2.47	-5.91	-3.07
LKD275	-2.71	-1.86	-1.46	-1.46	-3.91	-3.49	-2.63	-1.27	-3.54	-1.78	-3.73	-2.11	-3.88	0.40	-5.77	-2.93
LKD276	-2.06	-1.21	-1.95	-1.95	-3.94	-3.52	-1.81	-0.44	-3.43	-1.67	-3.17	-1.56	-3.83	0.45	-5.53	-2.69
LKD277	-2.12	-1.27	-3.04	-3.04	-3.76	-3.34	-2.13	-0.77	-2.60	-0.84	-3.73	-2.11	-3.55	0.74	-5.92	-3.08
LKD278	-5.95	-5.10	-2.95	-2.95	-4.57	-4.15	-4.52	-3.15	-6.29	-4.53	-3.77	-2.16	-6.29	-2.00	-5.77	-2.93
LKD279	-5.57	-4.72	-2.41	-2.41	-4.34	-3.92	-4.32	-2.96	-10.73	-8.97	-3.64	-2.03	-6.76	-2.47	-5.91	-3.07
LKD280	-5.84	-4.99	-2.67	-2.67	-5.05	-4.63	-4.22	-2.85	-5.92	-4.16	-3.68	-2.07	-7.12	-2.84	-5.42	-2.58
LKD281	-5.91	-5.07	-2.17	-2.17	-4.52	-4.10	-4.39	-3.03	-3.39	-1.62	-3.77	-2.16	-6.54	-2.25	-6.09	-3.25
LKD282	-2.15	-1.30	-2.24	-2.24	-5.49	-5.07	-3.37	-2.00	-10.73	-8.97	-4.01	-2.39	-3.92	0.37	-5.91	-3.07
LKD283	-1.99	-1.14	-1.63	-1.63	-3.99	-3.57	-1.21	0.16	-2.80	-1.04	-3.64	-2.03	-3.83	0.45	-5.91	-3.07
LKD284	-3.20	-2.36	-1.93	-1.93	-4.09	-3.67	-2.91	-1.55	-10.73	-8.97	-4.57	-2.95	-4.55	-0.27	-6.27	-3.43
LKD285	-2.84	-1.99	-1.94	-1.94	-4.51	-4.09	-2.60	-1.24	-3.77	-2.00	-4.23	-2.61	-3.80	0.49	-5.53	-2.69
LKD286	-2.43	-1.58	-1.59	-1.59	-4.61	-4.19	-2.51	-1.15	-3.08	-1.31	-4.06	-2.44	-3.65	0.63	-5.64	-2.81
LKD287	-2.59	-1.74	-3.21	-3.21	-5.37	-4.95	-3.07	-1.71	-2.92	-1.15	-3.91	-2.29	-3.58	0.70	-5.77	-2.93
LKD288	-2.40	-1.55	-2.32	-2.32	-4.79	-4.37	-4.03	-2.66	-3.19	-1.42	-3.96	-2.34	-4.11	0.18	-6.00	-3.16
LKD289	-2.58	-1.73	-2.45	-2.45	-4.47	-4.05	-2.55	-1.18	-4.01	-2.25	-4.73	-3.11	-3.26	1.03	-5.77	-2.93
LKD290	-2.18	-1.33	-2.60	-2.60	-5.25	-4.83	-2.47	-1.11	-5.19	-3.43	-4.23	-2.61	-3.78	0.51	-5.91	-3.07
LKD291	-2.77	-1.92	-2.42	-2.42	-4.66	-4.24	-3.20	-1.84	-2.50	-0.74	-4.17	-2.56	-3.88	0.40	-5.77	-2.93
LKD292	-5.68	-4.83	-1.72	-1.72	-4.60	-4.18	-5.05	-3.69	-5.01	-3.25	-4.35	-2.74	-5.47	-1.18	-5.97	-3.13
LKD293	-5.62	-4.77	-2.55	-2.55	-4.99	-4.57	-5.51	-4.14	-5.40	-3.63	-5.01	-3.39	-4.72	-0.43	-5.77	-2.93
LKD294	-5.98	-5.13	-2.46	-2.46	-4.68	-4.26	-6.08	-4.71	-4.45	-2.68	-4.81	-3.20	-5.41	-1.12	-5.42	-2.58
LKD295	-5.47	-4.62	-1.50	-1.50	-4.15	-3.73	-5.37	-4.01	-2.34	-0.58	-4.49	-2.88	-5.37	-1.09	-5.77	-2.93
LKD296	-2.26	-1.41	-2.07	-2.07	-4.45	-4.03	-4.73	-3.37	-3.45	-1.69	-3.91	-2.29	-4.02	0.26	-5.64	-2.81
LKD297	-2.62	-1.77	-3.47	-3.47	-4.29	-3.87	-2.30	-0.94	-3.87	-2.10	-4.29	-2.68	-3.68	0.60	-6.00	-3.16

Continuation of Table S5. Geoaccumulation index of heavy metal(loid)s.

	Cu		Pb		Zn		Cr		Ni		Cd		As		Hg	
	Shale	UCC	Shale	UCC	Shale	UCC	Shale	UCC	Shale	UCC	Shale	UCC	Shale	UCC	Shale	UCC*
LKD298	-3.20	-2.36	-3.05	-3.05	-4.46	-4.04	-2.88	-1.52	-3.28	-1.51	-4.42	-2.81	-4.00	0.29	-6.09	-3.25
LKD299	-2.89	-2.04	-2.82	-2.82	-4.17	-3.75	-2.97	-1.61	-2.65	-0.88	-3.86	-2.25	-3.72	0.57	-6.00	-3.16
LKD300	-2.46	-1.61	-2.12	-2.12	-3.78	-3.36	-2.75	-1.39	-2.21	-0.45	-3.77	-2.16	-3.35	0.93	-5.42	-2.58
LKD301	-2.33	-1.48	-2.19	-2.19	-3.93	-3.51	-2.91	-1.54	-4.23	-2.46	-4.49	-2.88	-3.30	0.99	-5.95	-3.11
LKD302	-3.20	-2.35	-1.84	-1.84	-4.20	-3.78	-5.41	-4.05	-3.90	-2.14	-4.42	-2.81	-4.16	0.12	-5.77	-2.93
LKD303	-3.84	-3.00	-2.18	-2.18	-4.46	-4.04	-3.50	-2.14	-2.83	-1.07	-3.86	-2.25	-4.07	0.21	-5.64	-2.81
LKD304	-3.23	-2.38	-1.89	-1.89	-4.32	-3.90	-2.68	-1.32	-3.69	-1.93	-4.11	-2.50	-3.69	0.59	-5.42	-2.58
LKD305	-2.44	-1.60	-1.51	-1.51	-4.78	-4.36	-2.32	-0.96	-3.35	-1.59	-4.42	-2.81	-3.49	0.80	-5.97	-3.13
LKD306	-2.33	-1.48	-0.75	-0.75	-3.85	-3.43	-4.83	-3.47	-2.56	-0.79	-4.35	-2.74	-3.74	0.55	-5.91	-3.07
LKD307	-2.77	-1.92	-2.30	-2.30	-4.23	-3.81	-2.49	-1.12	-2.95	-1.18	-4.49	-2.88	-3.69	0.59	-5.32	-2.49
LKD308	-3.25	-2.40	-2.31	-2.31	-4.32	-3.90	-2.69	-1.33	-4.06	-2.30	-4.35	-2.74	-3.70	0.58	-5.42	-2.58
LKD309	-1.74	-0.89	-0.88	-0.88	-2.12	-1.70	-1.25	0.11	-1.38	0.38	-3.49	-1.88	-3.80	0.49	-3.06	-0.22

UCC: Upper continental crust value (2); Shale: Average shale values (3); UCC* (4).

Note: To maintain two digits after the decimal, values were rounded.

Table S6. Heavy metal(loid)s Er and RI.

	Cu	Pb	Cr	Ni	Zn	Cd	As	Hg	
	Er	Er	Er	Er	Er	Er	Er	Er	RI
LKD-1	3.12	1.65	0.37	1.04	0.06	12.24	13.40	12.14	44.99
LKD-2	4.80	1.29	1.25	0.74	0.14	10.41	11.07	10.00	40.33
LKD-3	2.50	0.85	1.48	0.81	0.03	9.18	11.20	10.71	37.44
LKD-4	0.10	3.48	0.09	0.02	0.04	12.24	3.93	10.00	30.04
LKD-5	5.96	1.10	1.53	0.67	0.18	11.33	10.27	11.43	43.07
LKD-6	0.12	0.44	0.30	0.02	0.03	12.86	6.80	10.00	30.68
LKD-7	0.12	0.62	0.09	0.15	0.02	10.10	7.20	8.57	27.24
LKD-8	0.18	2.85	0.11	0.02	0.09	11.33	3.00	7.14	24.98
LKD-9	4.32	0.30	0.70	0.97	0.09	10.10	16.20	7.00	40.41
LKD-10	0.12	0.46	0.09	0.13	0.02	10.41	8.27	10.00	29.81
LKD-11	6.14	1.35	1.22	0.85	0.20	11.33	9.67	11.43	43.06
LKD-12	5.72	5.25	1.02	0.71	0.20	11.02	8.87	10.71	44.10
LKD-13	2.24	0.60	0.54	0.73	0.02	12.86	12.80	10.71	41.19
LKD-14	1.58	0.41	0.50	0.58	0.02	10.71	9.00	10.00	33.45
LKD-15	0.24	1.16	0.13	0.01	0.01	12.55	3.93	10.71	28.90
LKD-16	0.02	0.79	0.18	0.02	0.00	12.86	6.73	12.14	32.86
LKD-17	0.28	0.51	0.33	0.23	0.02	12.24	4.67	10.71	29.18
LKD-18	0.18	0.87	0.46	0.45	0.05	13.78	6.20	10.00	32.43
LKD-19	0.16	0.81	0.17	0.02	0.02	14.39	6.60	12.86	35.15
LKD-20	0.15	0.35	0.10	0.02	0.01	12.55	8.00	10.71	32.01
LKD-21	0.29	0.89	0.36	0.34	0.06	13.47	7.20	12.86	35.79
LKD-22	0.09	0.20	0.25	0.08	0.01	13.47	7.93	12.14	34.29
LKD-23	2.36	0.17	1.71	1.14	0.02	10.41	15.07	9.29	41.11
LKD-24	2.06	0.93	1.78	1.01	0.05	14.39	11.13	9.29	41.63
LKD-25	5.80	0.86	0.89	0.87	0.17	12.24	10.80	10.00	42.33
LKD-26	5.10	2.75	0.39	0.84	0.15	13.47	7.93	10.00	41.43
LKD-27	9.10	1.05	1.53	1.15	0.33	12.55	13.07	15.71	55.68
LKD-28	6.64	0.06	2.26	1.77	0.14	11.94	19.73	10.00	53.51
LKD-29	4.18	0.57	4.90	1.27	0.08	11.02	19.20	15.00	57.07
LKD-30	3.30	0.59	0.63	1.34	0.02	11.94	13.87	8.57	41.32
LKD-31	2.38	1.17	1.59	1.90	0.08	13.47	9.47	9.29	40.78
LKD-32	2.62	4.78	1.54	1.02	0.03	14.39	10.73	9.29	45.22
LKD-33	3.34	1.23	2.06	1.53	0.03	15.61	10.80	8.57	44.16
LKD-34	2.50	27.00	2.38	1.93	0.03	11.94	11.93	8.57	67.42
LKD-35	1.21	0.91	0.67	0.92	0.02	10.10	9.33	7.86	31.80
LKD-36	2.20	1.18	0.87	1.88	0.08	11.63	10.87	10.00	40.18
LKD-37	3.04	1.13	3.97	1.37	0.09	14.39	14.93	12.86	52.77
LKD-38	2.28	0.95	1.31	1.43	0.03	15.00	12.27	9.29	43.51
LKD-39	4.54	0.48	0.54	1.63	0.02	12.86	16.80	9.29	47.39
LKD-40	3.60	0.55	3.68	1.57	0.07	12.24	13.60	10.71	47.20
LKD-41	2.36	0.98	1.35	1.00	0.07	10.71	8.33	8.57	34.13
LKD-42	1.98	1.18	1.13	0.83	0.07	12.24	13.07	10.00	41.26
LKD-43	0.17	0.18	0.57	0.12	0.13	9.18	3.13	7.86	21.44
LKD-44	0.36	0.50	0.37	0.34	0.04	14.39	5.73	10.00	32.00

Continuation of Table S6. Heavy metal(loid)s Er and RI.

	Cu	Pb	Cr	Ni	Zn	Cd	As	Hg	
	Er	Er	Er	Er	Er	Er	Er	Er	RI
LKD-45	0.23	0.15	0.22	0.13	0.03	10.10	4.33	10.71	26.07
LKD-46	0.11	0.58	0.18	0.18	0.05	12.55	4.13	7.86	25.88
LKD-47	1.84	0.61	0.56	1.07	0.10	11.63	10.40	11.43	38.50
LKD-48	2.72	1.06	2.54	1.32	0.10	8.57	12.00	12.14	41.70
LKD-49	1.85	5.10	1.48	0.97	0.04	9.80	13.33	7.86	41.30
LKD-50	2.68	15.33	1.82	1.05	0.04	13.47	13.80	7.14	56.30
LKD-51	2.40	0.62	1.18	1.22	0.02	11.94	13.13	8.57	39.95
LKD-52	3.60	0.21	0.45	1.34	0.03	8.88	13.67	9.29	38.62
LKD-53	1.81	2.09	1.19	0.74	0.05	13.47	7.80	9.29	37.25
LKD-54	2.52	0.53	1.46	1.05	0.07	10.41	6.20	10.71	33.81
LKD-55	2.50	0.60	1.59	0.92	0.07	12.24	6.07	8.57	33.44
LKD-56	1.60	1.17	1.25	0.71	0.05	13.16	5.53	7.86	32.07
LKD-57	0.13	0.44	0.20	0.16	0.05	12.86	6.67	7.86	28.52
LKD-58	0.33	0.47	0.32	0.39	0.05	10.71	1.27	9.29	23.04
LKD-59	0.17	0.51	0.13	0.17	0.08	11.94	2.40	9.29	24.85
LKD-60	0.27	0.50	0.15	0.20	0.07	8.57	0.57	8.57	19.10
LKD-61	4.42	0.26	2.74	1.70	0.04	15.61	12.20	7.14	45.21
LKD-62	4.36	0.37	0.60	1.15	0.08	12.86	10.27	7.14	37.72
LKD-63	4.86	1.17	2.15	1.72	0.08	11.63	16.27	9.29	48.28
LKD-64	2.28	2.50	1.70	0.94	0.04	11.63	12.67	10.00	42.75
LKD-65	4.70	0.82	1.80	0.94	0.11	12.24	9.53	20.00	51.16
LKD-66	4.46	0.46	0.58	1.63	0.01	12.24	13.93	8.57	43.18
LKD-67	3.38	0.48	2.99	1.31	0.02	11.94	10.87	7.14	39.07
LKD-68	2.92	0.12	1.59	1.18	0.03	13.47	11.93	7.14	39.34
LKD-69	0.26	0.26	1.81	1.17	0.02	12.55	2.73	10.00	29.61
LKD-70	1.52	0.17	0.78	0.69	0.04	9.80	9.80	7.86	31.37
LKD-71	0.39	0.32	1.82	1.05	0.02	10.10	2.73	10.00	27.20
LKD-72	0.16	0.14	0.17	0.16	0.02	9.80	1.00	7.14	18.73
LKD-73	0.39	1.14	0.21	0.59	0.11	10.71	11.27	16.43	41.64
LKD-74	0.24	0.68	0.27	0.23	0.04	12.24	2.47	10.00	26.58
LKD-75	0.20	0.30	0.32	0.24	0.03	10.41	3.73	8.57	24.06
LKD-76	0.11	0.28	0.15	0.12	0.02	8.88	2.40	8.57	20.65
LKD-77	3.96	1.06	1.18	0.95	0.12	11.63	6.67	7.86	34.17
LKD-78	5.82	1.49	0.53	0.97	0.21	12.55	7.67	10.00	40.17
LKD-79	1.69	1.72	0.66	0.82	0.03	9.80	7.33	7.86	30.52
LKD-80	1.44	0.81	1.01	0.45	0.02	15.00	5.67	7.14	32.12
LKD-81	1.33	0.14	0.58	0.54	0.03	9.80	7.53	7.00	27.64
LKD-82	2.92	0.02	0.37	1.02	0.02	11.02	6.40	7.86	30.49
LKD-83	1.93	0.34	1.80	0.92	0.04	9.49	5.40	8.57	29.16
LKD-84	2.46	0.27	1.34	0.93	0.05	8.27	6.73	7.86	28.58
LKD-85	1.34	1.37	0.82	0.58	0.01	9.49	3.87	7.00	25.06
LKD-86	3.50	0.53	1.78	1.18	0.20	11.33	11.93	7.86	39.43
LKD-87	0.39	0.65	0.44	0.64	0.05	9.80	0.73	10.71	23.65
LKD-88	0.67	0.89	0.93	0.67	0.11	11.94	1.00	8.57	25.23

Continuation of Table S6. Heavy metal(loid)s Er and RI.

	Cu	Pb	Cr	Ni	Zn	Cd	As	Hg	
	Er	Er	Er	Er	Er	Er	Er	Er	RI
LKD-89	0.04	0.45	0.18	0.08	0.07	7.04	2.40	7.14	17.56
LKD-90	0.33	0.25	0.25	0.02	0.05	11.33	0.22	6.57	19.20
LKD-91	2.96	0.93	1.14	13.45	0.07	12.24	6.53	9.29	47.21
LKD-92	2.36	0.89	0.39	12.43	0.02	12.24	7.93	8.57	45.43
LKD-93	1.58	0.43	0.55	13.30	0.03	11.02	6.33	8.57	42.50
LKD-94	1.15	0.53	0.77	23.15	0.03	10.10	7.00	7.86	51.11
LKD-95	1.64	0.44	0.55	13.03	0.04	11.33	4.53	8.57	40.83
LKD-96	3.78	0.35	0.52	15.35	0.03	9.49	9.47	7.14	47.25
LKD-97	1.90	0.40	1.77	0.92	0.00	2.45	7.07	9.29	24.63
LKD-98	1.42	0.52	0.89	0.41	0.01	1.07	9.00	11.43	25.26
LKD-99	1.80	0.41	1.65	0.50	0.00	0.64	7.80	8.57	22.09
LKD-100	1.67	0.76	0.90	0.51	0.00	6.12	6.13	10.00	26.82
LKD-101	0.11	0.67	0.18	0.02	0.00	0.61	4.27	9.29	15.27
LKD-102	0.09	2.29	0.17	0.02	0.01	0.64	1.33	11.43	16.42
LKD-103	0.41	0.48	0.15	0.02	0.00	0.67	1.20	10.71	13.72
LKD-104	1.45	1.53	0.70	0.23	0.00	4.59	5.33	10.00	24.28
LKD-105	2.42	1.83	0.35	0.65	0.02	4.29	9.07	11.43	30.74
LKD-106	1.91	0.48	0.56	3.70	0.00	9.49	8.07	9.29	34.17
LKD-107	0.88	0.60	0.81	0.68	0.00	2.05	8.07	10.00	23.62
LKD-108	1.90	0.39	0.88	1.71	0.02	0.64	8.53	9.29	24.18
LKD-109	2.38	0.46	0.24	1.05	0.02	5.51	9.40	10.00	29.80
LKD-110	2.24	0.64	1.69	0.83	0.10	7.04	7.60	7.86	28.64
LKD-111	1.34	0.89	0.75	0.51	0.00	1.50	5.53	10.00	21.09
LKD-112	6.62	0.80	1.04	0.45	0.20	0.70	9.13	8.57	28.10
LKD-113	1.82	0.77	0.93	0.51	0.00	6.43	8.13	8.57	27.87
LKD-114	1.30	0.52	0.74	0.44	0.01	1.96	4.00	10.00	19.57
LKD-115	0.15	2.32	0.26	1.76	0.02	0.92	1.80	10.71	18.17
LKD-116	0.15	1.84	0.11	0.44	0.00	7.35	3.60	10.71	24.32
LKD-117	0.22	2.44	0.24	0.15	0.04	3.00	1.00	10.00	17.37
LKD-118	1.71	1.58	0.89	0.63	0.05	1.84	7.27	11.43	26.11
LKD-119	1.32	0.59	0.35	0.34	0.00	6.73	8.40	10.00	28.14
LKD-120	1.98	1.38	1.47	0.93	0.11	3.98	11.80	11.43	33.72
LKD-121	1.29	0.49	0.94	0.77	0.00	3.98	9.20	10.71	27.92
LKD-122	1.19	0.41	0.66	0.63	0.00	8.88	7.20	9.29	28.83
LKD-123	2.60	0.55	0.25	0.87	0.00	7.04	10.87	7.86	30.88
LKD-124	1.89	0.46	1.61	0.45	0.00	6.12	10.13	10.00	31.20
LKD-125	1.43	0.85	0.67	0.53	0.00	7.96	8.67	12.14	32.86
LKD-126	1.70	0.59	1.14	0.38	0.00	5.51	11.93	8.57	30.40
LKD-127	2.28	0.41	1.46	0.72	0.02	0.77	3.20	9.29	19.03
LKD-128	0.12	1.06	0.11	0.01	0.00	3.98	12.20	10.71	28.33
LKD-129	0.16	1.43	0.04	0.01	0.00	9.80	4.80	8.57	24.90
LKD-130	0.29	2.35	0.29	0.52	0.06	0.58	0.87	9.29	14.80
LKD-131	0.01	2.63	0.15	0.19	0.05	0.55	3.60	10.71	17.95

Continuation of Table S6. Heavy metal(loid)s Er and RI.

	Cu	Pb	Cr	Ni	Zn	Cd	As	Hg	
	Er	Er	Er	Er	Er	Er	Er	Er	RI
LKD-132	1.77	0.76	1.07	0.63	0.04	3.37	8.40	10.00	26.59
LKD-133	2.10	0.80	0.56	0.88	0.04	3.37	9.73	8.57	26.87
LKD-134	1.78	0.92	1.37	0.81	0.03	0.58	11.13	10.00	27.23
LKD-135	2.16	1.11	1.33	1.24	0.02	10.71	10.47	7.86	35.70
LKD-136	2.08	1.19	1.84	1.43	0.05	2.91	8.00	10.71	29.12
LKD-137	3.22	1.59	0.50	1.39	0.09	2.05	11.67	8.57	30.24
LKD-138	1.96	0.45	1.81	0.78	0.00	6.73	9.20	6.93	28.59
LKD-139	1.46	0.71	0.73	0.57	0.03	6.73	6.93	8.57	26.40
LKD-140	1.59	0.53	0.78	0.47	0.04	2.36	10.47	8.57	25.43
LKD-141	1.66	0.64	0.66	0.61	0.01	4.29	15.00	7.86	31.58
LKD-142	0.09	1.13	0.22	0.02	0.02	0.52	3.93	7.86	13.90
LKD-143	0.13	1.50	0.11	0.02	0.06	5.51	3.33	9.29	20.03
LKD-144	0.03	1.55	0.02	0.02	0.05	0.61	5.27	7.86	15.52
LKD-145	0.04	1.90	0.02	0.02	0.06	1.47	1.40	9.29	14.80
LKD-146	2.44	0.02	1.86	0.82	0.00	0.58	13.80	7.86	28.34
LKD-147	1.02	0.36	0.04	0.47	0.00	0.61	6.47	8.57	18.09
LKD-148	1.37	0.51	0.62	0.93	0.06	0.64	9.47	10.71	25.08
LKD-149	0.82	0.22	0.42	0.35	0.03	1.87	5.00	12.14	21.56
LKD-150	1.18	0.29	0.81	0.65	0.00	0.70	6.27	10.00	20.68
LKD-151	2.90	0.02	0.09	1.08	0.00	0.58	12.27	12.14	30.13
LKD-152	2.50	0.15	2.33	1.14	0.00	0.61	9.87	10.00	27.63
LKD-153	3.00	0.39	1.76	0.99	0.04	0.64	14.13	10.71	32.66
LKD-154	0.94	0.05	0.53	0.24	0.03	18.98	9.00	8.57	38.90
LKD-155	1.49	1.78	1.21	0.74	0.04	0.80	8.93	8.57	24.59
LKD-156	0.02	0.78	0.02	0.02	0.03	0.77	2.07	10.71	14.51
LKD-157	0.14	1.28	0.13	0.12	0.06	2.02	2.60	9.29	15.84
LKD-158	0.75	0.09	3.84	1.34	0.13	0.89	2.33	12.14	22.87
LKD-159	0.84	0.25	0.49	0.64	0.03	2.17	5.67	12.14	22.90
LKD-160	2.06	0.95	0.78	2.68	0.24	0.64	11.47	43.57	63.96
LKD-161	1.34	0.37	4.03	0.70	0.13	0.67	15.73	17.86	41.56
LKD-162	1.46	0.24	0.75	0.67	0.07	0.70	7.93	12.86	25.59
LKD-163	2.42	0.02	0.31	1.03	0.11	1.56	9.13	10.71	26.28
LKD-164	1.35	0.09	1.24	0.58	0.08	0.67	6.27	9.29	20.13
LKD-165	4.52	0.75	2.75	2.09	0.11	23.27	19.40	7.14	61.87
LKD-166	1.70	0.17	1.14	0.85	0.00	0.61	9.73	8.57	23.69
LKD-167	1.24	0.02	0.87	0.44	0.00	0.64	16.07	10.71	30.86
LKD-168	2.02	0.07	1.40	1.20	0.03	0.70	8.67	9.29	25.58
LKD-169	0.08	0.23	0.02	0.10	0.03	0.64	3.87	10.00	15.45
LKD-170	0.04	0.10	0.03	0.10	0.04	0.67	11.40	10.71	23.45
LKD-171	1.28	0.17	0.94	0.60	0.01	0.67	11.07	9.29	24.86
LKD-172	2.34	0.49	0.18	0.83	0.01	0.64	19.67	16.43	41.52
LKD-173	1.73	0.22	1.78	0.56	0.01	1.41	11.87	12.14	30.39
LKD-174	1.13	0.02	0.30	0.42	0.02	14.69	14.40	11.43	42.98
LKD-175	1.52	0.02	0.99	0.57	0.02	0.70	9.67	9.29	23.56

Continuation of Table S6. Heavy metal(loid)s Er and RI.

	Cu	Pb	Cr	Ni	Zn	Cd	As	Hg	
	Er	Er	Er	Er	Er	Er	Er	Er	RI
LKD-176	2.40	0.02	1.80	1.50	0.03	0.61	15.53	11.43	34.39
LKD-177	1.72	0.36	1.03	1.05	0.05	0.67	13.73	8.57	28.27
LKD-178	2.90	0.02	1.79	1.22	0.01	0.64	17.47	11.43	36.59
LKD-179	4.62	0.12	0.20	0.73	0.14	0.67	9.13	10.00	26.67
LKD-180	1.46	0.76	1.21	1.19	0.09	0.61	11.60	9.29	27.45
LKD-181	0.17	0.94	0.06	0.07	0.15	0.40	3.40	10.00	15.59
LKD-182	0.02	0.88	0.13	0.14	0.03	14.08	1.60	10.71	27.85
LKD-183	0.18	0.78	1.04	0.53	0.03	0.61	5.00	14.29	22.81
LKD-184	0.07	0.70	0.12	0.37	0.14	0.67	3.53	12.86	18.94
LKD-185	1.40	2.34	0.84	0.53	0.12	0.70	9.33	8.57	24.53
LKD-186	2.88	0.19	1.66	1.10	0.05	0.77	24.20	18.57	50.53
LKD-187	1.10	0.02	0.34	0.54	0.02	16.22	12.73	10.00	41.58
LKD-188	1.12	0.02	0.89	0.60	0.10	0.92	7.60	8.57	20.39
LKD-189	2.50	0.40	2.21	1.28	0.06	10.71	10.47	6.64	35.14
LKD-190	1.90	0.43	0.79	0.42	0.07	6.73	9.80	8.57	29.64
LKD-191	2.32	0.61	1.34	1.21	0.08	5.51	13.67	6.57	32.35
LKD-192	3.12	0.64	1.98	1.26	0.09	5.51	14.33	7.14	35.27
LKD-193	2.56	0.66	1.09	2.24	0.11	7.65	10.53	8.57	34.53
LKD-194	2.06	0.65	1.81	1.46	0.08	4.59	12.07	8.57	32.12
LKD-195	0.23	0.96	0.12	0.02	0.08	4.90	2.00	7.86	16.42
LKD-196	0.20	0.88	0.13	0.02	0.08	7.04	1.80	8.57	18.89
LKD-197	0.19	2.02	0.36	0.34	0.09	6.73	1.07	7.14	18.28
LKD-198	0.23	1.73	0.58	0.38	0.09	7.35	1.60	7.14	19.38
LKD-199	2.24	0.76	0.89	0.15	0.10	13.47	4.20	7.86	30.21
LKD-200	3.30	0.59	0.52	0.09	0.08	6.43	9.53	7.86	29.44
LKD-201	1.66	0.59	1.39	1.56	0.10	7.04	6.40	8.57	27.94
LKD-202	1.93	0.52	1.15	0.79	0.08	8.27	8.00	7.14	28.64
LKD-203	2.34	0.66	2.35	1.28	0.09	5.20	10.80	7.86	31.40
LKD-204	2.86	0.67	1.48	1.43	0.11	9.49	9.93	7.14	34.45
LKD-205	1.70	0.58	0.71	0.41	0.09	7.04	10.93	8.57	30.80
LKD-206	1.63	0.70	0.30	0.02	0.10	6.43	5.67	6.29	21.80
LKD-207	0.64	0.74	0.33	0.02	0.06	7.35	5.33	6.86	21.72
LKD-208	2.52	0.81	1.55	0.58	0.07	4.59	12.47	9.29	32.67
LKD-209	0.20	0.81	0.17	0.63	0.07	5.82	1.73	6.79	16.40
LKD-210	0.34	11.53	1.36	1.79	0.20	7.96	1.47	6.79	32.01
LKD-211	0.22	0.73	0.13	0.77	0.06	5.20	1.27	6.86	15.37
LKD-212	0.23	0.44	0.46	0.02	0.06	6.73	0.73	10.00	18.89
LKD-213	1.71	0.41	1.87	0.18	0.06	7.04	8.00	6.93	27.03
LKD-214	3.82	0.72	0.53	1.37	0.07	5.51	12.47	6.36	31.98
LKD-215	3.30	1.01	5.61	2.93	0.20	9.18	15.00	6.93	45.09
LKD-216	2.00	0.90	0.97	0.64	0.06	3.06	7.27	7.86	23.54
LKD-217	1.14	1.21	1.79	0.90	0.09	6.12	10.00	6.57	28.59
LKD-218	3.32	0.64	2.34	1.69	0.11	6.43	13.80	6.57	36.68
LKD-219	2.36	0.92	1.65	0.52	0.10	3.98	10.60	8.57	29.71

Continuation of Table S6. Heavy metal(loid)s Er and RI.

	Cu	Pb	Cr	Ni	Zn	Cd	As	Hg	
	Er	Er	Er	Er	Er	Er	Er	Er	RI
LKD-220	2.38	0.87	0.90	0.39	0.09	4.90	11.00	11.43	32.86
LKD-221	1.36	0.59	1.34	0.02	0.06	4.90	9.73	7.14	25.83
LKD-222	2.88	1.02	2.10	1.16	0.08	4.59	13.53	8.57	35.12
LKD-223	0.22	0.61	0.50	0.53	0.06	6.12	2.40	6.71	17.70
LKD-224	0.24	0.52	0.42	0.02	0.06	5.82	3.40	6.86	17.45
LKD-225	0.28	0.60	0.58	1.60	0.08	6.43	1.13	7.14	18.17
LKD-226	0.32	0.95	0.78	1.57	0.09	10.10	2.87	9.29	26.50
LKD-227	1.12	1.39	1.79	1.03	0.09	8.27	7.20	7.86	29.44
LKD-228	3.20	1.76	4.31	1.74	0.26	6.73	11.27	5.50	36.37
LKD-229	1.90	0.77	1.87	0.25	0.10	7.04	6.80	9.29	28.76
LKD-230	2.12	1.21	1.45	1.14	0.14	7.04	6.67	10.00	30.85
LKD-231	2.04	5.10	1.26	1.00	0.05	5.20	11.80	5.71	33.11
LKD-232	1.60	0.71	0.87	0.84	0.06	9.18	9.27	5.71	28.93
LKD-233	2.24	1.34	0.72	0.95	0.08	7.65	7.00	5.07	26.03
LKD-234	1.64	0.55	1.83	0.89	0.07	5.51	12.20	6.86	30.30
LKD-235	2.46	0.57	1.27	1.55	0.07	7.65	6.67	6.79	27.88
LKD-236	0.23	1.24	0.40	0.02	0.08	7.04	2.13	6.14	17.47
LKD-237	0.18	0.65	0.49	0.55	0.07	7.04	1.20	8.57	19.00
LKD-238	0.19	0.86	0.50	0.50	0.07	8.88	4.33	10.00	25.46
LKD-239	0.32	1.29	0.20	0.02	0.10	6.73	1.80	7.14	17.89
LKD-240	2.92	2.75	0.63	3.45	0.11	8.27	9.33	7.14	35.85
LKD-241	2.54	0.75	2.39	0.69	0.06	6.73	13.67	6.43	34.10
LKD-242	1.83	0.82	1.71	1.25	0.08	5.82	7.67	10.71	30.95
LKD-243	2.12	0.60	1.89	1.53	0.09	9.49	10.07	7.14	33.98
LKD-244	3.02	1.60	2.66	4.08	0.14	7.04	12.67	10.00	42.82
LKD-245	2.70	1.36	1.07	1.06	0.07	7.35	11.73	9.29	35.66
LKD-246	2.14	1.11	1.34	1.20	0.08	7.35	8.80	10.00	32.86
LKD-247	1.79	0.67	1.43	0.71	0.07	5.82	10.00	7.00	28.43
LKD-248	2.76	1.15	1.81	0.75	0.12	8.27	12.80	7.86	36.48
LKD-249	2.58	1.05	1.61	0.77	0.07	6.12	8.73	7.14	28.89
LKD-250	0.27	0.61	0.41	0.02	0.07	5.51	1.47	9.29	17.87
LKD-251	0.25	0.78	0.41	0.12	0.07	9.18	83.33	2050.00	2144.54
LKD-252	0.17	0.52	0.07	0.02	0.07	6.43	3.00	6.36	16.70
LKD-253	0.19	1.21	0.15	0.02	0.09	11.63	0.54	5.86	19.92
LKD-254	3.40	0.93	0.74	1.66	0.11	9.18	11.07	11.43	39.69
LKD-255	3.08	1.67	3.59	2.60	0.10	7.96	12.93	10.00	43.39
LKD-256	1.73	0.98	0.79	1.67	0.09	7.04	10.00	7.86	31.03
LKD-257	2.18	0.60	1.20	0.63	0.07	5.51	9.93	7.86	28.98
LKD-258	2.96	0.69	1.91	0.98	0.09	7.96	14.33	7.86	38.01
LKD-259	2.38	0.60	0.99	0.96	0.07	6.12	10.73	7.86	30.66
LKD-260	2.36	1.06	0.53	0.57	0.10	8.27	10.13	7.86	31.76
LKD-261	1.86	1.63	1.29	0.72	0.12	8.57	11.80	10.71	37.70
LKD-262	3.94	1.95	1.90	0.81	0.15	5.51	17.93	9.29	42.70
LKD-263	2.58	1.18	1.48	0.88	0.10	9.49	12.20	12.14	41.19

Continuation of Table S6. Heavy metal(loid)s Er and RI.

	Cu	Pb	Cr	Ni	Zn	Cd	As	Hg	
	Er	Er	Er	Er	Er	Er	Er	Er	RI
LKD-264	0.25	0.58	0.11	0.64	0.07	7.35	2.60	9.29	21.05
LKD-265	0.35	1.05	0.19	0.02	0.09	7.04	2.27	9.29	20.55
LKD-266	0.31	0.63	0.12	0.24	0.09	4.59	1.13	7.86	15.16
LKD-267	0.25	1.09	0.13	0.28	0.09	6.73	1.13	7.14	17.27
LKD-268	4.00	0.96	0.36	0.54	0.12	5.82	9.33	9.29	31.46
LKD-269	3.66	1.36	3.33	1.95	0.11	8.57	13.53	9.29	43.01
LKD-270	2.52	2.53	2.16	1.20	0.15	6.43	13.07	6.79	36.10
LKD-271	2.12	5.48	1.67	1.03	0.13	6.43	10.93	7.07	35.80
LKD-272	2.00	2.05	1.11	0.56	0.10	9.49	11.60	7.86	35.65
LKD-273	3.18	2.55	2.13	1.16	0.10	5.82	13.27	7.14	36.44
LKD-274	0.26	1.25	0.14	0.31	0.12	7.04	1.20	7.14	17.95
LKD-275	2.06	2.73	1.25	2.19	0.13	10.41	8.80	7.86	36.50
LKD-276	3.24	1.94	2.21	2.37	0.13	15.31	9.13	9.29	44.60
LKD-277	3.10	0.91	1.76	4.20	0.15	10.41	11.13	7.07	39.75
LKD-278	0.22	0.97	0.34	0.33	0.08	10.10	1.67	7.86	21.70
LKD-279	0.28	1.42	0.39	0.02	0.10	11.02	1.20	7.14	21.81
LKD-280	0.24	1.18	0.41	0.42	0.06	10.71	0.93	10.00	24.06
LKD-281	0.22	1.67	0.37	2.44	0.09	10.10	1.40	6.29	22.74
LKD-282	3.04	1.58	0.75	0.02	0.04	8.57	8.60	7.14	30.60
LKD-283	3.40	2.42	3.34	3.65	0.13	11.02	9.13	7.14	41.11
LKD-284	1.46	1.97	1.02	0.02	0.12	5.82	5.53	5.57	22.13
LKD-285	1.89	1.96	1.27	1.88	0.09	7.35	9.33	9.29	33.73
LKD-286	2.50	2.49	1.35	3.03	0.08	8.27	10.33	8.57	37.54
LKD-287	2.24	0.81	0.92	3.38	0.05	9.18	10.87	7.86	36.02
LKD-288	2.56	1.50	0.47	2.80	0.07	8.88	7.53	6.71	31.34
LKD-289	2.26	1.37	1.32	1.58	0.09	5.20	13.60	7.86	34.25
LKD-290	2.98	1.24	1.39	0.70	0.05	7.35	9.47	7.14	31.15
LKD-291	1.98	1.41	0.84	4.50	0.08	7.65	8.80	7.86	33.89
LKD-292	0.26	2.28	0.23	0.79	0.08	6.73	2.93	6.86	20.25
LKD-293	0.27	1.28	0.17	0.61	0.06	4.29	4.93	7.86	19.57
LKD-294	0.21	1.36	0.11	1.17	0.08	4.90	3.07	10.00	21.02
LKD-295	0.30	2.65	0.19	5.03	0.11	6.12	3.13	7.86	25.53
LKD-296	2.82	1.78	0.29	2.33	0.09	9.18	8.00	8.57	33.92
LKD-297	2.20	0.68	1.57	1.75	0.10	7.04	10.13	6.71	30.84
LKD-298	1.47	0.91	1.05	2.63	0.09	6.43	8.13	6.29	27.67
LKD-299	1.82	1.06	0.98	4.08	0.11	9.49	9.87	6.71	34.84
LKD-300	2.46	1.72	1.15	5.50	0.15	10.10	12.73	10.00	44.91
LKD-301	2.68	1.64	1.03	1.36	0.13	6.12	13.20	6.93	33.96
LKD-302	1.47	2.10	0.18	1.71	0.11	6.43	7.27	7.86	27.70
LKD-303	0.94	1.65	0.68	3.58	0.09	9.49	7.73	8.57	33.24
LKD-304	1.44	2.03	1.21	1.98	0.10	7.96	10.07	10.00	35.55
LKD-305	2.48	2.63	1.54	2.49	0.07	6.43	11.60	6.86	34.78
LKD-306	2.68	4.45	0.27	4.33	0.14	6.73	9.73	7.14	36.29
LKD-307	1.99	1.53	1.38	3.30	0.11	6.12	10.07	10.71	35.86

Continuation of Table S6. Heavy metal(loid)s Er and RI.

	Cu	Pb	Cr	Ni	Zn	Cd	As	Hg	
	Er	Er	Er	Er	Er	Er	Er	Er	RI
LKD-308	1.42	1.51	1.19	1.53	0.10	6.73	10.00	10.00	33.25
LKD-309	4.04	4.08	3.25	9.78	0.46	12.24	9.33	51.43	96.87

Table S7. Laboratory results.

Location ID	Analysis No.	Cu	Pb	Zn	Cr	Ni	Cd	As	Hg	Mn
		10^{-6}	10^{-6}	10^{-6}	10^{-6}	10^{-6}	10^{-6}	10^{-6}	10^{-6}	10^{-2}
1	B160050001	15.6	6.60	3.96	6.52	4.14	0.040	2.01	0.017	0.0130
2	B160050002	24.0	5.15	10.00	21.80	2.96	0.034	1.66	0.014	0.0093
3	B160050003	12.5	3.38	2.04	25.90	3.22	0.030	1.68	0.015	0.0130
4	B160050004	0.5	13.90	2.80	1.65	0.06	0.040	0.59	0.014	0.0031
5	B160050005	29.8	4.40	12.90	26.70	2.67	0.037	1.54	0.016	0.0100
6	B160050006	0.6	1.74	1.94	5.27	0.06	0.042	1.02	0.014	0.0015
7	B160050007	0.6	2.48	1.66	1.58	0.60	0.033	1.08	0.012	0.0150
8	B160050008	0.9	11.40	6.04	1.90	0.06	0.037	0.45	0.010	0.0056
9	B160050009	21.6	1.21	6.52	12.20	3.87	0.033	2.43	0.010	0.0100
10	B160050010	0.6	1.82	1.38	1.52	0.52	0.034	1.24	0.014	0.0130
11	B160050011	30.7	5.41	14.00	21.40	3.41	0.037	1.45	0.016	0.0190
12	B160050012	28.6	21.00	14.30	17.80	2.82	0.036	1.33	0.015	0.0085
13	B160050013	11.2	2.38	1.07	9.51	2.91	0.042	1.92	0.015	0.0093
14	B160050014	7.9	1.63	1.66	8.71	2.30	0.035	1.35	0.014	0.0093
15	B160050015	1.2	4.65	0.52	2.24	0.06	0.041	0.59	0.015	0.0023
16	B160050016	0.1	3.17	0.34	3.12	0.06	0.042	1.01	0.017	0.0015
17	B160050017	1.4	2.03	1.71	5.73	0.91	0.040	0.70	0.015	0.0015
18	B160050018	0.9	3.47	3.26	7.97	1.79	0.045	0.93	0.014	0.0039
19	B160050019	0.8	3.25	1.31	2.91	0.06	0.047	0.99	0.018	0.0023
20	B160050021	0.8	1.38	0.76	1.71	0.06	0.041	1.20	0.015	0.0015
21	B160050022	1.4	3.57	4.52	6.31	1.35	0.044	1.08	0.018	0.0023
22	B160050023	0.5	0.78	0.36	4.31	0.32	0.044	1.19	0.017	0.0008
23	B160050024	11.8	0.66	1.56	30.00	4.57	0.034	2.26	0.013	0.0150
24	B160050025	10.3	3.70	3.89	31.10	4.02	0.047	1.67	0.013	0.0190
25	B160050026	29.0	3.45	12.00	15.60	3.48	0.040	1.62	0.014	0.0100
26	B160050027	25.5	11.00	11.00	6.77	3.35	0.044	1.19	0.014	0.0110
27	B160050028	45.5	4.20	23.70	26.80	4.59	0.041	1.96	0.022	0.0220
28	B160050029	33.2	0.22	9.87	39.50	7.08	0.039	2.96	0.014	0.0100
29	B160050030	20.9	2.27	5.46	85.70	5.06	0.036	2.88	0.021	0.0120
30	B160050031	16.5	2.37	1.73	11.10	5.37	0.039	2.08	0.012	0.0150
31	B160050032	11.9	4.67	5.64	27.80	7.60	0.044	1.42	0.013	0.0170
32	B160050033	13.1	19.10	2.40	27.00	4.08	0.047	1.61	0.013	0.0120
33	B160050034	16.7	4.90	2.26	36.00	6.13	0.051	1.62	0.012	0.0160
34	B160050035	12.5	108.00	2.15	41.60	7.70	0.039	1.79	0.012	0.0110
35	B160050036	6.0	3.65	1.35	11.70	3.69	0.033	1.40	0.011	0.0170
36	B160050037	11.0	4.71	5.63	15.30	7.51	0.038	1.63	0.014	0.0160
37	B160050038	15.2	4.53	6.10	69.40	5.48	0.047	2.24	0.018	0.0120
38	B160050039	11.4	3.80	2.33	23.00	5.73	0.049	1.84	0.013	0.0110
39	B160050040	22.7	1.93	1.68	9.41	6.53	0.042	2.52	0.013	0.0150
40	B160050041	18.0	2.20	4.68	64.40	6.26	0.040	2.04	0.015	0.0170
41	B160050042	11.8	3.92	5.16	23.70	3.98	0.035	1.25	0.012	0.0093
42	B160050044	9.9	4.73	5.16	19.70	3.33	0.040	1.96	0.014	0.0093
43	B160050045	0.9	0.70	8.89	9.93	0.49	0.030	0.47	0.011	0.0008

Continuation of Table S7. Laboratory results.

Location ID	Analysis No.	Cu	Pb	Zn	Cr	Ni	Cd	As	Hg	Mn
		10^{-6}	10^{-6}	10^{-6}	10^{-6}	10^{-6}	10^{-6}	10^{-6}	10^{-6}	10^{-2}
44	B160050046	1.8	2.00	2.54	6.54	1.34	0.047	0.86	0.014	0.0015
45	B160050047	1.2	0.60	2.42	3.82	0.52	0.033	0.65	0.015	0.0008
46	B160050048	0.6	2.30	3.45	3.07	0.71	0.041	0.62	0.011	0.0023
47	B160050049	9.2	2.45	6.79	9.78	4.29	0.038	1.56	0.016	0.0110
48	B160050050	13.6	4.22	7.17	44.40	5.27	0.028	1.80	0.017	0.0260
49	B160050051	9.3	20.40	2.83	25.90	3.86	0.032	2.00	0.011	0.0190
50	B160050052	13.4	61.30	3.06	31.80	4.20	0.044	2.07	0.010	0.0160
51	B160050053	12.0	2.49	1.38	20.60	4.87	0.039	1.97	0.012	0.0110
52	B160050054	18.0	0.85	2.19	7.95	5.37	0.029	2.05	0.013	0.0210
53	B160050056	9.1	8.35	3.70	20.80	2.96	0.044	1.17	0.013	0.0150
54	B160050057	12.6	2.13	5.29	25.50	4.20	0.034	0.93	0.015	0.0120
55	B160050058	12.5	2.39	4.91	27.80	3.68	0.040	0.91	0.012	0.0120
56	B160050059	8.0	4.68	3.21	21.90	2.85	0.043	0.83	0.011	0.0150
57	B160050060	0.6	1.75	3.58	3.58	0.64	0.042	1.00	0.011	0.0008
58	B160050061	1.7	1.89	3.52	5.53	1.55	0.035	0.19	0.013	0.0015
59	B160050062	0.8	2.02	6.02	2.23	0.67	0.039	0.36	0.013	0.0015
60	B160050063	1.4	1.99	5.09	2.65	0.80	0.028	0.09	0.012	0.0015
61	B160050064	22.1	1.04	3.04	48.00	6.78	0.051	1.83	0.010	0.0150
62	B160050065	21.8	1.48	5.36	10.50	4.60	0.042	1.54	0.010	0.0130
63	B160050066	24.3	4.66	5.88	37.60	6.86	0.038	2.44	0.013	0.0150
64	B160050067	11.4	10.00	2.62	29.70	3.75	0.038	1.90	0.014	0.0170
65	B160050068	23.5	3.27	8.11	31.50	3.74	0.040	1.43	0.028	0.0260
66	B160050069	22.3	1.85	0.92	10.20	6.51	0.040	2.09	0.012	0.0190
67	B160050070	16.9	1.92	1.40	52.40	5.24	0.039	1.63	0.010	0.0180
68	B160050071	14.6	0.47	1.89	27.90	4.71	0.044	1.79	0.010	0.0150
69	B160050072	1.3	1.03	1.69	31.70	4.68	0.041	0.41	0.014	0.0015
70	B160050073	7.6	0.69	2.51	13.60	2.76	0.032	1.47	0.011	0.0140
71	B160050074	1.9	1.29	1.74	31.80	4.20	0.033	0.41	0.014	0.0015
72	B160050075	0.8	0.54	1.33	2.92	0.64	0.032	0.15	0.010	0.0008
73	B160050076	1.9	4.54	7.85	3.67	2.37	0.035	1.69	0.023	0.0039
74	B160050077	1.2	2.72	3.08	4.80	0.93	0.040	0.37	0.014	0.0031
75	B160050078	1.0	1.19	2.19	5.55	0.97	0.034	0.56	0.012	0.0023
76	B160050080	0.5	1.11	1.33	2.66	0.48	0.029	0.36	0.012	0.0015
77	B160050081	19.8	4.22	8.68	20.60	3.79	0.038	1.00	0.011	0.0110
78	B160050082	29.1	5.96	14.70	9.22	3.86	0.041	1.15	0.014	0.0150
79	B160050083	8.5	6.88	2.00	11.60	3.29	0.032	1.10	0.011	0.0085
80	B160050084	7.2	3.25	1.15	17.70	1.78	0.049	0.85	0.010	0.0085
81	B160050085	6.7	0.55	2.03	10.20	2.15	0.032	1.13	0.010	0.0170
82	B160050086	14.6	0.06	1.68	6.51	4.08	0.036	0.96	0.011	0.0120
83	B160050087	9.6	1.35	3.10	31.50	3.67	0.031	0.81	0.012	0.0120
84	B160050088	12.3	1.09	3.26	23.40	3.70	0.027	1.01	0.011	0.0085
85	B160050089	6.7	5.46	0.84	14.30	2.32	0.031	0.58	0.010	0.0085
86	B160050090	17.5	2.13	14.10	31.10	4.73	0.037	1.79	0.011	0.0180
87	B160050091	2.0	2.59	3.26	7.78	2.56	0.032	0.11	0.015	0.0008
88	B160050092	3.3	3.55	7.46	16.20	2.68	0.039	0.15	0.012	0.0031

Continuation of Table S7. Laboratory results.

Location ID	Analysis No.	Cu	Pb	Zn	Cr	Ni	Cd	As	Hg	Mn
		10^{-6}	10^{-6}	10^{-6}	10^{-6}	10^{-6}	10^{-6}	10^{-6}	10^{-6}	10^{-2}
89	B160050093	0.2	1.81	4.67	3.08	0.30	0.023	0.36	0.010	0.0015
90	B160050094	1.7	0.98	3.32	4.35	0.06	0.037	0.03	0.009	0.0015
91	B160050095	14.8	3.72	5.13	19.90	53.80	0.040	0.98	0.013	0.0085
92	B160050096	11.8	3.54	1.74	6.84	49.70	0.040	1.19	0.012	0.0074
93	B160050097	7.9	1.72	1.99	9.65	53.20	0.036	0.95	0.012	0.0120
94	B160050098	5.7	2.10	2.19	13.40	92.60	0.033	1.05	0.011	0.0070
95	B160050099	8.2	1.75	2.87	9.63	52.10	0.037	0.68	0.012	0.0150
96	B160050100	18.9	1.41	2.14	9.17	61.40	0.031	1.42	0.010	0.0160
97	B160050101	9.5	1.60	0.03	31.00	3.66	0.008	1.06	0.013	0.0150
98	B160050102	7.1	2.08	0.72	15.60	1.62	0.004	1.35	0.016	0.0077
99	B160050103	9.0	1.62	0.03	28.80	2.01	0.002	1.17	0.012	0.0110
100	B160050104	8.3	3.02	0.03	15.80	2.02	0.020	0.92	0.014	0.0150
101	B160050105	0.5	2.69	0.03	3.09	0.06	0.002	0.64	0.013	0.0015
102	B160050106	0.5	6.36	0.03	<0.3	0.06	0.023	0.67	0.013	0.0062
103	B160050107	0.4	9.17	0.83	2.98	0.06	0.002	0.20	0.016	0.0046
104	B160050108	2.1	1.90	0.03	2.71	0.06	0.002	0.18	0.015	0.0008
105	B160050109	7.2	6.12	0.03	12.30	0.91	0.015	0.80	0.014	0.0077
106	B160050110	12.1	7.30	1.15	6.21	2.59	0.014	1.36	0.016	0.0085
107	B160050111	9.6	1.93	0.03	9.77	14.80	0.031	1.21	0.013	0.0093
108	B160050112	4.4	2.40	0.03	14.20	2.70	0.007	1.21	0.014	0.0093
109	B160050114	9.5	1.56	1.50	15.40	6.84	0.002	1.28	0.013	0.0150
110	B160050115	11.9	1.84	1.55	4.12	4.20	0.018	1.41	0.014	0.0110
111	B160050116	11.2	2.56	7.28	29.60	3.31	0.023	1.14	0.011	0.0110
112	B160050117	6.7	3.56	0.33	13.20	2.03	0.005	0.83	0.014	0.0085
113	B160050118	33.1	3.18	14.50	18.20	1.79	0.002	1.37	0.012	0.0093
114	B160050119	9.1	3.06	0.03	16.20	2.04	0.021	1.22	0.012	0.0140
115	B160050120	6.5	2.07	0.36	13.00	1.77	0.006	0.60	0.014	0.0093
116	B160050121	0.8	9.27	1.57	4.52	7.05	0.003	0.27	0.015	0.0023
117	B160050122	0.8	7.35	0.03	1.94	1.75	0.024	0.54	0.015	0.0008
118	B160050123	1.1	9.76	3.02	4.15	0.58	0.010	0.15	0.014	0.0046
119	B160050124	8.6	6.32	3.88	15.60	2.52	0.006	1.09	0.016	0.0110
120	B160050125	6.6	2.34	0.03	6.18	1.35	0.022	1.26	0.014	0.0046
121	B160050126	9.9	5.53	7.66	25.80	3.72	0.013	1.77	0.016	0.0077
122	B160050127	6.4	1.96	0.03	16.50	3.07	0.013	1.38	0.015	0.0077
123	B160050128	6.0	1.65	0.03	11.50	2.53	0.029	1.08	0.013	0.0130
124	B160050129	13.0	2.21	0.03	4.44	3.48	0.023	1.63	0.011	0.0150
125	B160050130	9.5	1.82	0.03	28.20	1.80	0.020	1.52	0.014	0.0110
126	B160050131	7.1	3.38	0.35	11.80	2.10	0.026	1.30	0.017	0.0085
127	B160050132	8.5	2.34	0.03	20.00	1.50	0.018	1.79	0.012	0.0110
128	B160050133	11.4	1.65	1.58	25.60	2.86	0.003	0.48	0.013	0.0150
129	B160050134	0.6	4.24	0.03	1.88	0.06	0.013	1.83	0.015	0.0015
130	B160050135	0.8	5.71	0.03	0.64	0.06	0.032	0.72	0.012	0.0008
131	B160050136	1.5	9.39	4.58	5.09	2.07	0.002	0.13	0.013	0.0120
132	B160050137	0.0	10.50	3.54	2.65	0.76	0.002	0.54	0.015	0.0008

Continuation of Table S7. Laboratory results.

Location ID	Analysis No.	Cu	Pb	Zn	Cr	Ni	Cd	As	Hg	Mn
		10^{-6}	10^{-6}	10^{-6}	10^{-6}	10^{-6}	10^{-6}	10^{-6}	10^{-6}	10^{-2}
133	B160050138	8.8	3.02	2.69	18.80	2.50	0.011	1.26	0.014	0.0093
134	B160050139	10.5	3.20	2.62	9.77	3.53	0.011	1.46	0.012	0.0110
135	B160050140	8.9	3.68	1.96	23.90	3.22	0.002	1.67	0.014	0.0085
136	B160050141	10.8	4.43	1.09	23.20	4.95	0.035	1.57	0.011	0.0110
137	B160050142	10.4	4.77	3.85	32.20	5.73	0.010	1.20	0.015	0.0190
138	B160050143	16.1	6.36	6.16	8.68	5.54	0.007	1.75	0.012	0.0170
139	B160050144	9.8	1.79	0.03	31.60	3.13	0.022	1.38	0.010	0.0150
140	B160050145	7.3	2.84	2.22	12.80	2.26	0.022	1.04	0.012	0.0100
141	B160050147	7.9	2.13	3.01	13.60	1.88	0.008	1.57	0.012	0.0100
142	B160050148	8.3	2.57	0.93	11.60	2.42	0.014	2.25	0.011	0.0140
143	B160050149	0.5	4.52	1.19	3.82	0.06	0.002	0.59	0.011	0.0008
144	B160050150	0.7	5.99	4.38	1.85	0.06	0.018	0.50	0.013	0.0008
145	B160050151	0.1	6.18	3.40	0.35	0.06	0.002	0.79	0.011	0.0023
146	B160050152	0.2	7.58	4.44	0.35	0.06	0.005	0.21	0.013	0.0170
147	B160050153	12.2	0.06	0.03	32.60	3.29	0.002	2.07	0.011	0.0150
148	B160050154	5.1	1.45	0.03	0.70	1.89	0.002	0.97	0.012	0.0085
149	B160050155	6.9	2.04	4.27	10.80	3.72	0.002	1.42	0.015	0.0093
150	B160050156	4.1	0.88	2.02	7.35	1.40	0.006	0.75	0.017	0.0150
151	B160050157	5.9	1.15	0.03	14.20	2.61	0.002	0.94	0.014	0.0170
152	B160050158	14.5	0.06	0.03	1.59	4.30	0.002	1.84	0.017	0.0160
153	B160050159	12.5	0.59	0.03	40.80	4.54	0.002	1.48	0.014	0.0180
154	B160050160	15.0	1.57	2.84	30.80	3.96	0.002	2.12	0.015	0.0150
155	B160050162	4.7	0.20	2.34	9.24	0.96	0.062	1.35	0.012	0.0077
156	B160050163	7.5	7.13	3.17	21.20	2.96	0.003	1.34	0.012	0.0220
157	B160050164	0.1	3.13	1.83	0.35	0.06	0.003	0.31	0.015	0.0015
158	B160050165	<0.02	4.42	2.16	0.35	0.06	0.002	0.41	0.015	0.0008
159	B160050166	0.7	5.13	4.00	2.22	0.46	0.007	0.39	0.013	0.0046
160	B160050167	3.7	0.37	9.29	67.20	5.35	0.003	0.35	0.017	0.0130
161	B160050168	4.2	0.98	2.11	8.50	2.54	0.007	0.85	0.017	0.0120
162	B160050169	10.3	3.80	16.90	13.60	10.70	0.002	1.72	0.061	0.0120
163	B160050170	6.7	1.48	9.18	70.60	2.78	0.002	2.36	0.025	0.0100
164	B160050171	7.3	0.94	4.65	13.10	2.66	0.002	1.19	0.018	0.0190
165	B160050172	12.1	0.06	7.48	5.39	4.13	0.005	1.37	0.015	0.0130
166	B160050173	6.7	0.35	5.50	21.70	2.32	0.002	0.94	0.013	0.0093
167	B160050174	22.6	3.01	8.11	48.10	8.34	0.076	2.91	0.010	0.0250
168	B160050175	8.5	0.66	0.03	20.00	3.38	0.002	1.46	0.012	0.0120
169	B160050176	6.2	0.06	0.03	15.30	1.77	0.002	2.41	0.015	0.0140
170	B160050177	10.1	0.26	2.41	24.50	4.79	0.002	1.30	0.013	0.0230
171	B160050178	0.3	0.06	0.09	<0.3	0.75	0.002	0.36	0.015	0.0039
172	B160050179	0.5	2.21	1.21	0.35	<0.05	0.002	0.41	0.013	0.0077
173	B160050180	0.4	0.90	2.29	0.35	0.40	0.002	0.58	0.014	0.0093
174	B160050181	0.2	0.41	2.51	0.57	0.41	0.002	1.71	0.015	0.0046
175	B160050182	6.4	0.67	0.92	16.50	2.41	0.002	1.66	0.013	0.0160
176	B160050183	11.7	1.97	0.75	3.13	3.31	0.002	2.95	0.023	0.0150
177	B160050184	8.6	0.88	0.73	31.20	2.24	0.005	1.78	0.017	0.0120

Continuation of Table S7. Laboratory results.

Location ID	Analysis No.	Cu	Pb	Zn	Cr	Ni	Cd	As	Hg	Mn
		10^{-6}	10^{-6}	10^{-6}	10^{-6}	10^{-6}	10^{-6}	10^{-6}	10^{-6}	10^{-2}
178	B160050185	5.7	0.06	1.76	5.30	1.68	0.048	2.16	0.016	0.0077
179	B160050186	7.6	0.06	1.25	17.30	2.29	0.002	1.45	0.013	0.0120
180	B160050187	12.0	0.07	1.87	31.50	6.01	0.002	2.33	0.016	0.0120
181	B160050189	8.6	1.45	3.34	18.10	4.20	0.002	2.06	0.012	0.0120
182	B160050190	14.5	0.06	0.97	31.40	4.89	0.002	2.62	0.016	0.0140
183	B160050191	23.1	0.46	9.79	3.45	2.93	0.002	1.37	0.014	0.0130
184	B160050192	7.3	3.04	6.37	21.20	4.74	0.002	1.74	0.013	0.0180
185	B160050193	0.8	3.77	10.70	0.97	0.26	0.001	0.51	0.014	0.0130
186	B160050194	0.1	3.50	1.78	2.27	0.54	0.046	0.24	0.015	0.0031
187	B160050195	0.9	3.10	2.04	18.20	2.11	0.002	0.75	0.020	0.0039
188	B160050196	0.3	2.81	10.00	2.04	1.47	0.002	0.53	0.018	0.0031
189	B160050197	7.0	9.34	8.21	14.70	2.13	0.002	1.40	0.012	0.0160
190	B160050198	14.4	0.74	3.73	29.10	4.40	0.003	3.63	0.026	0.0140
191	B160050199	5.5	0.06	1.75	5.90	2.16	0.053	1.91	0.014	0.0100
192	B160050200	5.6	0.06	7.02	15.50	2.39	0.003	1.14	0.012	0.0077
193	B160050201	12.5	1.58	4.47	38.60	5.12	0.035	1.57	0.009	0.0120
194	B160050202	9.5	1.71	4.67	13.90	1.68	0.022	1.47	0.012	0.0140
195	B160050203	11.6	2.43	5.77	23.50	4.83	0.018	2.05	0.009	0.0120
196	B160050204	15.6	2.55	6.62	34.70	5.04	0.018	2.15	0.010	0.0110
197	B160050205	12.8	2.65	7.49	19.10	8.94	0.025	1.58	0.012	0.0120
198	B160050206	10.3	2.61	5.53	31.70	5.82	0.015	1.81	0.012	0.0120
199	B160050207	1.1	3.82	5.81	2.08	0.06	0.016	0.30	0.011	0.0031
200	B160050208	1.0	3.50	5.73	2.20	0.06	0.023	0.27	0.012	0.0015
201	B160050209	1.0	8.07	6.48	6.35	1.34	0.022	0.16	0.010	0.0039
202	B160050210	1.2	6.91	6.73	10.20	1.52	0.024	0.24	0.010	0.0031
203	B160050212	11.2	3.02	7.44	15.50	0.59	0.044	0.63	0.011	0.0110
204	B160050213	16.5	2.35	5.46	9.10	0.34	0.021	1.43	0.011	0.0120
205	B160050214	8.3	2.34	6.75	24.30	6.23	0.023	0.96	0.012	0.0110
206	B160050215	9.6	2.07	5.48	20.20	3.15	0.027	1.20	0.010	0.0093
207	B160050216	11.7	2.65	6.17	41.10	5.13	0.017	1.62	0.011	0.0110
208	B160050217	14.3	2.67	7.47	25.90	5.70	0.031	1.49	0.010	0.0200
209	B160050218	8.5	2.32	6.27	12.40	1.62	0.023	1.64	0.012	0.0093
210	B160050219	8.2	2.81	6.94	5.29	0.06	0.021	0.85	0.009	0.0100
211	B160050220	3.2	2.96	4.27	5.72	0.06	0.024	0.80	0.010	0.0070
212	B160050221	12.6	3.24	4.92	27.20	2.30	0.015	1.87	0.013	0.0110
213	B160050222	1.0	3.23	5.17	3.00	2.52	0.019	0.26	0.010	0.0015
214	B160050223	1.7	46.10	14.50	23.80	7.15	0.026	0.22	0.010	0.0093
215	B160050224	1.1	2.90	4.58	2.20	3.09	0.017	0.19	0.010	0.0015
216	B160050225	1.2	1.76	4.32	8.08	0.06	0.022	0.11	0.014	0.0023
217	B160050226	8.6	1.63	4.27	32.80	0.70	0.023	1.20	0.010	0.0190
218	B160050227	19.1	2.88	4.76	9.22	5.48	0.018	1.87	0.009	0.0160
219	B160050228	16.5	4.02	14.40	98.20	11.70	0.030	2.25	0.010	0.0140
220	B160050229	10.0	3.58	4.55	16.90	2.54	0.010	1.09	0.011	0.0120
221	B160050230	5.7	4.84	6.58	31.30	3.61	0.020	1.50	0.009	0.0100

Continuation of Table S7. Laboratory results.

Location ID	Analysis No.	Cu	Pb	Zn	Cr	Ni	Cd	As	Hg	Mn
		10^{-6}	10^{-6}	10^{-6}	10^{-6}	10^{-6}	10^{-6}	10^{-6}	10^{-6}	10^{-2}
222	B160050231	16.6	2.57	7.60	41.00	6.75	0.021	2.07	0.009	0.0210
223	B160050232	11.8	3.66	7.02	28.90	2.07	0.013	1.59	0.012	0.0130
224	B160050233	11.9	3.46	6.52	15.70	1.55	0.016	1.65	0.016	0.0150
225	B160050234	6.8	2.34	4.11	23.50	0.06	0.016	1.46	0.010	0.0100
226	B160050235	14.4	4.09	5.87	36.80	4.65	0.015	2.03	0.012	0.0190
227	B160050236	1.1	2.44	4.44	8.72	2.10	0.020	0.36	0.009	0.0039
228	B160050237	1.2	2.07	4.31	7.27	0.06	0.019	0.51	0.010	0.0015
229	B160050238	1.4	2.41	5.84	10.20	6.41	0.021	0.17	0.010	0.0031
230	B160050239	1.6	3.78	6.33	13.70	6.29	0.033	0.43	0.013	0.0063
231	B160050240	5.6	5.54	6.25	31.40	4.11	0.027	1.08	0.011	0.0150
232	B160050242	16.0	7.02	18.30	75.50	6.94	0.022	1.69	0.008	0.0170
233	B160050243	9.5	3.06	7.12	32.80	1.00	0.023	1.02	0.013	0.0120
234	B160050244	10.6	4.82	10.10	25.30	4.57	0.023	1.00	0.014	0.0120
235	B160050245	10.2	20.40	3.66	22.00	3.99	0.017	1.77	0.008	0.0170
236	B160050246	8.0	2.84	4.40	15.20	3.37	0.030	1.39	0.008	0.0093
237	B160050247	11.2	5.37	5.37	12.60	3.81	0.025	1.05	0.007	0.0150
238	B160050248	8.2	2.18	5.04	32.00	3.55	0.018	1.83	0.010	0.0085
239	B160050249	12.3	2.26	5.29	22.30	6.21	0.025	1.00	0.010	0.0110
240	B160050250	1.1	4.95	5.59	6.95	0.06	0.023	0.32	0.009	0.0039
241	B160050251	0.9	2.61	5.26	8.65	2.18	0.023	0.18	0.012	0.0023
242	B160050252	1.0	3.43	5.11	8.79	2.01	0.029	0.65	0.014	0.0008
243	B160050253	1.6	5.17	6.87	3.56	0.06	0.022	0.27	0.010	0.0031
244	B160050254	14.6	11.00	8.00	11.10	13.80	0.027	1.40	0.010	0.0180
245	B160050255	12.7	2.98	4.47	41.90	2.75	0.022	2.05	0.009	0.0150
246	B160050256	9.2	3.26	5.67	29.90	5.01	0.019	1.15	0.015	0.0220
247	B160050257	10.6	2.40	6.43	33.00	6.12	0.031	1.51	0.010	0.0130
248	B160050258	15.1	6.41	9.60	46.60	16.30	0.023	1.90	0.014	0.0190
249	B160050259	13.5	5.43	4.85	18.80	4.24	0.024	1.76	0.013	0.0120
250	B160050260	10.7	4.42	5.68	23.40	4.80	0.024	1.32	0.014	0.0120
251	B160050261	8.9	2.67	4.74	25.00	2.82	0.019	1.50	0.010	0.0130
252	B160050262	13.8	4.58	8.26	31.70	2.99	0.027	1.92	0.011	0.0120
253	B160050263	12.9	4.19	5.11	28.10	3.06	0.020	1.31	0.010	0.0120
254	B160050264	1.4	2.43	4.63	7.25	0.06	0.018	0.22	0.013	0.0015
255	B160050265	1.2	3.10	5.02	7.25	0.48	0.030	12.50	2.870	0.0054
256	B160050266	0.8	2.09	4.79	1.26	0.06	0.021	0.45	0.009	0.0011
257	B160050268	0.9	4.85	6.73	2.66	0.06	0.038	0.08	0.008	0.0023
258	B160050269	17.0	3.71	7.59	12.90	6.63	0.030	1.66	0.016	0.0160
259	B160050270	15.4	6.67	7.21	62.90	10.40	0.026	1.94	0.014	0.0260
260	B160050271	8.6	3.90	6.51	13.80	6.67	0.023	1.50	0.011	0.0160
261	B160050272	10.9	2.38	5.10	21.00	2.50	0.018	1.49	0.011	0.0140
262	B160050273	14.8	2.74	6.15	33.50	3.91	0.026	2.15	0.011	0.0170
263	B160050274	11.9	2.40	5.19	17.30	3.82	0.020	1.61	0.011	0.0120
264	B160050275	11.8	4.25	6.77	9.24	2.26	0.027	1.52	0.011	0.0130
265	B160050276	9.3	6.50	8.20	22.60	2.87	0.028	1.77	0.015	0.0130

Continuation of Table S7. Laboratory results.

Location ID	Analysis No.	Cu	Pb	Zn	Cr	Ni	Cd	As	Hg	Mn
		10^{-6}	10^{-6}	10^{-6}	10^{-6}	10^{-6}	10^{-6}	10^{-6}	10^{-6}	10^{-2}
266	B160050277	19.7	7.81	10.80	33.20	3.23	0.018	2.69	0.013	0.0190
267	B160050279	12.9	4.72	7.05	25.90	3.50	0.031	1.83	0.017	0.0130
268	B160050280	1.3	2.32	5.21	1.93	2.54	0.024	0.39	0.013	0.0015
269	B160050281	1.8	4.21	6.19	3.36	0.06	0.023	0.34	0.013	0.0023
270	B160050282	1.6	2.50	6.41	2.16	0.94	0.015	0.17	0.011	0.0015
271	B160050283	1.3	4.35	6.30	2.21	1.12	0.022	0.17	0.010	0.0120
272	B160050284	20.0	3.83	8.51	6.35	2.16	0.019	1.40	0.013	0.0160
273	B160050285	18.3	5.45	7.75	58.20	7.79	0.028	2.03	0.013	0.0220
274	B160050286	12.6	10.10	10.40	37.80	4.79	0.021	1.96	0.010	0.0230
275	B160050287	10.6	21.90	9.02	29.20	4.11	0.021	1.64	0.010	0.0220
276	B160050288	10.0	8.19	7.09	19.50	2.25	0.031	1.74	0.011	0.0160
277	B160050289	15.9	10.20	7.38	37.20	4.64	0.019	1.99	0.010	0.0150
278	B160050290	1.3	5.00	8.22	2.45	1.22	0.023	0.18	0.010	0.0120
279	B160050291	10.3	10.90	9.50	21.80	8.74	0.034	1.32	0.011	0.0170
280	B160050292	16.2	7.76	9.29	38.60	9.46	0.050	1.37	0.013	0.0120
281	B160050293	15.5	3.64	10.50	30.80	16.80	0.034	1.67	0.010	0.0150
282	B160050294	1.1	3.88	5.98	5.90	1.30	0.033	0.25	0.011	0.0012
283	B160050295	1.4	5.66	7.04	6.74	0.06	0.036	0.18	0.010	0.0027
284	B160050296	1.2	4.72	4.29	7.26	1.68	0.035	0.14	0.014	0.0008
285	B160050297	1.1	6.68	6.19	6.44	9.76	0.033	0.21	0.009	0.0023
286	B160050298	15.2	6.33	3.18	13.10	0.06	0.028	1.29	0.010	0.0120
287	B160050299	17.0	9.67	8.98	58.50	14.60	0.036	1.37	0.010	0.0140
288	B160050300	7.3	7.89	8.36	17.90	0.06	0.019	0.83	0.008	0.0120
289	B160050301	9.4	7.82	6.24	22.30	7.50	0.024	1.40	0.013	0.0110
290	B160050302	12.5	9.96	5.84	23.70	12.10	0.027	1.55	0.012	0.0140
291	B160050303	11.2	3.25	3.44	16.10	13.50	0.030	1.63	0.011	0.0110
292	B160050304	12.8	6.00	5.16	8.29	11.20	0.029	1.13	0.009	0.0120
293	B160050305	11.3	5.48	6.44	23.10	6.32	0.017	2.04	0.011	0.0140
294	B160050306	14.9	4.94	3.74	24.40	2.79	0.024	1.42	0.010	0.0120
295	B160050307	9.9	5.62	5.62	14.70	18.00	0.025	1.32	0.011	0.0120
296	B160050308	1.3	9.12	5.87	4.07	3.16	0.022	0.44	0.010	0.0008
297	B160050309	1.4	5.13	4.49	2.97	2.42	0.014	0.74	0.011	0.0015
298	B160050310	1.1	5.45	5.54	2.00	4.67	0.016	0.46	0.014	0.0015
299	B160050312	1.5	10.60	8.00	3.26	20.10	0.020	0.47	0.011	0.0031
300	B160050313	14.1	7.13	6.54	5.07	9.33	0.030	1.20	0.012	0.0150
301	B160050314	11.0	2.70	7.29	27.40	7.00	0.023	1.52	0.009	0.0120
302	B160050315	7.3	3.63	6.46	18.30	10.50	0.021	1.22	0.009	0.0150
303	B160050316	9.1	4.25	7.89	17.20	16.30	0.031	1.48	0.009	0.0100
304	B160050317	12.3	6.89	10.40	20.10	22.00	0.033	1.91	0.014	0.0180
305	B160050318	13.4	6.56	9.37	18.00	5.44	0.020	1.98	0.010	0.0120
306	B160050319	7.4	8.39	7.76	3.17	6.83	0.021	1.09	0.011	0.0130
307	B160050320	4.7	6.60	6.48	11.90	14.30	0.031	1.16	0.012	0.0093
308	B160050321	7.2	8.11	7.11	21.10	7.90	0.026	1.51	0.014	0.0170
309	B160050322	12.4	10.50	5.17	27.00	9.97	0.021	1.74	0.010	0.0093

Continuation of Table S7. Laboratory results.

Location ID	Analysis No.	Cu	Pb	Zn	Cr	Ni	Cd	As	Hg	Mn
		10^{-6}	10^{-6}	10^{-6}	10^{-6}	10^{-6}	10^{-6}	10^{-6}	10^{-6}	10^{-2}
310	B160050323	13.4	17.80	9.88	4.74	17.30	0.022	1.46	0.010	0.0130
311	B160050324	9.9	6.10	7.60	24.10	13.20	0.020	1.51	0.015	0.0120
312	B160050325	7.1	6.03	7.12	20.90	6.11	0.022	1.50	0.014	0.0170
313	B160050326	20.2	16.30	32.70	56.80	39.10	0.040	1.40	0.072	0.0140

Reference

1. Elster D, Holman IP, Parker A, Rudge L. An investigation of the basement complex aquifer system in Lofa county, Liberia, for the purpose of siting boreholes. Q J Eng Geol Hydrogeol. 2014;47(2):159–67.

2. Taylor SR, McLennan SM. The geochemical evolution of the continental crust. Rev Geophys. 1995;33(2):241–65.

3. Wedepohl KH. The composition of the continental crust. Geochim Cosmochim Acta. 1995;59(7):1217–32.

4. Turekian KK, Wedepohl KH. Distribution of the elements in some major units of the earth's crust. Geol Soc Am Bull. 1961;72(2):175–92.
